# PEMOCS: effects of a concept-guided, PErsonalized, MOtor-Cognitive exergame training on cognitive functions and gait in chronic Stroke—a randomized, controlled trial

**DOI:** 10.3389/fnagi.2025.1514594

**Published:** 2025-03-13

**Authors:** S. K. Huber, R. H. Knols, J. P. O. Held, M. Betschart, S. Gartmann, N. Nauer, E. D. de Bruin

**Affiliations:** ^1^Physiotherapy Occupational Therapy Research Center, Directorate of Research and Education, University Hospital Zurich, Zürich, Switzerland; ^2^Motor Control and Learning Group, Institute of Human Movement Sciences and Sport, Department of Health Sciences and Technology, ETH Zurich, Zürich, Switzerland; ^3^Rehabilitation Center Triemli Zurich, Valens Clinics, Zürich, Switzerland; ^4^Bellevue Medical Group, Zürich, Switzerland; ^5^Department of Health, OST – Eastern Swiss University of Applied Sciences, St. Gallen, Switzerland; ^6^Institute of Therapy and Rehabilitation, Kantonsspital Winterthur, Winterthur, Switzerland; ^7^Division of Physiotherapy, Department of Neurobiology, Care Sciences and Society, Karolinska Institutet, Stockholm, Sweden

**Keywords:** stroke, exergaming, rehabilitation, cognition, gait, dual-task

## Abstract

**Purpose:**

Motor-cognitive exergames may be beneficial for addressing both motor and cognitive residual impairments in chronic stroke, however, effective training schedules are yet to be determined. Therefore, this study investigates the effects of a concept-guided, personalized, motor-cognitive exergame training on cognitive functions and gait in chronic stroke survivors.

**Methods:**

In this single-blind, randomized, controlled trial, stroke survivors (at least six-months post-stroke and able to perform step-based exergaming) were allocated either to the intervention (usual care + concept-guided, personalized, motor-cognitive exergame training) or the control group (usual care only). Global cognitive functioning was primarily targeted, while health-related quality of life (HRQoL), cognitive functions, mobility, and gait were evaluated secondarily. Analyses were performed with linear-mixed effect models.

**Results:**

Effects on global cognitive functioning were non-significant, with no differences between responders (participants exhibiting a clinically relevant change) and non-responders (participants exhibiting no clinically relevant change). Among secondary outcomes, the mobility domain of the HRQoL questionnaire, intrinsic visual alertness, cognitive flexibility, working memory, and outdoor walking speed as well as swing width (unaffected side) showed significant interaction effects in favour of the exergame group.

**Discussion:**

Additional exergaming helped maintaining global cognitive functioning and showed encouraging effects in mobility and cognitive outcomes. Responders and non-responders did not differ in adherence, baseline values or age. Enhancing the frequency and intensity of sessions could unlock more substantial benefits. Adopting a blended therapy approach may be key to maximizing positive effects.

**Clinical trial registration:**

clinicaltrials.gov, identifier NCT05524727.

## 1 Introduction

Stroke is a major cause of long-term disability in adults (Katan and Luft, 2018; Johnson et al., 2019), with common sequelae being cognitive and motor impairments (Sun et al., 2014; Ursin et al., 2019). In many stroke survivors, these impairments reside in the long-term, which hampers their daily-life functioning, independence, psychological and health-related quality of life (Nakling et al., 2017; Hotter et al., 2018; Katan and Luft, 2018; Sennfalt et al., 2019). Cognitive impairments are highly prevalent in chronic stroke [up to 84% (Mahon et al., 2017)], with processing speed, executive functions, memory, and visuospatial functions being the domains most often affected (Cumming et al., 2013; Middleton et al., 2014). Nevertheless, rehabilitation of cognitive functions has been neglected for a long time (Jokinen et al., 2015; McDonald et al., 2019), and residual cognitive impairments are an important unmet need in chronic stroke survivors (Pollock et al., 2012; Hotter et al., 2018; Virani et al., 2020; Rudberg et al., 2021). Additionally, up to 80% of stroke survivors experience hemiparesis (Barker and Mullooly, 1997; Patten et al., 2004). Hemiparesis alters spatiotemporal gait parameters including shorter stride length, lower cadence, and higher asymmetry, which can reduce gait speed, walking efficiency, and mobility (Olney and Richards, 1996; Katan and Luft, 2018).

Post-stroke cognitive and motor impairments are linked, as they share structural and functional roots in the nervous system (Verstraeten et al., 2016; Ursin et al., 2019). Motoric-Cognitive Risk (MCR) syndrome, which has been associated with stroke (Verghese et al., 2013; Beauchet et al., 2018), describes the collective deterioration and mutual influence of cognitive functions and specifically gait. This raises the idea that cognitive and gait impairments could also be addressed together to improve collectively (Verstraeten et al., 2016; Li et al., 2018; Ursin et al., 2019; Montero-Odasso et al., 2020). The guided plasticity facilitation model (Kraft, 2012; Fissler et al., 2013) suggests that the combination of physical and cognitive training specifically triggers neuroplasticity, which is key in stroke rehabilitation (Maier et al., 2019). Combined motor-cognitive interventions may thus be a promising approach to address cognitive deficits and gait impairments in stroke (Herold et al., 2018; Yang and Wang, 2021). Confirming the theory, motor-cognitive interventions have been found superior in improving motor and cognitive functions in healthy older adults (Bamidis et al., 2015; Lauenroth et al., 2016; Levin et al., 2017; Wollesen et al., 2020; Rieker et al., 2022; Teraz et al., 2022) and neurological populations (Gavelin et al., 2020; Sun et al., 2021; Zhou et al., 2021).

Exergames are a promising type of combined motor-cognitive training (Huber et al., 2022a), as the gamified training fosters motivation and enjoyment, which was attributed with higher adherence (Sailer et al., 2017; Valenzuela et al., 2018; Yoong et al., 2024). Exergames have been suggested as adjunct to standard stroke rehabilitation, especially for long-term and repetitious therapy (Chan et al., 2022; Zhang et al., 2022; Wicaksono et al., 2023). Potentially beneficial effects of exergames on cognitive and physical functioning have been reported in several systematic reviews in healthy older adults (Stojan and Voelcker-Rehage, 2019; Fang et al., 2020; Pacheco et al., 2020; Janhunen et al., 2021; Suleiman-Martos et al., 2021; Yen and Chiu, 2021), as well as in neurological and general populations (Stanmore et al., 2017; Mura et al., 2018; Calafiore et al., 2021; Prosperini et al., 2021). In chronic stroke, exergames have been reported to beneficially affect motor functions including balance, mobility, and gait (Chan et al., 2022; Huber et al., 2022a; Ghazavi Dozin et al., 2024). However, effective training schedules remain to be determined, as exergame interventions in previous stroke studies were mainly implemented in an unstructured manner, while description of and rationales for exercise variables and personalized schedules were insufficient (Cano Porras et al., 2018; Stojan and Voelcker-Rehage, 2019; Huber et al., 2022a; Rüth et al., 2023). Regarding cognitive functions, a recent systematic review revealed that exergame trainings had a beneficial effect in the acute phase of stroke, while no effect was found for chronic stroke (Duta et al., 2023). Notably, the subgroup analysis in chronic stroke included only two studies with small sample sizes and short intervention durations (Duta et al., 2023). Both reported on the same exergame intervention, which was performed seated. However, step-based exergaming in a standing position may be most beneficial for improving cognitive functions and gait (Tahmosybayat et al., 2018; Manser and de Bruin, 2021; Hou and Li, 2022; Manser and de Bruin, 2024). Hence, the effects of (step-based) exergames on cognitive functions in chronic stroke should be further investigated. Besides cognitive functions, detailed spatiotemporal gait parameters, real-world walking, and dual-task functions are underrepresented outcomes in studies investigating exergames (Gallou-Guyot et al., 2020; Huber et al., 2022a; Stretton et al., 2022). To address these research gaps, we developed an evidence-based concept for PErsonalized MOtor-Cognitive exergame training in chronic Stroke [PEMOCS, (Huber et al., 2024b)]. The aim of this study was to evaluate the effects of exergame training guided by the PEMOCS concept on cognitive functions, health-related quality of life, single- and dual-task walking mobility, as well as indoor and outdoor gait in community-dwelling chronic stroke survivors.

## 2 Materials and methods

### 2.1 Trial design

The PEMOCS study was a single-blind, parallel, randomized, controlled trial in three hospitals / rehabilitation centres in Canton Zurich (Switzerland). Participants signed written informed consent before any study procedures started. After the baseline assessments (T0), participants were 1:1 randomly allocated to the intervention group (usual care + concept-guided, personalized, motor-cognitive exergame training) or the control group (usual care only). After the 12-week intervention period, post-intervention assessments (T1) were performed, followed by a 12-week follow-up period (usual care only in both groups), and the follow-up assessments (T2). The initial protocol was adhered to during the trial. The study protocol was approved by the ethics committee of Canton Zurich (KEK Zürich, Switzerland), registered on clinicaltrials.gov (NCT05524727), and published (Huber et al., 2024a). CONSORT 2010 guidelines were followed for reporting (Schulz et al., 2010).

### 2.2 Randomization and blinding

Randomization was stratified by sex (Sohrabji et al., 2017; Dong et al., 2020) and cognitive impairment absent or present [MoCA score ≥ 24 or < 24, respectively (Chiti and Pantoni, 2014; Shi et al., 2018; Potocnik et al., 2020)]. It was performed using the randomization module within REDCap (Harris et al., 2009), which was used for the electronic case report forms (eCRF). A senior researcher, who was not involved in the study otherwise, created the randomization list following the instructions provided by REDCap (2018). Outcome assessors were blinded to group allocation, while care providers and participants were not. Care providers enrolled participants and assigned them to the groups.

### 2.3 Participants

Eligible were adult, chronic stroke survivors (≥ 18 years old, ≥ 6 months post-stroke), who were able to stand for 3 min, walk 10 m without personal assistance [Functional Ambulation Category ((Mehrholz et al., 2007), FAC ≥ 3)], follow a two-stage command, and give informed consent as documented by own signature. Excluded were people who were unable or unwilling to give informed consent, suffered from other neurological diseases except for cognitive deficits or dementia, presented clinical contra-indications against the study intervention, were unable to perform the study intervention or the primary outcome test [Montreal Cognitive Assessment (Nasreddine et al., 2005), MoCA], or were overlappingly enrolled in another clinical trial. All study visits including assessments and training sessions took place in the study centres.

### 2.4 Interventions

The intervention group received concept-guided, personalized, motor-cognitive exergame training additionally to usual care. The exergame training was performed twice per week for 30–40 min over 12 weeks, resulting in 840 min of total training time (Huber et al., 2024b). The control group received no additional intervention to usual care, but weekly phone calls, to balance for contact to the study team. These phone calls consisted of 5- to 10-minute conversations covering the questions to gather further activity outcomes (see below). Among these further activity outcomes, amount, intensity and content of each participant’s usual care was inquired as this can vary considerably from patient to patient. Depending on type and severity of remaining impairments (among other determinants), usual care for chronic stroke survivors in Switzerland can range from no therapies to several physical, occupational and / or speed therapy sessions (usually lasting 45 min) a week.

**Figure 1:**
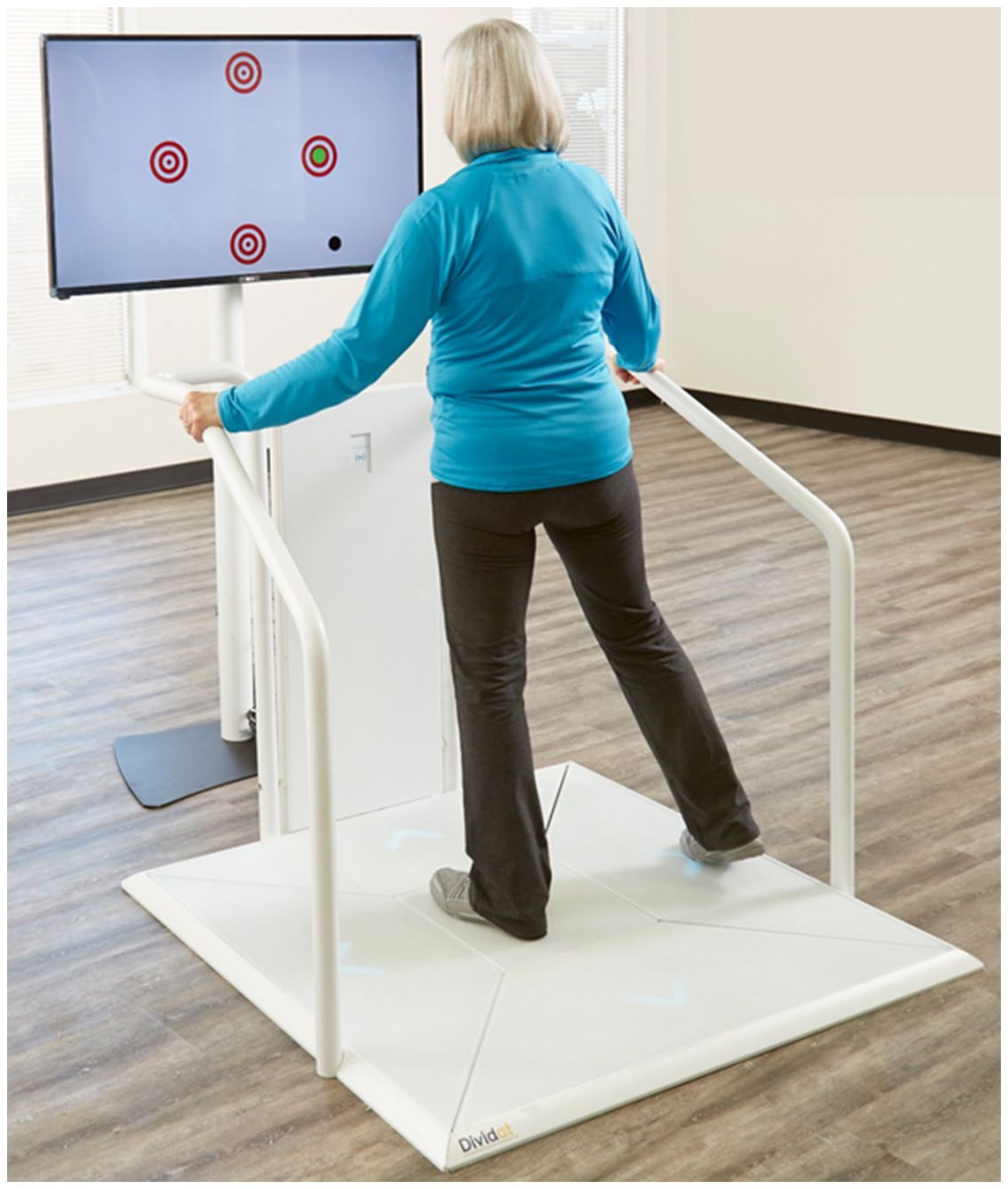
Study device in action: the Dividat Senso including pressure-sensitive plate, handrails in use, and the screen showing targets, one of the motor-cognitive exergames.

A concept for PErsonalized MOtor-Cognitive exergame training in chronic Stroke (PEMOCS) was specifically developed for this study and detailed elsewhere (Huber et al., 2024b). It has the aim to provide an optimal exergame training load for inducing neuroplasticity, cognitive and motor learning in each individual participant (Huber et al., 2024b). In short, the PEMOCS concept is based on Gentile’s Taxonomy for Motor Learning (Gentile, 1987), which was extended by a cognitive dimension for the use in exergame interventions. The taxonomy is a table of skill-categories and difficulty levels, to which the different games and game versions were assigned. By focusing on the most impaired cognitive domain of each individual, and applying individualized progression, the PEMOCS concept enables the personally tailored application of an exergame intervention (Huber et al., 2024b). Participants received one-to-one training sessions, which were planned individually and supervised by trained movement scientists of the study team. The motor-cognitive exergame training was delivered using the exergame system “Dividat Senso” (Dividat AG, Schindellegi, Switzerland, Figure 1). It comprises of a pressure-sensitive plate with handrails on three sides, which is placed in front of a screen on eye level. Cognitively challenging video games are presented on the screen, and stepping tasks are performed on the plate to interact with the games. Different games and game settings were available to train various cognitive and motor functions (Huber et al., 2024b).

### 2.5 Outcomes

All primary and secondary outcomes were collected at the three measurement time points (T0, T1, T2) by blinded assessors. Study data were collected and managed using REDCap electronic data capture tools hosted at ETH Zurich (Harris et al., 2009; Harris et al., 2019). REDCap (Research Electronic Data Capture) is a secure, web-based software platform designed to support data capture for research studies, providing (1) an intuitive interface for validated data capture; (2) audit trials for tracking data manipulation and export procedures; (3) automated export procedures for seamless data downloads to common statistical packages; and (4) procedures for data integration and interoperability with external sources. For a more detailed description of all outcomes and their conduct, see the protocol paper, which also presents definitions of all outcome variables (Tables 3, 4 in Huber et al., 2024a). The primary outcome was global cognitive functioning assessed by the total score of the Montreal Cognitive Assessment [MoCA (Nasreddine et al., 2005)]. The MoCA is a structured interview comprising of sub-tests assessing attention, executive functions, working memory, short-term memory recall, visuospatial skills, and orientation (Chiti and Pantoni, 2014). To prevent learning effects, three different versions of the test exist, which were used at the three measurement time points in this study.^1^ Blinded assessors were certified for conduction of the MoCA test (see text footnote 1).

Secondary outcomes were health-related quality of life, cognitive functions including alertness, processing speed, executive and visuospatial functions, single- and dual-task mobility, as well as indoor and outdoor walking. Participants filled the German Stroke Impact Scale (SIS) 3.0, which covers eight domains (strength, memory/thinking, emotion, communication, ADL/IADL, mobility, hand function, and participation) and the perceived state of recovery [0 to 100% (Duncan et al., 1999)]. Computer-based cognitive tests were performed in the Vienna Test System (VTS, Schuhfried GmbH, Mödling, Austria), including a simple reaction test [SRT (Zimmermann and Fimm, 1992; Sturm, 2006; Spikman and van Zomeren, 2010), “WAFA” within the VTS], the Trail-making test [TMT (Reitan, 1958; Tombaugh, 2004; Bowie and Harvey, 2006), “TMT – Langensteinbacher Version” within the VTS], the Stroop Interference test [(Faria et al., 2015; Scarpina and Tagini, 2017), “STROOP” within the VTS], the 2-back test [NBT (Owen et al., 2005; Schellig and Schuri, 2009; León-Domínguez et al., 2015; Gajewski et al., 2018), “NBV” within the VTS], and a mental rotation test [MRT (Shepard and Metzler, 1971), “3D” within the VTS]. The Timed-up-and-go test was performed in single-task (TUG) and dual-task (TUG-Cogn) mode (Ng and Hui-Chan, 2005; Yang et al., 2016; Ng et al., 2017). As cognitive task, participants subtracted three from a random number between 50 and 100 and not part of the row of three (Yang et al., 2016; Pumpho et al., 2020). To strive real dual-task (simultaneous) performance, participants were instructed to try and not prioritize one task over the other (“Please neither stop walking nor stop calculating throughout the whole trial.”). The cognitive single-task was performed until they reached 0 or until 60 s were complete (Huber et al., 2024a). To assess gait, a 10-Meter Walk Test [10MWT (Cheng et al., 2020)] and an Outdoor Walking Assessment [OWA (Huber et al., 2022b)] were performed. The 10MWT was performed indoors at preferred and fast walking speed. The OWA was performed on a flat 400-meter outdoor route without stairs walking at a preferred walking speed. All gait assessments were timed using a stopwatch, while inertial gait sensors (Physilog^®^ sensors, Gait Up SA, Lausanne, Switzerland) measured spatiotemporal gait parameters during the 10MWT preferred and the OWA. From the measured parameters, the gait variability index (Gouelle et al., 2013) for both legs, the asymmetry index (Anker, 2019), and the walk ratio (Bogen et al., 2018) were calculated. The gait variability index was determined following published instructions (Gouelle et al., 2013) (Supplementary material S1 and R scripts in https://doi.org/10.5281/zenodo.14849242). The walk ratio was calculated using the formulas in Bogen et al. (2018).

Intervention outcomes included compliance (completed sessions/offered sessions) and adherence rates (completed training time/offered training time), reasons for not attending or aborting training sessions, as well as the ratings of perceived motor-cognitive task difficulty and perceived performance provided in the training sessions. Further activity outcomes were inquired weekly (intervention group: at one of the study appointments, control group: during the phone calls) to gather information on physical and cognitive therapies (usual care), as well as physical and cognitive leisure activities. Participants reported on how many days per week, for how long, and at what perceived intensity they had performed the therapies and activities (Huber et al., 2024a).

Baseline factors included demographics, further participant characteristics, stroke diagnosis details, and several clinical characteristics, namely the Functional Ambulation Category [FAC (Mehrholz et al., 2007)], the initial and current National Institute of Health Stroke Scale [NIHSS (Kwah and Diong, 2014)], the Lower Extremity component of the Fugl-Meyer Assessment [FMA-LE (Fugl-Meyer et al., 1975)], and the Berg Balance Scale [BBS (Blum and Korner-Bitensky, 2008)]. The Charlson Comorbidity Index [CCI (Charlson et al., 2022)] was determined from self-reported health data. The initial NIHSS was collected from patient files, or if unavailable, retrospectively determined using the algorithm presented in Williams et al. (2000).

### 2.6 Sample size

The target sample size was 38 participants, determined by an a-priori sample size estimation based on existing systematic reviews on the effects of exergame and motor-cognitive training interventions on global cognitive functions in stroke and comparable populations (Zhu et al., 2016; Stanmore et al., 2017; Gavelin et al., 2020; Soares et al., 2021; Yen and Chiu, 2021; Yu and Chan, 2021). A small to medium effect size (*f* = 0.21) for global cognitive functions was anticipated and the following parameters entered in G*Power; *α*-level = 0.05, power = 0.80, number of groups = 2, number of measurements = 3, correlation among rep measures = 0.5, non-sphericity correction = 1. Withdrawals were replaced with further recruitment.

### 2.7 Statistical methods

Statistical analyses were performed using RStudio open-source software [Boston, United States, (RStudio_Team, 2020), Version 4.3.1] and Microsoft Excel (Microsoft Corporation, 2016). Data were explored, checking on distributions with the Shapiro–Wilk test (Field et al., 2012). Appropriate descriptives were obtained for all variables. Differences between groups at baseline were tested using independent t-tests (for interval data allowing parametric testing), Wilcoxon rank-sum tests (for interval data requiring non-parametric testing), Chi-square tests, or Fisher exact tests [for ordinal / categorical data with frequencies >5 or ≤ 5, respectively (Kim, 2017)]. For intervention outcomes, means overall and per week were calculated. For further activity outcomes, within participant means over the intervention and follow-up periods were calculated and compared between the two groups using independent *t*-tests or Wilcoxon rank-sum tests.

Interaction effects of interval data were analysed using linear mixed-effects models (LMEM, lmer-function of the lme4 package in R). Models were built by starting with a basic model including main fixed effects (group, time) and the interaction effect (group*time). Potential covariates (for choice, see below) were added first individually and then combined (Meteyard and Davies, 2020). The basic and covariate models were compared using the anova-function in R to choose the final model for each outcome [model with lowest AIC and BIC (Akaike and Bayesian Information Criteria, respectively) values (Meteyard and Davies, 2020)]. LMEMs have been recommended for data analysis of intervention studies despite frequent violation of assumptions in such data sets and have been reported to be sufficiently robust to smaller sample sizes (*N* < 50) and violated assumptions (Wiley and Rapp, 2019; Schielzeth et al., 2020). Nevertheless, the following assumptions were tested; (1) homogeneity of variance using Levene’s test, (2) linearity using ZRESID/ZPRED plots, (3) normality of residuals using the DHARMa package in R (Harting, 2022) and visually inspecting histograms of the residuals, (4) normality of random-effects by visually inspecting histograms of the random-effects, and (5) multicollinearity by calculating the variance inflation factor (VIF) (Field et al., 2012). In case severe violation of one or several assumptions was detected, these violations were reported alongside with the model results. Log-transformation of the data in violated models was considered, however, it was remarked that back-transformed model coefficients (b, standard errors) and effect sizes were not comparable to the original data and other models, as described in Feng et al. (2014). Therefore, no further action was taken in case of assumption violations. Final models, information on the process of model choice, as well as results of the assumption tests were documented in supplements (Meteyard and Davies, 2020). Effect sizes for the models were calculated as *r* (Bravais-Pearson correlation coefficient) = sqrt(t2 / (t2 + df)) (Field et al., 2012).

Ordinal outcomes were analysed with LMEMs of the family “poisson” (glmer-function of the lme4 package in R). Before analysis, clear outliers that could be associated to problems with handling the computer-based tests were replaced with the next higher / lower value ± 1 (Field et al., 2012). R scripts for all LMEMs are available online at https://doi.org/10.5281/zenodo.14849242.

All primary and secondary outcomes were primarily analysed following the intention-to-treat (ITT) principle. In these ITT analyses, missing data due to withdrawals were imputed using the last-observation-carried-forward method. Single missing data points (e.g., one assessment was not performed because of technical issues), were not imputed, as LMEMs can also be fitted with some missing data (Field et al., 2012; Gabrio et al., 2022). In case, a participant lacked data for an outcome at two or three time points, they were excluded from these analyses. Secondarily, per-protocol analyses were performed excluding individuals who withdrew from the study during the intervention period (T0–T1) or attended less than 85% of the total training sessions / time [for rationale, see Huber et al. (2024a)].

Potential covariates in the LMEMs were pre-defined as follows; (1) any baseline characteristic exhibiting a difference between groups, (2) parameters, which are established moderators of treatment effects and predictors of outcomes after stroke in literature. Therefore, age was considered as covariate in all analyses (Falck et al., 2019; Saa et al., 2021). Years of education was chosen for the secondary cognitive outcomes (Ojala-Oksala et al., 2012; Basak et al., 2020; Umarova et al., 2021; Hua et al., 2022). It was not a covariate in the analysis of the primary outcome, as the MoCA considers years of education for the total score (Nasreddine et al., 2005). Secondly, the FMA-LE score was chosen for the gait and mobility outcomes (Kollen et al., 2005; Burke et al., 2014; Rech et al., 2020; Selves et al., 2020). Sex and cognitive impairment were not considered as these variables were accounted for by the randomization stratification. Moreover, time post-stroke, which is often reported to be a moderator / predictor of post-stroke outcomes, was not considered for two reasons. Firstly, most studies that report time since stroke to be a moderator / predictor discriminate between (sub-)acute and chronic stroke (Lam et al., 2010; Burke et al., 2014; Prat-Luri et al., 2020), however, in our study, all participants were chronic. Secondly, there is an ongoing debate whether time post-stroke in the chronic stage is really a moderator of treatment effects (Rogers et al., 2018), and two recent studies found no moderating effect on treatment effects, and no correlations between time since stroke and spatiotemporal gait parameters, respectively (Gama et al., 2017; Zeng et al., 2023).

Within-group changes were tested using Wilcoxon signed rank tests (wilcox.test function in R, setting ‘paired = TRUE’) and effect sizes were calculated as *r* (Bravais-Pearson correlation coefficient) = z / sqrt(N) (Field et al., 2012). Additionally, responder analyses were performed for the MoCA, and those outcomes that would exhibit a significant interaction effect (Snapinn and Jiang, 2007). For a responder analysis, participants are divided into responders (exhibiting changes greater than a minimal clinically important difference, MCID) and non-responders (showing no clinically important change), and ratios thereof are compared between groups. To potentially identify characteristics of responders, we additionally compared baseline and covariate (see above) values of responders and non-responders in the intervention group with a Wilcoxon rank sum test.

Significance for all analyses was set to *p* < 0.05. No *p*-value adjustments for multiple testing were performed as this study had one primary outcome, while secondary analyses were rather exploratory (Feise, 2002). Bravais-Pearson correlation coefficients (*r*) were interpreted as small (*r* < 0.3), medium (0.3 ≤ *r* < 0.5), and large (*r* ≥ 0.5) (Field et al., 2012).

### 2.8 Changes to protocol

No changes to the initial protocol were made in terms of study procedures (Huber et al., 2024a), however, the following changes were made in the analysis. (1) A responder analysis was also performed for the MoCA, even though no significant interaction effect was found. (2) Gait variability was reported as the Gait Variability Index [GVI, (Gouelle et al., 2013), Supplementary material S1] instead of individual stride time / length variability. The GVI is a composite score of gait variability, which considers the nine most relevant parameters for gait variability (Gouelle et al., 2013). This has the advantage that one composite score replaced nine parameters, which may even add small changes in individual variability parameters, which would not be detected individually (Gouelle et al., 2013). (3) In the NBT, the reaction time of the mistakes (RT_mistakes_) was no longer an outcome. The N in the analysis became very small, as several participants made no mistakes in this test. As RT_mistakes_ is less relevant than other outcomes of the test (main outcomes: number of omissions and mistakes, reaction times of the correct answers (Schellig and Schuri, 2009), it was removed. (4) For the TUG-Cogn, it was not possible to report the cognitive dual-task effect due to a protocol limitation we only noticed when analysing the data. Particularly, the time given for the cognitive single-task should have been matched to participants’ time to complete the dual-task (Yang et al., 2016). However, participants were given 60 s to complete the single task. This time mismatch led to unrealistic values when calculating the cognitive dual-task effect. Therefore, it was decided to report single- and dual-task response rates separately instead of the cognitive dual-task effect. The motor task and dual-task effect were not affected by this protocol limitation. (5) Participants who performed the OWA twice were not excluded from the OWA analyses, as fewer missing data was present than expected. This way, valuable collected outcome data was considered for the analysis instead of being excluded.

## 3 Results

### 3.1 Participant flow and recruitment

**Figure 2:**
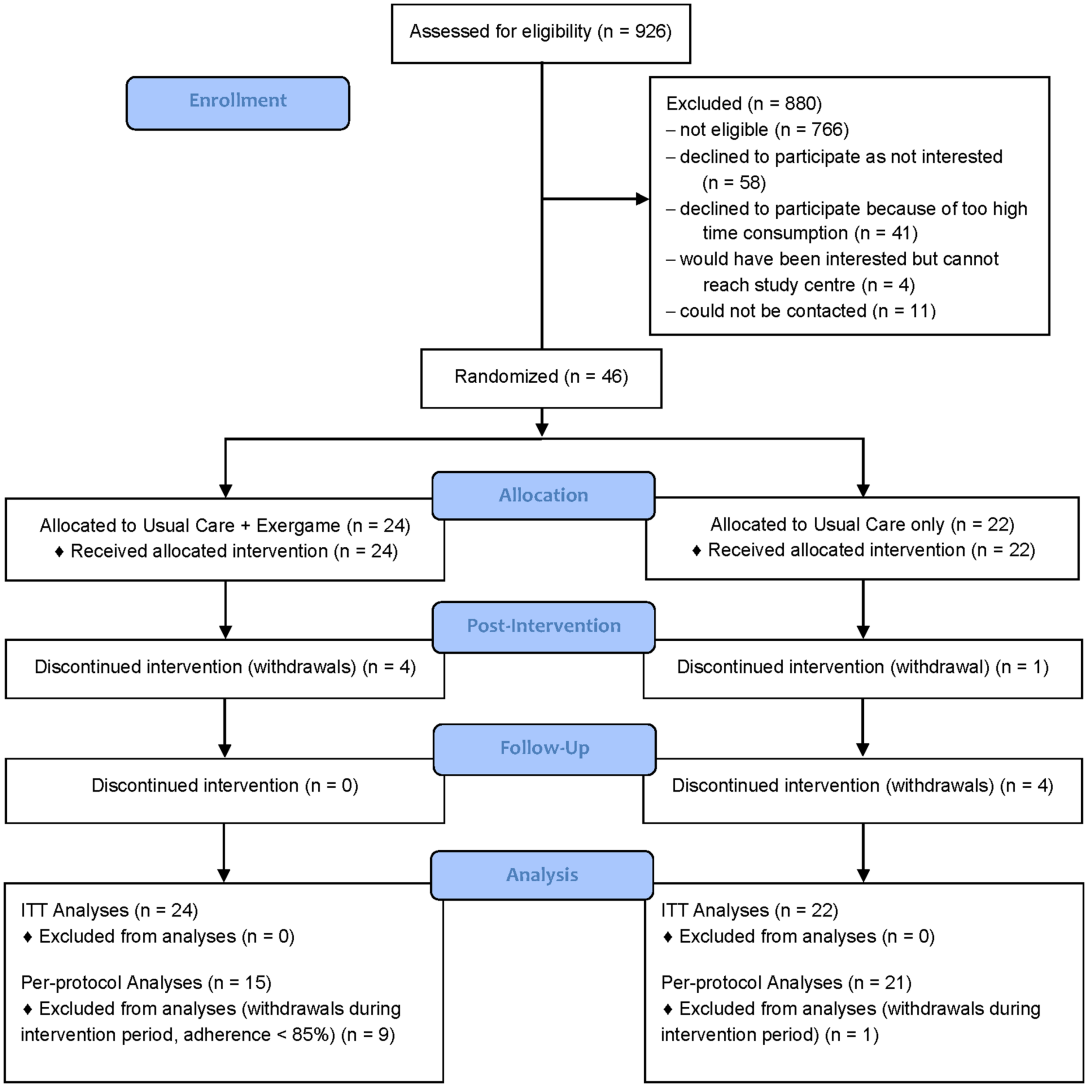
CONSORT flow diagram.

Recruitment ran between September 2022 and October 2023, and data collection was completed by April 2024. The study was completed as planned. Of 926 screened stroke survivors, 46 were included. The most frequent reason for non-inclusion was ineligibility (*N* = 766). Reasons for not participating among those who would have been eligible were “not being interested in study participation” (*N* = 58), “too high time consumption of the study” (*N* = 41), and “would be interested but cannot reach the study centre” (*N* = 4, all women). Women were underrepresented in all phases of recruitment and inclusion, namely screening on eligibility (ratio women / men: 0.73), contact for participation (ratio women / men: 0.43), and inclusion (ratio women / men: 0.24). Twenty-four and twenty-two participants were allocated to the intervention group and the control group, respectively. Forty-one participants completed the intervention period and thirty-seven the whole study duration, resulting in an attrition rate of 19.6%. In the intervention group, four participants withdrew during the intervention period (T0–T1), and none in the follow-up period (T1–T2). Reasons for withdrawal were (1) a temporally but not causally related adverse event (compare ‘Adverse events’, *N* = 1), (2) an unrelated serious adverse event (*N* = 1), (3) remarking that scheduling the training sessions in daily life was not possible (*N* = 1), and (4) not reported (withdrew without giving a reason, *N* = 1). In the control group, one participant withdrew during the intervention period (T0–T1), and four in the follow-up period (T1–T2). Reasons for withdrawal were (1) not reported (withdrew without giving a reason, *N* = 1), (2) boredom and / or discomfort with the phone calls (*N* = 3), and (3) an unrelated serious adverse event (compare ‘Adverse events’, N = 1). Intention-to-treat analyses included 46, per-protocol analyses 36 participants. Three participants were excluded from the SIS analyses, as they withdrew before initially answering the questionnaire. One participant was excluded from the TUG-Cogn, MRT, Stroop, and NBT analyses as he was not able to perform these assessments. Three participants were excluded from 10MWT-fast analyses, and three from the OWA analyses, because they did not want to perform the assessment. The participant flow is shown in Figure 2.

### 3.2 Baseline data

Participants were on average 66 years old. Men were over-represented in the study (women: 9 / men: 37), however, both sexes were equally stratified over both groups. Approximately, a fifth of participants (19.6%) had suffered a haemorrhagic stroke, and there was a wide range of stroke chronicity in the sample (6–222 months post-stroke). Participants were highly educated (15 years of education, range: 8–24). Most participants showed neither cognitive nor physical impairments at baseline. However, 13 were cognitively impaired [MoCA <24, (Chiti and Pantoni, 2014)], 3 showed reduced motor function [FMA-LE < 21, (Kwong and Ng, 2019)] and 6 had restrictions in community ambulation [gait speed ≤0.78 m/s, (Bijleveld-Uitman et al., 2013)]. Baseline characteristics are summarized in Table 1. Median values per group of all outcomes at the three time points are shown in Supplementary Tables S1–S3.

**Table 1:** Baseline characteristics of both groups and between group differences.

	Intervention group (*N* = 24)			Control group (*N* = 22)			Group difference
Characteristic	*N*	Mean ± SD / median (IQR)	Range: min-max / frequency	N	Mean ± SD / median (IQR)	Range: min-max / frequency	*p*-value
Sex	24		F: 5, M: 19	22		F: 4, M: 18	> 0.99^c^
Age [years]	24	68.75 ± 8.51	49–81	22	63.18 ± 9.69	46–77	0.05^a^
Weight [kg]	22	79.45 ± 10.36	61–100	21	76.38 ± 12.46	54–100	0.39^a^
Height [cm]	22	174.68 ± 7.55	154–185	21	175.33 ± 7.28	165–188	0.77^a^
Handedness	22		Left: 2, Right: 20	21		Left: 3, Right: 18	0.87^c^
Civil status	22		Single: 1 Married: 16 Divorced: 5 Widowed: 0	21		Single: 2 Married: 14 Divorced: 3 Widowed: 2	0.65^c^
Education [years]	24	14.17 ± 3.12	8–23	22	15.89 ± 3.89	11–24	0.11^a^
Education grade	24		Primary: 0 Secondary: 3 Professional: 9 Maturity: 3 University: 9	22		Primary: 0 Secondary: 0 Professional: 7 Maturity: 3 University: 9	0.29^c^
CCI [0–28]	22	2.5 (2,3)	1–5	21	2 (1,3)	1–5	0.42^b^
Stroke type	23		Ischemic: 19 Haemorrhagic: 4	22		Ischemic: 17 Haemorrhagic: 5	0.86^c^
Time since stroke [months]	23	27 (10,49.5)	6–222	22	21.5 (10,40.5)	6–179	0.60^b^
Previous stroke	24		No: 22, Yes: 2	21		No: 19, Yes: 2	0.80^c^
Affected brain hemisphere	24		Right: 14, Left: 9 Mid / both: 1	22		Right: 10, Left: 11 Mid / both: 1	0.77^c^
iNIHSS [0–42]	21	5 (2,7)	1–22	21	3 (1,8)	0–20	0.30^b^
Affected body side	24		Left: 15, Right: 9	22		Left: 10, Right: 12	0.37^c^
cNIHSS [0–42]	24	0 (0,1)	0–8	22	0 (0,1)	0–5	0.99^b^
mRS	22		0: 7, 1: 9 2: 3, 3: 2	21		0: 7, 1: 8 2: 4, 3: 2	> 0.99^c^
FAC	24		3: 1, 4: 2, 5: 21	22		3: 0, 4: 3, 5: 19	0.89^c^
FMA-LE [0–34]	24	31 (28,33)	13–34	22	31.5 (25,33)	14–34	0.69^b^
BBS [0–56]	24	55.5 (53,56)	19–56	22	56 (54,56)	40–56	0.47^b^
Faller	22		No: 18, Yes: 4	21		No: 16, Yes: 5	> 0.99^c^
Gait speed	24	1.38 (1.14,1.44)		22	1.25 (1.07,1.43)		0.56
MoCA, cogn. Impairment	24	25 (23, 27)	MoCA <24: 7 MoCA ≥24: 17	22	26 (24,28)	MoCA <24: 6 MoCA ≥24: 16	0.62

Between-group tests: ^a^independent *t*-test, ^b^Wilcoxon rank-sum test, ^c^Fisher’s exact test. BBS, Berg balance scale; CCI, Charlson comorbidity index; c/iNIHSS, current / initial National Institutes of Health Scale; FAC, Functional ambulation category; FMA-LE, Fugl-Meyer Assessment, lower extremity component; MoCA, Montreal Cognitive Assessment; mRS, modified Rankin scale; IQR, interquartile range; SD, standard deviation.

### 3.3 Outcomes and estimation

#### 3.3.1 Primary outcome

**Figure 3:**
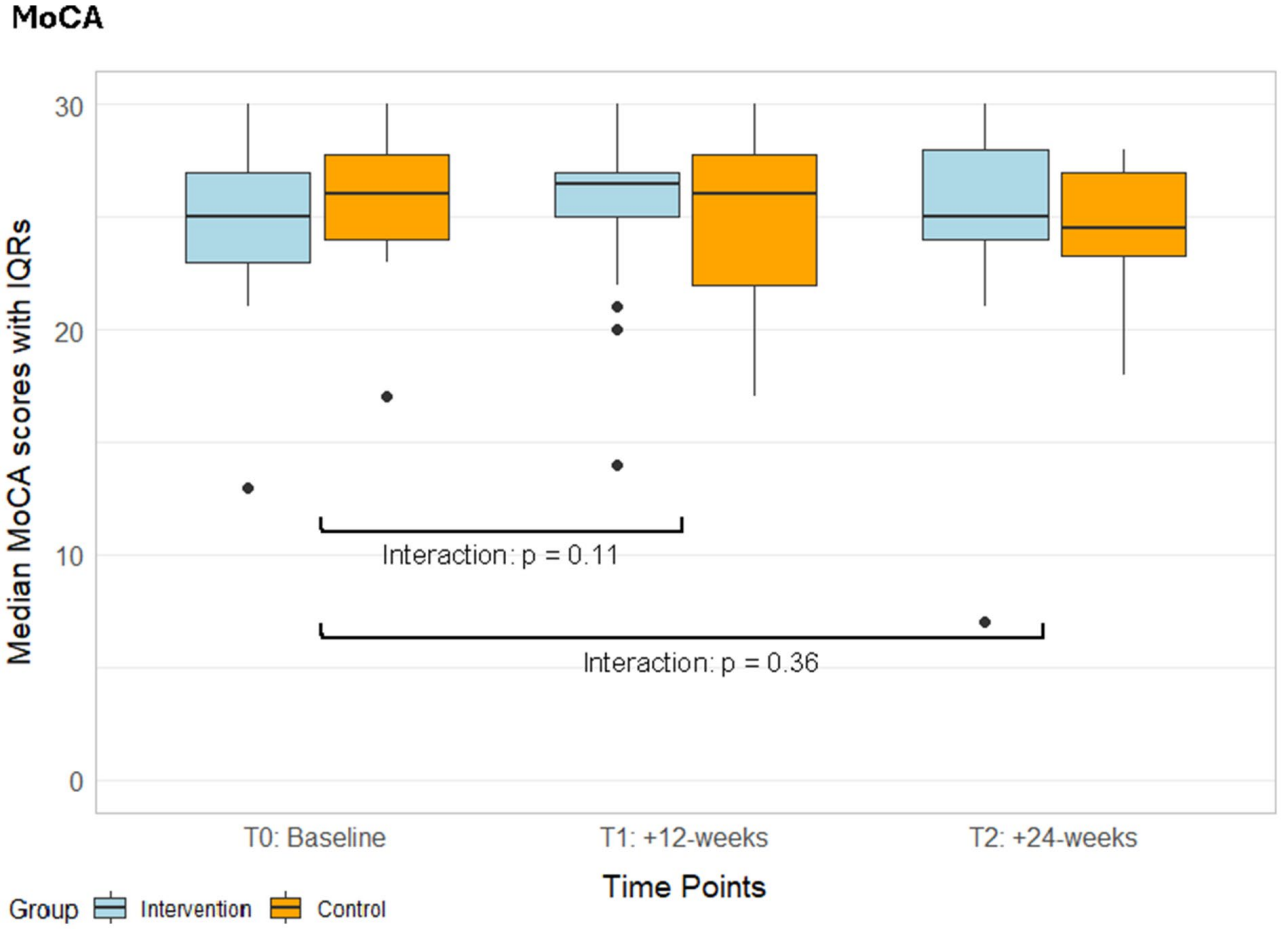
Median MoCA scores with IQRs from the ITT analyses of both groups over time. IQR, Interquartile ranges; MoCA, Montreal Cognitive Assessment; T1, Post-intervention measurements; T2, Follow-up measurements.

The MoCA total score remained stable in both groups, and there were no interaction effects (Figure 3, Supplementary Tables S4, S5, S10). The responder analysis was performed with 1.22 points as MCID (Wu et al., 2019). The rate of responders in the intervention group (responders / non-responders: 0.40 / 0.60) was higher compared to the control group (responders / non-responders: 0.20 / 0.80, Table 2). It did not considerably change when only adhering participants were considered (responders / non-responders: 0.44 / 0.56, Table 2). There was no significant difference between responders and non-responders in baseline MoCA score (*p* = 0.35) or age (*p* = 0.94, Table 2).

**Table 2:** Results of responder analyses.

		MoCA		SIS mobility		OWA gait speed	
	# Total	# Responders	Rate	# Responders	Rate	# Responders	Rate
Control group	20						
Responders		4	0.20	5	0.25	3	0.15
Non-responders		16	0.80	15	0.75	17	0.85
Intervention group	20 (Adh. 16)						
Responders		8	0.40	9	0.45	4	0.20
Adherer		7	0.44	6	0.38	3	0.19
Non-responders		12	0.60	11	0.55	16	0.80
Adherer		9	0.56	10	0.63	13	0.81

		Median (IQR)	*p*-value	Median (IQR)	*p*-value	Median (IQR)	*p*-value
Identification of possible responder characteristics							
Baseline	Responders	24 (23,24.5)	0.35	86 (67,92)	**<0.001**	1.3 (1.2,1.4)	0.74
	Non-respon.	26 (23,28)		100 (97,100)		1.3 (1.3,1.4)	
Age	Responders	67 (65,75)	0.94	73 (66,76)	0.32	69 (59,76)	0.70
	Non-respon.	71 (65,75)		68 (65,71)		69 (66,73)	
FMA-LE	Responders	–	–	–	–	30 (26,33)	0.77
	Non-respon.	–		–		31 (28,33)	

#, number of…; Adh, adherers; FMA-LE, Fugl-Meyer Assessment, lower extremity component; IQR, Interquartile range; MoCA, Montreal Cognitive Assessment; Non-respond, Non-responders; OWA, Outdoor walking assessment; SIS, Stroke Impact Scale. Bold values signify significant difference.

#### 3.3.2 Secondary outcomes

**Figure 4:**
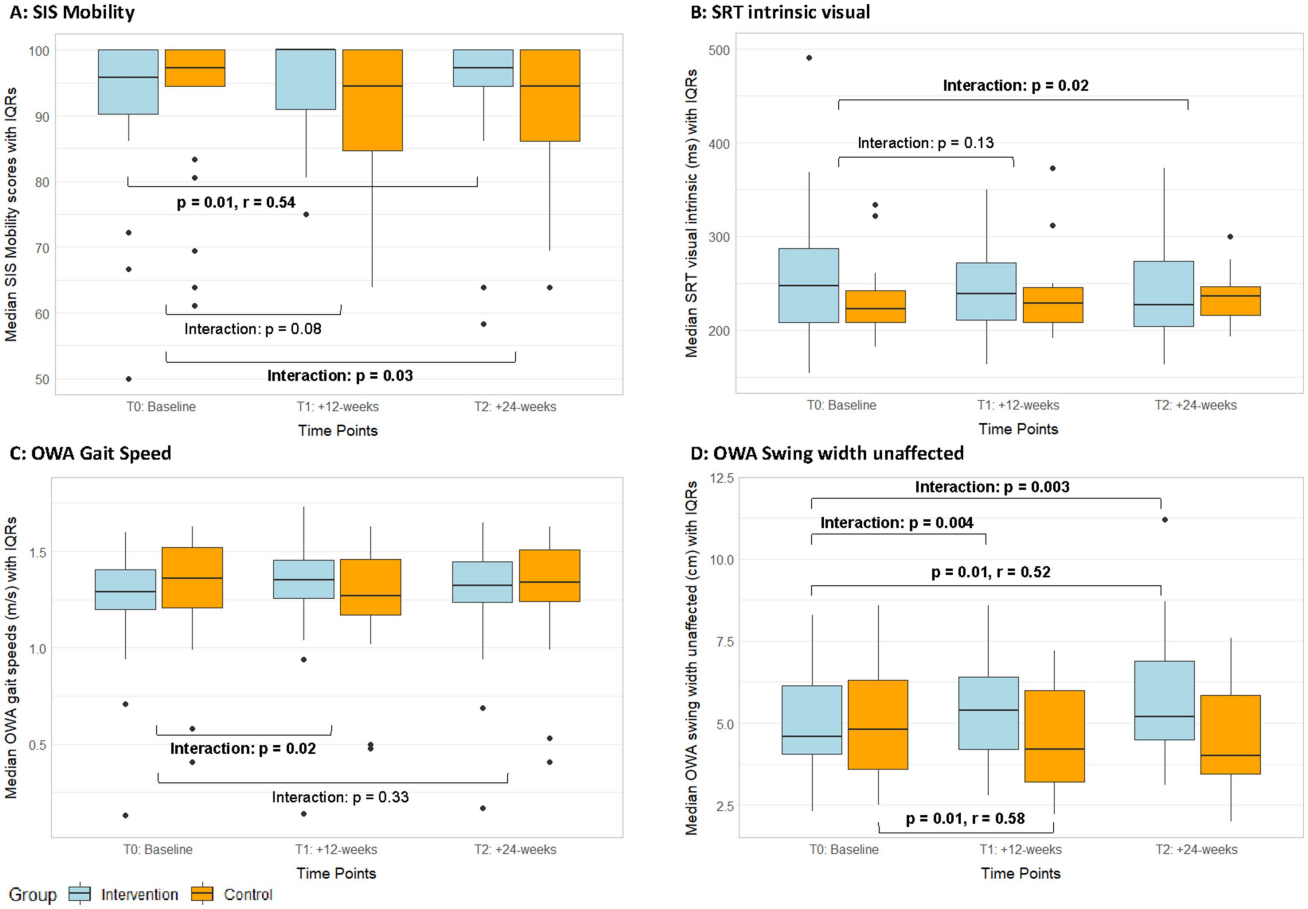
Median SIS-mobility scores **(A)**, reaction times in intrinsic visual alertness **(B)**, outdoor gait speed **(C)**, and outdoor swing width values **(D)** with IQRs from the ITT analyses of both groups over time. IQR, Interquartile ranges; OWA, Outdoor Walking Assessment; SIS, Stroke Impact Scale; SRT, Simple Reaction Test; T1, Post-intervention measurements; T2, Follow-up measurements.

The SIS domain Mobility showed a significant interaction effect (T2, ITT: *p* = 0.03, *r* = 0.24; PP: *p* = 0.06, *r* = 0.23; Figure 4A, Supplementary Tables S6, S7). The exergame group reported a significant improvement in perceived mobility with a large effect size, while the control group showed no changes in this domain (Table 3, Supplementary Table S10). The responder analysis was performed using 4.5 points as MCID (Lin et al., 2010). It showed that the rate of responders in the exergame group (responders / non-responders: 0.45 / 0.55) was higher compared to the control group (responders / non-responders: 0.25 / 0.75, Table 2). Responders showed significantly lower (*p* < 0.001) SIS Mobility values at baseline, while there was no difference in age (*p* = 0.32, Table 2).

**Table 3:** Overview of within-group improvements in both groups.

	Improvements within intervention group		Improvements within control group	
	T0 ➔ T1	T0 ➔ T2	T0 ➔ T1	T0 ➔ T2
MoCA	PP: +1 pt. NS	NS	PP: -1 pt. NS	NS
SRT	Phasic RT visual ITT: M, PP: L	Phasic RT visual ITT: NS, PP: L		
		Phasic RT auditory ITT: NS, PP: L	Phasic RT auditory ITT & PP: M	Phasic RT auditory ITT & PP: M
TMT	NS	NS	NS	NS
Stroop			Reading interference RT ITT: M, PP: L	
NBT	Mistakes ITT: M, PP: L	Mistakes ITT: M, PP: L		
MRT	Accuracy ITT & PP: L	Accuracy ITT & PP: L		Accuracy ITT & PP: M
SIS		Mobility ITT & PP: L		
TUG	Time single-task ITT & PP: L		Time single-task ITT & PP: M	
TUG-Cogn	Time dual-task ITT: M, PP: NS		Time dual-task ITT & PP: M	Time dual-task ITT & PP: L
				Dual-task effect motor ITT & PP: L
	CRR single-task ITT & PP: L	CRR single-task ITT & PP: L	CRR single-task ITT & PP: M	CRR single-task ITT & PP: L
	CRR dual-task ITT: M, PP: NS	CRR dual-task ITT: L, PP: NS		
10MWT		Gait speed ITT: M, PP: NS		
				Cadence ITT: NS, PP: M
	Stride length affected ITT: M, PP: NS	Stride length affected ITT: M, PP: NS		
	Stride time unaffected ITT & PP: L			
	Time fast ITT & PP: L	Time fast ITT: M, PP: NS	Time fast ITT & PP: M	
OWA	Cadence ITT: L, PP: NS	Cadence ITT: M, PP: NS		
	Stride time affected ITT: M, PP: NS	Stride time affected ITT: L, PP: NS		
	Stride time unaffected ITT: M, PP: NS	Stride time unaffected ITT: M, PP: NS		
		Swing width unaffected ITT: L, PP: NS		

Not mentioned variables showed maintenance, while none showed a deterioration in neither group. Median within-group changes in the majority of the variables were very small and may not be clinically relevant (compare Supplementary Tables S4–S9). CRR, Correct response rate; ITT, Intention-to-treat analysis; L, large / M, medium effect size; NS, non-significant; PP, Per-protocol analysis; Colours: green, large effect size / yellow, medium effect size in at least one analysis.

**Figure 5:**
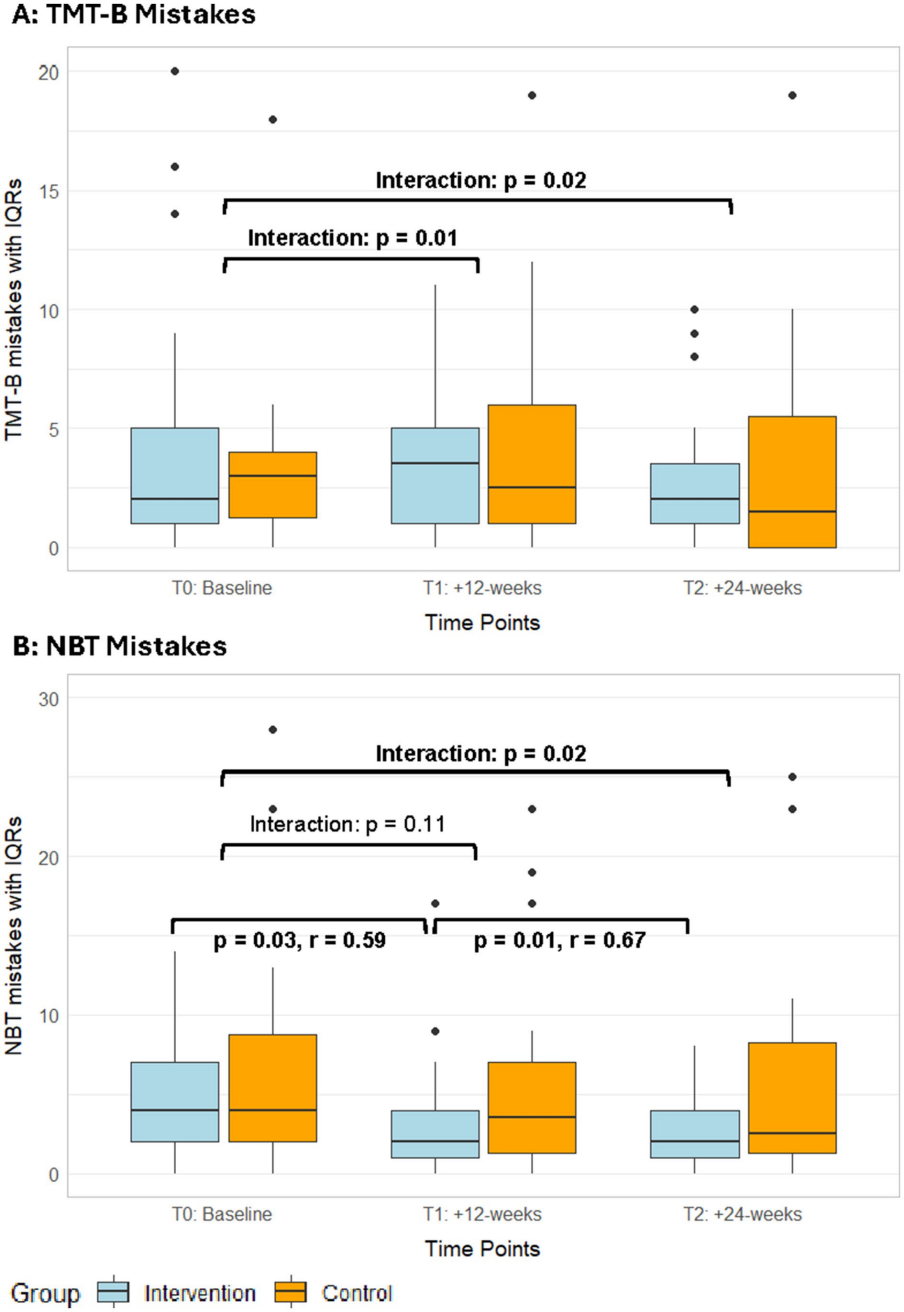
Median mistakes in the TMT-B **(A)** and NBT **(B)** with IQRs from the PP analyses of both groups over time. IQR, interquartile ranges; NBT, N-back test; TMT, trail-making test; T1, post-intervention measurements; T2, follow-up measurements.

Among the secondary cognitive assessments, a significant interaction effect in favour of the exergame group was found (Supplementary Tables S4, S5) in reaction time in the SRT—intrinsic visual alertness (T2, ITT: *p* = 0.02, *r* = 0.26; PP: *p* = 0.04, *r* = 0.25, Figure 4B). Additionally, the per-protocol analyses showed significant interaction effects in favour of the exergame group for mistakes in TMT-B (T1, *p* = 0.01; T2, *p* = 0.02, Figure 5A, Supplementary Table S5) and in mistakes in the NBT (T2, *p* = 0.02, Figure 5B, Supplementary Table S5). No responder analyses were performed for these outcomes, as no clinically relevant changes could be identified. In the MRT, both groups significantly improved in accuracy with large (exergame group) and medium (control group) effect sizes, respectively (Table 3, Supplementary Table S10).

The TUG(-Cogn) revealed no significant interaction effects, however, several significant within-group changes with medium to large effect sizes, which demonstrate performance improvements in single- and dual-task mobility in both groups (Table 3, Supplementary Table S10). In the 10MWT, there were no significant interaction effects for any parameter (Supplementary Tables S8, S9). In the 10MWT-fast, both groups significantly improved with medium to large effect sizes (Table 3, Supplementary Table S10). In outdoor gait speed, there was a significant, small interaction effect in favour of the exergame group (T1, ITT: *p* = 0.02, *r* = 0.25; PP: *p* = 0.11, *r* = 0.20, Figure 4C, Supplementary Tables S8, S9). The responder analysis was performed with 0.175 m/s as MCID (Fulk et al., 2011). It showed that the rate of responders in the exergame group (responders / non-responders: 0.2 / 0.8) was higher compared to the control group (responders / non-responders: 0.15 / 0.85, Table 2). OWA gait speeds at baseline, age and baseline FMA-scores in responders and non-responders were not significantly different (Table 2). Additionally, a significant interaction effect with a medium size favouring the exergame group was found for swing width unaffected measured outdoors (T1, ITT: *p* = 0.004, *r* = 0.31; PP: *p* = 0.02, *r* = 0.29 / T2, ITT: *p* = 0.003, *r* = 0.33; PP: *p* = 0.007, *r* = 0.33, Figure 4D, Supplementary Tables S8, S9). Furthermore, the exergame group showed significant improvements in gait speed, stride length affected and stride time unaffected indoors as well as in cadence, stride time affected, and stride time unaffected outdoors, while the control group showed a significant improvement only in cadence indoors (Table 3, Supplementary Table S10).

**Figure 6:**
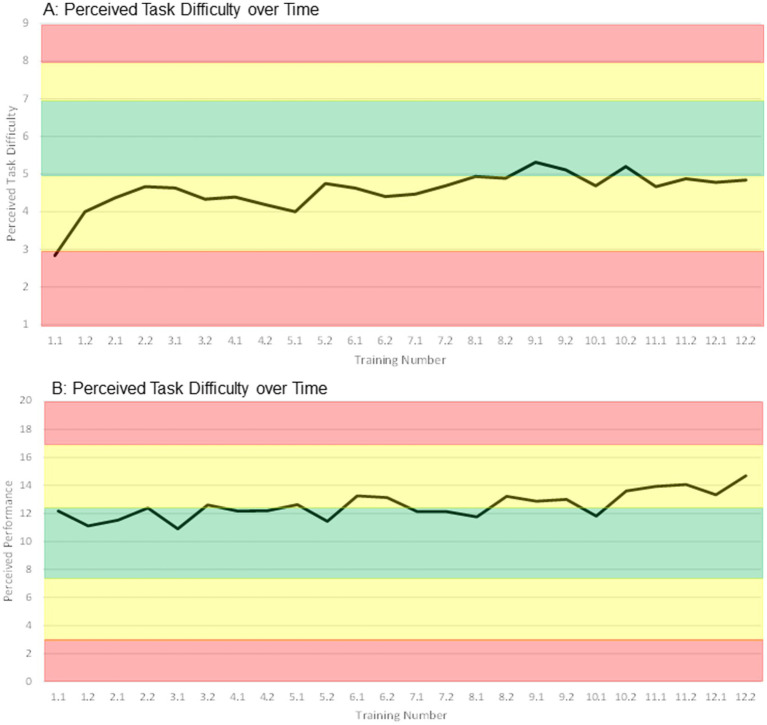
Median ratings of perceived task difficulty **(A)** and perceived performance **(B)** of all participants over time. Training number: *week.session*, e.g., 2.2 → week 2, second training in this week.

Intervention and further activity outcomes: Mean compliance (89.8%) and adherence (89.1%) rates were high (Supplementary Table S11, considering participants who completed the study). Reasons for not attending or aborting training sessions were all intervention-unrelated, including sickness, holidays, working appointments, or having forgotten the appointment. The mean perceived task difficulty of the participants started low, moved below the targeted range in weeks 3 to 6, and at the lower edge of the targeted range in weeks 7 to 12 (Figure 6A). The mean perceived performance started within the targeted range, moved at its upper edge throughout weeks 4 to 10, and raised higher in the last 2 weeks of the intervention (Figure 6B). Further activity outcomes showed that 18 participants received no usual care throughout the study, while the remaining participants received on average (median) 38 min of therapy per week (Supplementary Table S11). Ten participants received both physical and cognitive therapy, 12 received physical therapy only and 2 received cognitive therapy only. Overall, the average amount of physical therapy was higher compared to the average amount of cognitive therapy (Supplementary Table S11). In leisure time, participants were highly physically and cognitively active (median: 180–300 min of activity on 3–5 day per week, Supplementary Table S11). None of the further activity outcomes showed differences between the groups (Supplementary Table S11).

### 3.4 Safety

A mild and possibly (temporally but not causally) related AE was recorded in a participant with pre-existing heart condition. This participant experienced an uncomfortable but tolerable feeling in the chest / heart region during the more exhaustive parts of the measurement and training sessions, which decomposed after stopping the activities. The participant reported experiencing the same feelings when performing moderately to highly intense activities in daily life, but nevertheless withdrew. Another mild and unlikely related AE occurred in a participant, who experienced mild to moderate (tolerable) back pain during a few training sessions. The participant reported that the pain had been present already before the study and fluctuated, which was not attributable to the training sessions (pain was not stronger after the sessions). The training was adapted to the participant’s condition from the day of reporting; jumps were no longer integrated, and the cognitive load was reduced to enable focus on core stability during exercises. Furthermore, four unrelated SAEs (hospitalisations, for which a causal relationship to the study could be ruled out) and several unrelated AEs (e.g., flu, study-unrelated pain) occurred.

## 4 Discussion

This single-blind, randomized, controlled trial investigated the effects of a concept-guided, personalized, motor-cognitive exergame training (PEMOCS) added to usual care compared to usual care alone on global cognitive function, health-related quality of life (HRQoL), cognitive functions, single- and dual-task walking mobility, as well as indoor and outdoor gait in community-dwelling chronic stroke survivors. Global cognitive functioning (MoCA, primary outcome) remained stable in both groups, albeit the rate of responders showing a MCID in this outcome was higher in the exergame compared to the control group. The Mobility domain of the HRQoL questionnaire, reaction time in intrinsic visual alertness, mistakes in the TMT-B and the 2-back test, and gait speed as well as swing width on the unaffected side in outdoor walking showed significant interaction effects favouring the exergame group. However, as the study was not powered to these secondary outcomes, these results should be interpreted prudently. Neither the MoCA nor any of the outcomes showing significant interaction effects exhibited clinically meaningful changes on group level. Participants showed high compliance and adherence to the exergame training, and no definitely related adverse events occurred. The task load in the exergame training was below the targeted range.

### 4.1 Effects on cognitive outcomes

We found no interaction effects in the MoCA (Figure 3), however, a higher responder rate in the exergame compared to the control group (Table 2). Visual simple reaction time showed an interaction effect favouring the exergame group (Figure 4B). Additionally, the exergame group exhibited more and larger within-group improvements in cognitive assessments compared to the control group (Table 3). It seems that cognitive improvements were measurable with specific and sensitive tests but did not appear in a less sensitive summary score of global cognition as the MoCA. These findings are partly in line with three RCTs implementing exergame trainings in chronic stroke survivors with comparable baseline characteristics (Rozental-Iluz et al., 2016; Hung et al., 2017; Maier et al., 2020). Two of these studies compared exergame trainings in a standing position to conventional balance / physical training and found significant within-group improvements in the exergame groups but no significant interaction effects (Rozental-Iluz et al., 2016; Hung et al., 2017). The third study implemented seated video-game training including gross arm movements compared to conventional cognitive training, and found neither within-group changes nor interaction effects (Maier et al., 2020). These results align with findings in older adults that exergames should be performed in a standing position to improve cognitive functions (Tahmosybayat et al., 2018; Manser and de Bruin, 2021; Hou and Li, 2022; Manser et al., 2024). Moreover, they also align with evidence that supports exergames as adjunct to usual care or substitute to physical trainings but does not suggest them to outperform conventional cognitive trainings for improving specific cognitive functions (Stanmore et al., 2017; Mura et al., 2018).

Recent systematic reviews present mixed evidence regarding the effect of exergames on global and specific cognitive functions. Some found no beneficial effects on global cognitive functions of VR interventions including exergames in chronic stroke (Gao et al., 2021), and of exergames in neurological populations and healthy older adults (Mura et al., 2018; Chen et al., 2023; Li et al., 2023). Others, investigating the effects of exergames compared to active or passive control groups in acute stroke and older adults with or without cognitive impairments, did find significant beneficial effects on MoCA scores (Soares et al., 2021; Buyle et al., 2022; Cai et al., 2023). Attentional functions have been reported to significantly improve with exergames in stroke (Gao et al., 2021) or to not change in neurological populations and healthy older adults (Yen and Chiu, 2021; Li et al., 2023). In stroke, neurological populations, and healthy older adults, executive functions often show beneficial effects after exergaming (Mura et al., 2018; Gao et al., 2021; Yen and Chiu, 2021; Jiang et al., 2022; Cai et al., 2023), however, systematic reviews showing no beneficial effects exist as well (Soares et al., 2021; Huber et al., 2022a; Li et al., 2023). Moreover, working memory and visuospatial functions have rarely been investigated in exergame studies, and effects were mostly non-significant (Unibaso-Markaida et al., 2019; Cai et al., 2023; Li et al., 2023). To conclude, evidence on the effects of exergames on cognitive functions in chronic stroke and related populations is inconsistent to date, therefore, more research is needed. A possible reason may be varying training protocols, on which we elaborate below.

Another explanatory factor may be different populations, for instance in terms of baseline cognitive status. Beneficial effects of exergames in cognitively impaired populations (Swinnen et al., 2021; Manser and de Bruin, 2024) compared to no beneficial effects in healthy older adults (Sturnieks et al., 2024) may support the hypothesis that cognitively impaired people benefit more from exergame training compared to cognitively healthy people. However, in our sample, baseline cognitive impairment did not affect responding to the intervention (Table 2). This is in line with several meta-analyses, wherein the positive effects of exergames on cognitive functions were mediated by studies including cognitively healthy older adults, while the subgroups of participants with mild cognitive impairment showed no beneficial effects (Soares et al., 2021; Jiang et al., 2022). We also analysed whether number of responders differed based on adherence to the exergame training, or relevant covariates. However, neither of the evaluated parameters showed a significant difference between responders and non-responders (Table 2). Therefore, future research should determine which populations benefit from exergame training, and under which circumstances.

### 4.2 Effects on health-related quality of life

We found a significant interaction effect in the health-related quality of life domain Mobility at follow-up (Figure 4A). Mean changes did not exceed the MCID of 4.5 points for this SIS domain [Supplementary Tables S6, S7 (Lin et al., 2010)], nevertheless the rate of responders was higher in the exergame group compared to the control group (Table 2). This indicates that participants attending the exergame training felt more mobile afterwards, while control participants remarked no difference. Responders in the exergame group showed significantly lower perceived mobility at baseline than non-responders (Table 2), which could indicate that less mobile participants benefitted more from the exergame training. Besides the training, a possible explanation for this finding could be that not only the exergame training but also the study participation itself, including leaving the house and coming into the study centres, may have helped less mobile participants to improve their perceived mobility. However, one must keep in mind that a ceiling effect was probably present in this outcome. The non-responders showed a mean Mobility score of 100 (the maximum) already at baseline. Therefore, the reason for becoming a non-responder could also be that there was no room for improvement over the intervention period. Consequently, it remains open to clarify whether participants with impaired perceived mobility profit more from the exergame training compared to participants with high perceived mobility.

### 4.3 Effects on single-task mobility and indoor gait

We found no interaction effects, but significant within-group improvements in single-task mobility in both groups (Table 3). These results are partly in line with existing literature. Significant within-group improvements in TUG performance after exergaming have been reported in stroke (Unibaso-Markaida and Iraurgi, 2022), and non-significant interaction effects in older adults, MCI and PD patients (Taylor et al., 2018; Elena et al., 2021; Buyle et al., 2022; Cai et al., 2023). However, a larger body of evidence in related populations supports a superior effect of exergames compared to passive and active controls on TUG performance (Fang et al., 2020; Pacheco et al., 2020; Chen et al., 2021; Prosperini et al., 2021; Suleiman-Martos et al., 2021; Zhang et al., 2022; Elhusein et al., 2024; Yoong et al., 2024). Indoor walking did not change in neither group, which is not in line with literature in (chronic) stroke (Corbetta et al., 2015; De Rooij et al., 2016; Gibbons et al., 2016; Ghai et al., 2020; Zhang et al., 2021, 2023; Huber et al., 2022a). Both groups were highly physically active before and throughout the study duration, which may explain their high performance in the gait analyses. For example, our participants showed higher gait speeds (1.24–1.41 m/s) compared to samples of other stroke studies (Yang et al., 2008; Fritz, 2013; Choi et al., 2018; Cikajlo et al., 2020; Lee and Bae, 2022). The high baseline status of the participants may explain the lack of significant interaction effects on single-task motor functions. Another explanation may be a limitation of how the PEMOCS concept was implemented. Namely, many of the more complex and intense motor tasks of the intervention (including squats, double steps, jumps) were added to the games via verbal instruction only. The missing in-game feedback and / or reward on the execution of these complex and intense motor tasks may have induced participants to prioritize the cognitive task (presented and rewarded by the game) over the additional motor task (similarly as suggested in Gallou-Guyot et al., 2023). This led to (partially) incomplete execution of these tasks, which may have diminished possible benefits on motor functions. We present a possible solution for this problem in the Future Directions.

### 4.4 Effects on dual-task mobility and outdoor gait

In dual-task mobility, we found no interaction effects, but significant within-group improvements in both groups. The exergame group showed larger improvements in the cognitive dual-task measures (correct response rates), while the control group exhibited larger improvements in the motor dual-task effect (Table 3). In outdoor gait, gait speed and swing width on the unaffected side showed significant interaction effects favouring the exergame group (Figures 4C,D). The responder analysis in gait speed showed an only slightly higher responder rate in the exergame group compared to the control group (Table 2), and changes did not exceed MCIDs for preferred gait speed [Supplementary Tables S8, S9, (Fulk et al., 2011; Bohannon and Glenney, 2014)]. It should be considered that this responder analysis was performed with a MCID for indoor gait speed, as no value for outdoor gait speed is, to our knowledge, available in literature. Moreover, only the exergame group showed significant improvements in several spatiotemporal gait parameters of the OWA (Table 3). Even though significant, the median changes in these parameters were below the smallest detectable changes (SDCs) determined in a study using the same Physilog sensors in stroke patients (Lefeber et al., 2019), which is why these results warrant cautious interpretation. Nevertheless, the summarized findings imply an overall picture. Namely, these partially beneficial effects on dual-task mobility and outdoor walking may be linked as both tasks can be considered complex walking tasks (Grosboillot et al., 2024). Outdoor walking needs, due to distractions of the environment, more cognitive resources compared to indoor walking (Hillel et al., 2019). The cognitive Timed-up-and-go is even more complex with the additional cognitive task (Grosboillot et al., 2024). Considering that we found significant interaction effects favouring the exergame over the control group in some cognitive measures but not in single motor-tasks, one could hypothesize that the improvements in dual-task mobility and outdoor gait are linked to increased cognitive resources triggered by the exergame intervention. This makes sense when observing that the improvements in dual-task performance in the exergame group were seen in the cognitive dual-task measures, while in the control group they were found in the motor dual-task measures (Table 3). Therefore, exergames may be beneficial for improving activities that require cognitive contribution in chronic stroke survivors.

### 4.5 Influence of training principles

The general training volume of 14 h of this study’s intervention may have been too low to trigger more interaction effects (Saeedi et al., 2021). At least 15 h of training has been recommended for improving cognitive and motor functions in stroke and older adults (Kwakkel et al., 2004; Lauenroth et al., 2016; Laver et al., 2017; Zhang et al., 2021). As duration and session duration of the presented intervention were in line with current recommendations for exergames (Fang et al., 2020; Jiang et al., 2022; Duta et al., 2023; Peng et al., 2024), the frequency may be the most suitable parameter to adjust. This aligns with three systematic reviews showing that frequency was a moderator of exergame-interventions effects (Prosperini et al., 2021; Hai et al., 2022; Jiang et al., 2022). While motor functions profit from high-frequency interventions (Prosperini et al., 2021), a lower frequency (≤ 3x/week) was reported to be more beneficial for improving cognitive functions (Zhu et al., 2016; Gallou-Guyot et al., 2020; Jiang et al., 2022). Three sessions per week may thus be most beneficial for improving both, cognitive and motor functions (Fang et al., 2020; Gallou-Guyot et al., 2020; Prosperini et al., 2021; Hai et al., 2022). Community-dwelling stroke survivors reported in our feasibility study that more than two centre-based sessions were not feasible for them (Huber et al., 2021) (which was the reason for not implementing a higher frequency in this study). Therefore, a blended-therapy approach may be a possible solution to increase the number of sessions per week (Mehra et al., 2018) (see Section 4.8).

A second influential training principle may have been insufficient exercise intensity. It has been postulated that cognitive and motor benefits of exergames (and other motor-cognitive and physical trainings) may be induced by neurotrophin-mediated neuroplasticity (Monteiro-Junior et al., 2016; Levin et al., 2017; Stojan and Voelcker-Rehage, 2019). Special attention has been given to brain-derived neurotrophic factor (BDNF), insulin growth factor-1 (IGF1), and vascular endothelial growth factor (VEGF), which were suggested to collectively mediate exergame-induced neuroplasticity (Yang et al., 2023). Supportive of this hypothesis are several studies, which found that exergaming increased BDNF levels in chronic stroke, older adults and Parkinson’s disease patients (Monteblanco Cavalcante et al., 2021; Huang et al., 2022; Schaeffer et al., 2022), as well as IGF-1 and other neuroplasticity-indicative biomarkers in people with mild cognitive impairment (Nath et al., 2023). Wide results from animal and human studies report that at least moderate exercise intensity is necessary for BDNF increase (Ploughman et al., 2015; Morais et al., 2018; Li et al., 2024). In accordance, low intensity exercise did not induce neuroplasticity in chronic stroke survivors (Murdoch et al., 2016), while exercise at moderate intensity increased neuroplasticity-associated factors and triggered neuroplasticity in stroke (Boyne et al., 2020; Hill et al., 2023). Moreover, moderate-intensity exercise has also been recommended for improving cognitive and physical functioning in stroke and older adults (Dhir et al., 2021; Yang and Wang, 2021; Li et al., 2022; Maeneja et al., 2024). Exergames have been shown to equal physical activity of low to moderate intensity, depending on the content of the games (Peng et al., 2011; Mat Rosly et al., 2017). Considering this, we hypothesize that the intensity of the exergame intervention in this study was too low to trigger neuroplasticity, and consequently effects on cognitive and motor functions were little. We did not specifically measure intensity during our intervention; however, participants reported their motor-cognitive task load via perceived task difficulty and performance. These parameters may align with intensity and showed that participants were under-challenged (Figures 6A,B). This was already the case in the feasibility study preceding this RCT (Huber et al., 2021), and we took actions in the further development of the PEMOCS concept to address this limitation (Huber et al., 2024b). It seems that the adaptions undertaken [namely, coupling motor and cognitive progression, and inclusion of more difficult motor tasks (Huber et al., 2024b)] did not increase intensity and task load sufficiently. One reason for this may be that these adaptions rather increased complexity of the tasks, instead of raising intensity (compare Section 4.3). Recent findings suggest that physical activity decreases with increased cognitive complexity in step-based exergames (Muller et al., 2023). We present a possible solution in Section 4.8. Two further reasons for insufficient intensity may be related to limitations of the used exergaming device (compare Section 4.7, limitations 1 + 2).

### 4.6 Compliance, adherence, and safety

Compliance and adherence to the exergame intervention was high, which is in line with previous studies (Pacheco et al., 2020; Rüth et al., 2023; Tsurayya et al., 2023). The attrition rate was at the upper edge or higher compared to reported ranges (Cheok et al., 2015; Taylor et al., 2018; Yoong et al., 2024), which may be attributed to different reasons in the two treatments groups. In the exergame group, several causally unrelated adverse events occurred, which hindered participants from further study participation. In the control group, some participants reported boredom or even discontent with the study, as they received no intervention and still had to be available for the phone calls and answer the same questions every week. Hence, withdrawals were unrelated to the exergame intervention, however, control groups should be planned differently in future studies. Finally, no causally related adverse events and few technical problems occurred, which is in line with previous exergame studies (Cheok et al., 2015; Pacheco et al., 2020; Prosperini et al., 2021; Rüth et al., 2023; Yoong et al., 2024).

### 4.7 Strengths and limitations

Strengths of the presented study are the following. (1) We implemented a concept-guided exergame intervention with a personalized progression and evidence-based rationales for all exercise variables (Huber et al., 2024b). (2) We used a customized and step-based exergame device with cognitively challenging games to be played in a standing position (Deutsch et al., 2019; Gallou-Guyot et al., 2020; Manser et al., 2024). (3) We assessed cognitive functions, dual-task ability, indoor and outdoor spatiotemporal gait parameters, which are outcomes that are yet under-investigated in exergame studies (Isha, 2019; Gallou-Guyot et al., 2020; Huber et al., 2022a). (4) The assessors in this study completed MoCA training certification before conducting assessments, which ensures high quality and validity of the assessment performance (mocacognition.com). However, there are also several limitations, which should be considered. (1) Several motor-cognitive skill categories of the PEMOCS concept’s underlying taxonomy could not be filled sufficiently due to limitations of the exergame device used. Especially the skill-categories in the higher difficulty levels [see Section 2.4 and (Huber et al., 2024b)] contained only few or even no games, which led to limitations for participants moving in these higher levels. Most importantly, not all cognitive domains could be targeted, and insufficient variability was provided in training sessions for these participants. This may be especially relevant for the presented study population as many participants presented mild residual impairments and may probably have profited from more difficult games and higher variability in the higher levels. (2) Due to a lack of resources for additional developmental work, many of the more complex and intense motor tasks of the intervention were only instructed verbally instead of being integrated into the game, so in-game feedback on their execution was missing. This is unfortunate in the light of literature describing that one advantage of exergames is multisensory feedback and higher enjoyment, which motivates the trainee for more exercise repetitions at a higher intensity (Sailer et al., 2017; Valenzuela et al., 2018). We had started the integration, e.g., a battery visually rewarded participants for dribbling in the according games [(Huber et al., 2024b), Supplementary material S2], however, for future implementations of the PEMOCS concept, in-game feedback for all tasks should be provided. (3) Women were clearly underrepresented in our study, which limits the generalizability of our results to the general chronic stroke population. This is a known phenomenon in studies implementing exercise interventions in stroke (Li et al., 2022), also represented in comparable examples (Hung et al., 2017; Cikajlo et al., 2020; Lee and Bae, 2022; Sultan et al., 2023). A possible reason may be that women are on average older than men are, when they experience a stroke (Eriksson et al., 2021). Consequently, they are more severely impaired and less able to participate in exercise (Eriksson et al., 2021). (4) To calculate the gait variability index (GVI), values for step length and step time had to be estimated from other parameters, as they were not provided by the Gait Up system. For exact procedures, see Supplementary material S1.

### 4.8 Future directions

Appropriate schedules and settings for exergame training to improve cognitive functions, health-related quality of life, indoor and outdoor gait in chronic stroke should be further investigated in future high-quality trials. For such trials as well as an implementation in clinical practice, cost and time efforts of the intervention should be considered. A possible solution to increase the number of sessions per week would be to implement a blended therapy approach, meaning that participants attend one or two supervised sessions per week in a study centre and perform further sessions at home with a telerehabilitation system (Baschung-Pfister et al., 2021; Seinsche et al., 2022). This would combine close contact and supervision by the study / health professionals with reduced time expense and travel-time to the study centres while enabling a higher training frequency (Mehra et al., 2018). Such an intervention design may be more attractive for potential participants with limited time resources or ability to reach the study centres. It may thus help increase the recruitment rate of more severely impaired study participants. To increase the intensity of the exergame training and reduce the prioritization problem (compare Section 4.3), we suggest increasing game speed before progressing game complexity (Muller et al., 2023). Participants would then execute simpler tasks at a higher speed, which may increase the cardiovascular load and cognitive difficulty of the training. Additionally, in-game feedback / reward on all tasks should be included to increase the motivation for proper task execution (compare Section 4.7, limitation 2). This would warrant adjusting the progression rules in the application of the PEMOCS concept (Huber et al., 2024b). Additionally, an objective performance parameter should be included (Huber et al., 2024b), which would enable automated progression and variability independent of a training supervisor. This would contribute to the self-reliant home-based sessions as well as help reducing the time efforts of the personal providing the intervention and therefore enable more patients to receive the intervention. Finally, to contribute to the on-going debate on the role of disease duration in chronic stroke survivors, a future study could investigate the effects of exergame training on cognitive functions in patients 6–24 months post-stroke (Saa et al., 2021). In such a sample earlier post-stroke, the cognitive benefits of exergame training may be greater (Saa et al., 2021).

## 5 Conclusion

Concept-guided, personalized, motor-cognitive exergame training preserved global cognitive functioning in chronic stroke, while it showed no additional benefits compared to usual care only. The rate of responders was higher in the exergame group compared to the control group. Secondarily, significant beneficial effects were found on alertness, working memory, perceived mobility, as well as outdoor gait speed and swing width on the unaffected side. No potential characteristic of responders could be identified as responders and non-responders did not differ in terms of adherence to intervention, baseline value of the according outcome, or age. Therefore, the identification of responsive sub-populations remains subject to future research. Participants showed high adherence to the training and no related adverse events occurred. Reasons for the lack of significant findings may be a high-functioning study population in combination with an insufficient training load in terms of session frequency and intensity. Future studies may increase number of sessions per week by implementing a blended therapy approach, and intensity by adjusting training progression in the PEMOCS concept.

## Data Availability

The raw data supporting the conclusions of this article will be made available by the authors, without undue reservation.

## Footnotes

^1^ mocacognition.com
