# Supplementary material for: PEMOCS: effects of a concept-guided, PErsonalized, MOtor-Cognitive exergame training on cognitive functions and gait in chronic Stroke—a randomized, controlled trial
Source: Front Aging Neurosci. 2025 Mar 13;17:1514594. doi: 10.3389/fnagi.2025.1514594 (PMC11965908; doi:10.3389/fnagi.2025.1514594)
Supplement: Supplementary file 1 [file Data_Sheet_1.docx]

GVI calculations

Following the procedures described in Gouelle et al. 2013^1^

1. **Extract gait parameters provided by Gait Up file**:
   Stride time (s), stance phase (% of cycle duration), swing phase (% of cycle duration),
   double support phase (% of cycle duration), stride length (m), stride velocity (m/s)
2. **Gait parameters needed to calculate the GVI**: provided (P), calculated (C), estimated (E)
   (P) Stride time
   (C) Stance time = (stride time/100) * stance phase
   (C) Swing time = (stride time/100) * swing phase
   (C) DS time = (mean (stride time R/stride time L)/100) * DS phase
   (C) SS time = stance time - DS time
   (E) Step time = swing time + 0.5*DS time
   (P) Stride length (cm)
   (E) Step length = stride length * (Step time/Stride time)
   (P) Stride velocity (cm/s)

   *Calculations (C) and estimations (E) performed in Excel, compare template provided online (https://doi.org/ 10.5281/zenodo.14849242)
3. **Calculations to prepare parameters for step 4**
   1. R Script 10MWT: gvi_10mwt.R
   2. R Code OWA: gvi_owa.R

🡪 Scripts are provided online (https://doi.org/ 10.5281/zenodo.14849242)

1. **GVI calculation by Excel macros file** provided in Gouelle et al., 2013^1^
   🡪 enter parameters from step 3.

^1^Gouelle, A., Mégrot, F., Presedo, A., Husson, I., Yelnik, A., & Penneçot, G. F. (2013). The Gait Variability Index:

A new way to quantify fluctuation magnitude of spatiotemporal parameters during gait. *Gait and Posture*, *38*(3),

461–465. https://doi.org/10.1016/j.gaitpost.2013.01.013
