# Supplementary material for: PEMOCS: effects of a concept-guided, PErsonalized, MOtor-Cognitive exergame training on cognitive functions and gait in chronic Stroke—a randomized, controlled trial
Source: Front Aging Neurosci. 2025 Mar 13;17:1514594. doi: 10.3389/fnagi.2025.1514594 (PMC11965908; doi:10.3389/fnagi.2025.1514594)
Supplement: Supplementary file 2 [file Data_Sheet_2.docx]

Supplement 2: Supplementary Tables

**Table S1: Median values per group of all cognitive outcomes at the three time points**

|  | **Intervention Group** | | | **Control Group** | | |
| --- | --- | --- | --- | --- | --- | --- |
| **Outcome** | **T0** | **T1** | **T2** | **T0** | **T1** | **T2** |
| **MoCA** (N=46) | 25 (23, 27) | 26.5 (25,27) | 25 (24,28) | 26 (24,28) | 26 (22,28) | 24.5 (23,27) |
| **SRT** (N=46) |  |  |  |  |  |  |
| Intrinsic visual, RT [ms] | 247 (208,287) | 239 (211,272) | 227 (204,274) | 223 (209,242) | 229 (209,246) | 237 (216,247) |
| Intrinsic visual, missed | 0 (0,0) | 0 (0,0) | 0 (0,0) | 0 (0,0) | 0 (0,0) | 0 (0,0) |
| Intrinsic visual, mistakes | 1 (0,1) | 1 (0,1) | 1 (0,2) | 1 (0,3) | 0.5 (0,2) | 0.5 (0,3) |
| CM-P visual, RT [ms] | 268 (239,316) | 231 (203,274) | 254 (222,293) | 250 (221,278) | 241 (223,266) | 247 (223,260) |
| CM-P visual, missed | 0 (0,1) | 0 (0,0) | 0 (0,0) | 0 (0,0) | 0 (0,0) | 0 (0,0) |
| CM-P visual, mistakes | 0 (0,0) | 0 (0,0) | 0 (0,0) | 0 (0,0) | 0 (0,0) | 0 (0,0) |
| UM-P visual, RT [ms] | 282 (243,339) | 247 (218,308) | 259 (231,318) | 275 (229,306) | 262 (238,324) | 257 (237,300) |
| UM-P visual, missed | 0 (0,0) | 0 (0,0) | 0 (0,0) | 0 (0,0) | 0 (0,0) | 0 (0,0) |
| UM-P visual, mistakes | 0 (0,1) | 0 (0,0.5) | 0 (0,0) | 0 (0,0) | 0 (0,0) | 0 (0,0) |
| Intrinsic auditory, RT [ms] | 302 (281,366) | 316 (261,371) | 312 (273,358) | 291 (272,335) | 294 (275,327) | 300 (275,318) |
| Intrinsic auditory, missed | 0 (0,0) | 0 (0,0) | 0 (0,0) | 0 (0,0) | 0 (0,0) | 0 (0,0) |
| Intrinsic auditory, mistakes | 1 (1,1) | 1 (0,2) | 1 (1,2) | 0.5 ( 0,2) | 0.5 (0,2) | 1 (0,2) |
| CM-P auditory, RT [ms] | 354 (281,408) | 336 (283,375) | 335 (280,385) | 317 (292,386) | 319 (269,335) | 319 (285,333) |
| CM-P auditory, missed | 0 (0,0) | 0 (0,1) | 0 (0,0) | 0 (0,1) | 0 (0,0) | 0 (0,0) |
| CM-P auditory mistakes | 0 (0,1) | 0 (0,1) | 0 (0,0) | 0 (0,1) | 0 (0,1) | 0 (0,1) |
| UM-P auditory, RT [ms] | 332 (299,380) | 300 (281,356) | 318 (273,372) | 301 (288,378) | 305 (287,335) | 295 (276,326) |
| UM-P auditory, missed | 0 (0,1) | 0 (0,1) | 0 (0,0) | 0 (0,0) | 0 (0,0) | 0 (0,0) |
| UM-P auditory, mistakes | 0 (0,1) | 0 (0,1) | 0 (0,1) | 0 (0,1) | 0 (0,0) | 0 (0,1) |
| **TMT** (N=46) |  |  |  |  |  |  |
| -A, time [s] | 23.79 (17.92,33.7) | 20.14 (16.98,39.32) | 20.35 (15.60,30.54) | 21.67 (17.21,27.81) | 21.13 (16.16,26.30) | 20.31 (17.15,26.96) |
| -A, mistakes | 1 (0,2.5) | 0.5 (0,3) | 0.5 (0,2) | 0 (0,1) | 0 (0,0) | 0 (0,1) |
| -B, time [s] | 40.73 (31.78,68.09) | 43.71 (28.77,61.0) | 42.17 (36.15,51.24) | 40.86 (31.34,51.30) | 42.67 (25.96,59.89) | 40.33 (25.17,64.84) |
| -B, mistakes | 2 (1,5) | 3.5 (1,5) | 2 (1,3.5) | 3 (1,4) | 2.5 (1,6) | 1.5 (0,5.5) |
| B-A ratio | 1.80 (1.52,1.99) | 1.61 (1.34,2.37) | 1.78 (1.50,2.39) | 1.85 (1.62,2.31) | 1.94 (1.37,2.46) | 1.77 (1.30,2.61) |
| **Stroop** (N=45) |  |  |  |  |  |  |
| Reading baseline, RT [s] | 0.87 (0.78,0.92) | 0.86 (0.77,0.94) | 0.87 (0.81,0.94) | 0.88 (0.82,0.94) | 0.89 (0.77,0.96) | 0.92 (0.80,0.95) |
| Reading baseline, mistakes | 0 (0,1) | 0 (0,1) | 0 (0,1) | 0 (0,2) | 0 (0,1) | 0 (0,0) |
| Reading interference, RT [s] | 1.08 (1.01,1.22) | 1.06 (1.01,1.25) | 1.09 (0.95,1.32) | 1.22 (1.06,1.34) | 1.17 (1.01,1.28) | 1.13 (1.07,1.27) |
| Reading interference, mistakes | 1 (0,3) | 1 (0,2) | 1 (0,3.5) | 3 (0,8) | 3 (0,6) | 1.5 (0,5) |
| Reading interference tendency [s] | 0.26 (0.16,0.33) | 0.19 (0.09,0.31) | 0.25 (0.13,0.40) | 0.34 (0.25,0.41) | 0.28 (0.21,0.37) | 0.26 (0.21,0.38) |
| Naming baseline, RT [s] | 0.85,0.74,0.90) | 0.83 (0.76,0.89) | 0 (0,0) | 0.86 (0.79,0.92) | 0.85 (0.75,0.92) | 0.83 (0.77,0.92) |
| Naming baseline, mistakes | 0 (0,0) | 0 (0,1) | 0.85 (0.74,0.91) | 0 (0,0) | 0 (0,1) | 0 (0,0) |
| Naming interference, RT [s] | 1.03 (0.92,1.29) | 1.02 (0.89,1.25) | 0 (0,2) | 1.04 (0.89,1.13) | 1.06 (0.88,1.18) | 1.00 (0.88,1.66) |
| Naming interference, mistakes | 0 (0,2.5) | 1 (0,3) | 1.07 (0.91,1.22) | 1 (0,3.5) | 1 (0,1) | 1 (0,1) |
| Naming interference tendency [s] | 0.18 (0.10,0.41) | 0.19 (0.09,0.31) | 0.21 (0.14,0.31) | 0.16 (0.07,0.32) | 0.16 (0.12,0.25) | 0.16 (0.07,0.39) |
|  |  |  |  |  |  |  |
|  |  |  |  |  |  |  |
|  |  |  |  |  |  |  |
| **NBT** (N=45) |  |  |  |  |  |  |
| RT correct [s] | 0.85 (0.70,1.02) | 0.88 (0.73,1.04) | 0.86 (0.82,1.07) | 0.84 (0.75,0.94) | 0.76 (0.72,0.98) | 0.83 (0.70,0.94) |
| correct | 11 (10,12) | 12 (10,13) | 13 (10,14) | 12 (10,14) | 13 (9,15) | 12.5 (10,14) |
| omissions | 4 (3,5) | 3 (2,4) | 2 (1,5) | 3 (1,5) | 2 (0,5) | 2.5 (1,5) |
| mistakes | 4 (2,7) | 2 (1,4) | 2 (1,4) | 4 (2,9) | 3.5 (1,7) | 2.5 (1,8) |
| **MRT** (N=45) |  |  |  |  |  |  |
| RT correct [s] | 17.20 (14.07,25.01) | 18.19 (12.97,23.28) | 17.30 (14.29,22.19) | 17.15 (14.66,21.84) | 16.51 (13.26,23.26) | 16.98 (13.34,21.89) |
| Accuracy | 0.80 (0.80,0.85) | 0.87 (0.73,0.88) | 0.87 (0.75,0.90) | 0.80 (0.71,0.90) | 0.87 (0.80,0.93) | 0.90 (0.77,0.93) |
| correct | 24 (21, 25.5) | 26 (22,26.5) | 26 (22.5,27) | 24 (21, 27) | 26 (24,28) | 27 (23,28) |

*Values in median (interquartile range). C/UM-P: cross-/uni-modal phasic. MoCA: Montreal Cognitive Assessment. MRT: Mental Rotation Test. NBT: N-back Test. RT: reaction time. SRT: Simple reaction test. Stroop: Stroop Interference Test. TMT: Trail-making Test.*

**Table S2: Median values per group of all health-related quality of life outcomes at the three time points**

|  | **Intervention Group** | | | **Control Group** | | |
| --- | --- | --- | --- | --- | --- | --- |
| **SIS Domain** (N=43) | **T0** | **T1** | **T2** | **T0** | **T1** | **T2** |
| total score | 84.16 (73.57,89.01) | 84.36 (77.14,91.72) | 85.41 (74.76,91.93) | 80.19 (71.8,92.0) | 80.56 (71.44,90.59) | 84.92 (70.51,93.0) |
| Strength | 75.0 (68.75,87.50) | 75.0 (68.75,87.50) | 75.0 (68.75,93.75) | 68.75 (43.75,93.75) | 68.75 (40.63,96.88) | 75.0 (53.13,90.63) |
| Memory | 78.57 (64.28,85.71) | 78.57 (71.43,85.71) | 78.57 (64.29,92.86) | 82.14 (64.29,92.86) | 89.29 (76.79,92.86) | 89.29 (69.64,92.86) |
| Emotions | 83.33 (75.0,91.67) | 83.33 (77.78,88.89) | 83.33 (77.78,88.89) | 88.89 (77.78,94.44) | 86.11 (69.44,90.28) | 86.11 (77.78,88.89) |
| Communication | 82.14 (75.0,85.71) | 85.71 (75.0,96.43) | 89.29 (78.57,96.43) | 85.71 (71.43,96.43) | 89.29 (71.43,94.64) | 89.29 (76.79,94.64) |
| Activity | 95.0 (85.0,100.0) | 100.0 (77.50,100.0) | 97.50 (82.50,100.0) | 95.0 (82.50,100.0) | 92.50 (78.75,100.0) | 95.0 (80.0,100.0) |
| Mobility | 94.44 (86.11,100.0) | 100.0 (88.89,100.0) | 97.22 (94.44,100.0) | 97.22 (94.44,100.0) | 94.44 (84.72,100.0) | 94.44 (86.11,100.0) |
| Hand | 85.0 (50.0,100.0) | 90.0 (50.0,100.0) | 85.0 (60.0,100.0) | 100.0 (55.0,100.0) | 90.0 (27.50,100.0) | 95.0 (47.50,100.0) |
| Participation | 87.50 (68.75,100.0) | 96.88 (75,96.88) | 90.63 (75.0,100.0) | 78.13 (62.50,100.0) | 81.25 (65.63,96.88) | 90.63 (73.44,98.44) |
| Recovery | 85.0 (70.0,91.0) | 90.0 (70.0,95.0) | 85.0 (70.0,95.0) | 80.0 (70.0,92.0) | 75.50 (67.75,90.50) | 89.50 (73.75,91.75) |

*Values in median (interquartile range). SIS: Stroke Impact Scale 3.0.*

**Table S3: Median values per group of all motor outcomes at the three time points**

|  | **Intervention Group** | | | **Control Group** | | |
| --- | --- | --- | --- | --- | --- | --- |
| **Outcome** | **T0** | **T1** | **T2** | **T0** | **T1** | **T2** |
| **TUG** (N=46, TUG-Cogn N=45) |  |  |  |  |  |  |
| single task, time [s] | 6.33 (5.33,8.42) | 6.33 (5.0,8.08) | 6.33 (5.50,7.83) | 7.0 (5.42,9.33) | 6.67 (5.0,8.67) | 7.0 (5.08,8.92) |
| cogn-dual task, time [s] | 8.0 (6.17,11.17) | 7.0 (5.50,9.50) | 7.0 (5.25,10.25) | 11.0 (7.50,13.25) | 9.33 (6.33,12.33) | 9.50 (6.67,11.25) |
| motor dual task effect [%]° | 20.83 (9.33,39.38) | 10.32 (6.36,33.75) | 9.72 (0.0,24.64) | 37.64 (25.97,55.28) | 27.31 (20.0,42.86) | 24.73 (15.97,35.12) |
| CRR single-task | 0.68 (0.46,0.82) | 0.77 (0.49,0.88) | 0.80 (0.42,0.95) | 0.56 (0.43,0.62) | 0.59 (0.80,0.71) | 0.60 (0.50,0.74) |
| CRR dual-task | 0.55 (0.42,0.69) | 0.59 (0.42,0.80) | 0.64 (0.40,0.87) | 0.51 (0.39,0.62) | 0.49 (0.43,0.64) | 0.54 (0.41,0.66) |
| **10MWT** (N=46) |  |  |  |  |  |  |
| time [s] | 7.67 (7.0,9.0) | 7.67 (6.92,8.75) | 7.67 (7.0,9.0) | 8.17 (7.0,9.33) | 8.0 (6.67,9.33) | 7.83 (7.08,8.92) |
| speed [m/s] | 1.38 (1.14,1.44) | 1.36 (1.19,1.53) | 1.41 (1.15,1.48) | 1.25 (1.07,1.43) | 1.24 (1.10,1.42) | 1.34 (1.15,1.46) |
| cadence [steps/min] | 110.20 (104.85,114.94) | 111.04 (109.35,114.87) | 110.16 (107.35,115.02) | 107.67 (100.68,112.09) | 110.66 (99.87,112.31) | 111.66 (103.78,113.86) |
| stride length affected [m] | 1.46 (1.21,1.54) | 1.45 (1.27,1.58) | 1.48 (1.26,1.59) | 1.38 (1.26,1.51) | 1.39 (1.24,1.52) | 1.38 (1.27,1.55) |
| stride length unaffected [m] | 1.47 (1.23,1.57) | 1.43 (1.27,1.61) | 1.47 (1.26,1.59) | 1.39 (1.27,1.49) | 1.39 (1.29,1.50) | 1.41 (1.26,1.53) |
| stride time affected [s] | 1.09 (1.04,1.15) | 1.08 (1.04,1.12) | 1.09 (1.04,1.12) | 1.11 (1.06,1.20) | 1.08 (1.05,1.21) | 1.08 (1.05,1.19) |
| stride time unaffected [s] | 1.09 (1.05,1.15) | 1.08 (1.05,1.12) | 1.09 (1.04,1.13) | 1.16 (1.07,1.22) | 1.09 (1.07,1.21) | 1.08 (1.06,1.19) |
| double support time [*] | 21.86 (20.53,23.86) | 21.86 (20.24,24.28) | 21.85 (19.96,24.57) | 22.93 (20.76,25.65) | 21.57 (20.30,24.46) | 21.88 (20.47,24.84) |
| stance time affected [*] | 60.76 (60.21,62.02) | 60.89 (59.84,62.92) | 61.41 (59.32,62.48) | 59.84 (59.03,62.66) | 60.18 (59.52,61.52) | 60.27 (59.31,62.03) |
| stance time unaffected [*] | 61.24 (60.37,62.82) | 60.80 (60.26,62.41) | 60.97 (60.23,62.57) | 61.85 (61.01,64.87) | 61.71 (60.08,64.49) | 61.27 (60.16,63.77) |
| swing time affected [*] | 39.24 (37.98,39.79) | 39.11 (37.08,40.16) | 38.59 (37.52,40.68) | 40.16 (37.34,40.97) | 39.82 (38.48,40.48) | 39.73 (37.97,40.69) |
| swing time unaffected [*] | 38.76 (37.18,39.63) | 39.20 (37.59,39.74) | 39.03 (37.44,39.77) | 38.15 (35.13,39.11) | 38.29 (35.51,39.92) | 38.74 (36.23,39.84) |
| swing width affected [cm] | 4.88 (3.30,6.07) | 4.97 (3.18,6.39) | 4.37 (3.0,5.65) | 4.58 (3.49,6.18) | 4.60 (3.67,6.33) | 4.67 (3.23,7.31) |
| swing width unaffected [cm] | 4.52 (3.79,5.91) | 4.73 (4.04,6.11) | 4.83 (4.02,7.05) | 3.45 (2.65,4.90) | 3.40 (2.37,4.70) | 3.45 (2.25,5.22) |
| asymmetry | 2.95 (1.37,5.82) | 2.57 (1.39,5.0) | 3.87 (1.98,5.70) | 4.02 (2.38,15.09) | 5.10 (1.39,14.63) | 3.48 (1.42,13.43) |
| GVI affected | 86.97 (77.64,95.19) | 86.95 (81.11,104.66) | 87.21 (75.88,94.80) | 80.76 (71.76,87.41) | 83.80 (74.36,91.20) | 79.26 (72.14,86.61) |
| GVI unaffected | 84.64 (76.73,95.84) | 89.05 (80.62,97.39) | 82.69 (77.59,87.32) | 81.76 (72.83,89.29) | 82.92 (75.73,90.89) | 81.80 (76.01,91.39) |
| walk ratio [cm/steps/min] | 0.66 (0.57,0.71) | 0.66 (0.58,0.72) | 0.66 (0.59,0.71) | 0.66 (0.61,0.68) | 0.65 (0.59,0.68) | 0.63 (0.59,0.68) |
| **10MWT fast**, time [s] (N=43) | 4.67 (3.08,6.58) | 4.33 (3.0,5.83) | 4.0 (3.0,5.0) | 4.33 (3.0,6.0) | 4.33 (2.67,7.33) | 5.0 (3.0,6.0) |
| **OWA** (N=43) |  |  |  |  |  |  |
| speed [m/s] | 1.29 (1.15,1.40) | 1.35 (1.26,1.46) | 1.33 (1.24,1.45) | 1.33 (1.11,1.52) | 1.27 (1.17,1.46) | 1.34 (1.24,1.51) |
| cadence [steps/min] | 108.66 (104.43,112.41) | 111.12 (108.43,114.22) | 110.80 (105.54,114.21) | 110.81 (104.59,114.07) | 110.99 (102.72,112.71) | 111.31 (108.94,113.15) |
| stride length affected [m] | 1.43 (1.26,1.49) | 1.44 (1.30,1.52) | 1.42 (1.26,1.55) | 1.41 (1.26,1.59) | 1.37 (1.30,1.57) | 1.40 (1.28,1.58) |
| stride length unaffected [m] | 1.41 (1.26,1.52) | 1.44 (1.30,1.55) | 1.43 (1.28,1.56) | 1.41 (1.28,1.58) | 1.40 (1.29,1.52) | 1.44 (1.29,1.56) |
| stride time affected [s] | 1.11 (1.07,1.15) | 1.08 (1.06,1.11) | 1.08 (1.05,1.14) | 1.10 (1.05,1.15) | 1.08 (1.07,1.17) | 1.08 (1.06,1.10) |
| stride time unaffected [s] | 1.11 (1.07,1.15) | 1.08 (1.06,1.11) | 1.08 (1.05,1.14) | 1.09 (1.05,1.15) | 1.08 (1.07,1.17) | 1.08 (1.06,1.10) |
| double support time [*] | 22.58 (20.47,26.59) | 21.80 (20.04,25.49) | 22.85 (16.34,64.59) | 21.98 (20.23,24.59) | 22.65 (19.83,24.26) | 22.13 (14.87,47.69) |
| stance time affected [*] | 61.49 (59.81,63.36) | 61.47 (59.80,62.39) | 61.64 (59.94,62.77) | 60.39 (59.39,62.57) | 59.53 (59.05,61.80) | 60.05 (59.22,61.76) |
| stance time unaffected [*] | 61.52 (60.50,63.06) | 60.98 (59.84,62.91) | 61.02 (60.47,62.99) | 61.17 (60.29,64.60) | 61.22 (60.40,65.27) | 61.18 (60.21,63.67) |
| swing time affected [*] | 38.52 (36.64,40.19) | 38.54 (37.61,40.20) | 38.37 (37.23,40.60) | 39.61 (37.43,40.61) | 40.48 (38.20,40.95) | 39.95 (38.24,40.78) |
| swing time unaffected [*] | 38.49 (36.94,39.50) | 39.03 (37.09,40.16) | 39.98 (37.01,39.53) | 38.83 (35.40,39.71) | 38.79 (34.74,39.60) | 38.82 (36.33,39.79) |
| swing width affected [cm] | 5.20 (2.98,6.55) | 4.90 (3.23,6.10) | 4.65 (4.05,6.63) | 5.0 (4.40,6.20) | 5.05 (4.13,6.28) | 4.80 (3.50,6.20) |
| swing width unaffected [cm] | 4.55 (3.80,6.13) | 5.30 (4.13,6.35) | 5.20 (4.35,6.80) | 3.80 (2.60,6.0) | 3.70 (2.65,5.55) | 4.0 (3.20,5.70) |
| asymmetry | 2.95 (2.08,4.35) | 3.85 (2.40,5.84) | 3.10 (2.12,4.23) | 3.80 (2.70,12.20) | 4.55 (3.40,18.40) | 5.20 (2.70,13.0) |
| GVI affected | 79.45 (73.69,87.24) | 81.48 (77.63,84.0) | 82.61 (73.60,86.66) | 79.73 (73.06,85.45) | 74.69 (71.48,83.24) | 77.42 (71.14,86.43) |
| GVI unaffected | 77.30 (73.10,86.56 | 80.49 (77.67,85.22) | 81.48 (76.56,85.27) | 83.26 (72.19,86.74) | 81.38 (75.76,84.85) | 83.58 (76.14,90.01) |
| walk ratio [cm/steps/min] | 0.66 (0.57,0.70) | 0.64 (0.59,0.69) | 0.65 (0.59,0.70) | 0.66 (0.63,0.69) | 0.65 (0.63,0.68) | 0.66 (0.62,0.68) |

*Values in median (interquartile range). *expressed in % of the whole gait cycle. CRR: correct response rate. GVI: gait variability index. OWA: Outdoor walking assessment. TUG: Timed-up-and-go test. 10MWT: 10-meter walk test.*

**Table S4: Intention-to-treat Analyses of Cognitive Outcomes**

| **Outcome** | **Period** | **Δ Intervention** | **Δ Control** | **Covariates** | **violated Assumptions** | **b (SE)** | **p** | **ES** |
| --- | --- | --- | --- | --- | --- | --- | --- | --- |
| **MoCA** (N=46) | T0🡪T1 | 0 (0,2.0) | -0.5 (-2.0,1.0) | none | normality of RE | -1.27 (0.78) | 0.11 | 0.17 |
|  | T0🡪T2 | 0 (-1.0,1.0) | 0 (-2.0,1.0) |  |  | -0.72 (0.78) | 0.36 | 0.10 |
| **SRT** (N=46) |  |  |  |  |  |  |  |  |
| Intrinsic visual, RT [ms] | T0🡪T1 | 0 (-16,10) | 1 (-13,30) | none | homogeneity | 14.34 (9.41) | 0.13 | 0.16 |
|  | T0🡪T2 | -2 (-23,5)^M^ | 3 (-16,23) |  |  | 23.17 (9.34) | **0.02*** | 0.26 |
| Intrinsic visual, missed | T0🡪T1 | 0 (0,0) | 0 (0,0) | none | - | -0.01 (<0.01) | > 0.99 | - |
|  | T0🡪T2 | 0 (0,0) | 0 (0,0) |  |  | 0.31 (<0.01) | > 0.99 |  |
| Intrinsic visual, mistakes | T0🡪T1 | 0 (-1,1) | 0 (-1,1) | age* |  | 0.40 (0.37) | 0.29 |  |
|  | T0🡪T2 | 0 (0,2) | 0 (-1,0) |  |  | 0.22 (0.34) | 0.51 |  |
| CM-P visual, RT [ms] | T0🡪T1 | -8 (-53,0)*^,M^ | 1 (-29,11) | age | homogeneity | 14.12 (12.58) | 0.26 | 0.12 |
|  | T0🡪T2 | 0 (-41,11) | -10 (-31,21) |  |  | 8.28 (12.48) | 0.51 | 0.07 |
| CM-P visual, missed° | T0🡪T1 | 0 (-1,0)^M^ | 0 (0,0) | education* | - | 1.61 (1.36) | 0.24 | - |
|  | T0🡪T2 | 0 (0,0) | 0 (0,0) |  |  | 1.39 (1.34) | 0.30 |  |
| CM-P visual, mistakes | T0🡪T1 | 0 (0,0) | 0 (0,0) | none |  | 2.05 (0.64) | 0.001* |  |
|  | T0🡪T2 | 0 (0,0) | 0 (0,0) |  |  | 2.24 (0.67) | 0.001* |  |
| UM-P visual, RT [ms] | T0🡪T1 | -19 (-36,0)*^,M^ | -4 (-35,25) | none | none | 8.93 (13.45) | 0.51 | 0.07 |
|  | T0🡪T2 | -3 (-55,27) | -8 (-50,24)^M^ |  |  | -4.61 (13.35) | 0.73 | 0.04 |
| UM-P visual, missed | T0🡪T1 | 0 (0,0) | 0 (0,0) | none | - | 15.39 (2386.16) | 0.99 | - |
|  | T0🡪T2 | 0 (0,0) | 0 (0,0) |  |  | 16.65 (2386.16) | 0.99 |  |
| UM-P visual, mistakes° | T0🡪T1 | 0 (-0.5,0) | 0 (0,0)^M^ | none |  | 1.34 (0.93) | 0.15 |  |
|  | T0🡪T2 | 0 (-1,0) | 0 (0,0) |  |  | 2.08 (0.94) | **0.03*** |  |
| Intrinsic auditory, RT [ms] | T0🡪T1 | 0 (-33,20) | 2 (-26,32) | education | homogeneity | 7.29 (15.52) | 0.64 | 0.05 |
|  | T0🡪T2 | 1 (-25,28) | 5 (-10,22) |  |  | -5.25 (15.41) | 0.73 | 0.04 |
| Intrinsic auditory, missed° | T0🡪T1 | 0 (0,0) | 0 (0,0) | none | - | -0.19 (<0.01) | > 0.99 | - |
|  | T0🡪T2 | 0 (0,0) | 0 (0,0) |  |  | -0.76 (<0.01) | > 0.99 |  |
| Intrinsic auditory, mistakes | T0🡪T1 | 0 (-1, 0.5) | 0 (-1,1) | age* |  | -0.10 (0.36) | 0.79 |  |
|  | T0🡪T2 | 0 (0,1) | 0 (-1,1) |  |  | 0.06 (0.35) | 0.88 |  |
| CM-P auditory, RT [ms] | T0🡪T1 | 0 (-48,18) | -17 (-52,16)*^,M^ | none | homogeneity | -3.51 (15.80) | 0.82 | 0.02 |
|  | T0🡪T2 | 0 (-44,15) | -11 (-65,9)^M^ |  |  | -6.15 (15.69) | 0.70 | 0.04 |
| CM-P auditory, missed | T0🡪T1 | 0 (0,0) | 0 (0,0) | age* | - | -1.11 (0.79) | 0.16 | - |
|  | T0🡪T2 | 0 (0,0) | 0 (0,0) |  |  | -0.54 ( 0.78) | 0.49 |  |
| CM-P auditory, mistakes | T0🡪T1 | 0 (0,0) | 0 (0,1) | age* |  | 0.66 (0.51) | 0.20 |  |
|  | T0🡪T2 | 0 (-1,0) | 0 (0,0) |  |  | 0.85 (0.54 | 0.16 |  |
| UM-P auditory, RT [ms] | T0🡪T1 | -1 (-24,11) | -16 (-47,18) | none | homogeneity | -2.43 (15.22) | 0.87 | 0.02 |
|  | T0🡪T2 | -10 (-61,15) | -16 (-70,-4)*^,M^ |  |  | -9.24 (15.10) | 0.54 | 0.07 |
| UM-P auditory, missed | T0🡪T1 | 0 (0,0) | 0 (0,0) | age | - | -1.57 (1.22) | 0.20 | - |
|  | T0🡪T2 | 0 (-1,0)*^,M^ | 0 (0,0) |  |  | 0.00 (1.37) | > 0.99 |  |
| UM-P auditory, mistakes | T0🡪T1 | 0 (0,0.5) | 0 (0,0) | age* |  | -0.57 (0.63) | 0.36 |  |
|  | T0🡪T2 | 0 (0,0) | 0 (0,1) |  |  | -0.63 (0.55) | 0.26 |  |
| **TMT** (N=46) |  |  |  |  |  |  |  |  |
| -A, time [s] | T0🡪T1 | 0 (-8.88,2.06) | -1.19 (-5.03,1.57) | age*, education | homogeneity, linearity, normality of RE | -0.15 (3.40) | 0.97 | < 0.01 |
|  | T0🡪T2 | -1.06 (-6.53,0.53) | -1.32 (-3.66,0.84) |  |  | 0.16 (3.40) | 0.96 | < 0.01 |
| -A, mistakes° | T0🡪T1 | 0 (-1,0) | 0 (0,0) | none | - | 0.15 (0.36) | 0.68 | - |
|  | T0🡪T2 | 0 (-0.5,1) | 0 (0,0) |  |  | 0.12 (0.38) | 0.76 |  |
| -B, time [s] | T0🡪T1 | -1.23 (-9.77,4.83) | -0.08 (-11.76,4.75) | age* | linearity, normality of  residuals & RE | 1.88 (7.44) | 0.80 | 0.03 |
|  | T0🡪T2 | 0 (-14.75,8.95) | -5.15 (-11.65,3.66) |  |  | 1.13 (7.44) | 0.88 | 0.02 |
| -B, mistakes | T0🡪T1 | 0 (-3, 3.5) | 0 (-3,4) | age* | - | 0.37 (0.21) | 0.08 | - |
|  | T0🡪T2 | 0 (-2,1) | -1 (-3,2.5) |  |  | 0.33 (0.22) | 0.14 |  |
| B-A ratio | T0🡪T1 | 0 (-0.25,0.31) | 0.05 (-0.38,0.53) | none | linearity | 0.05 (0.32) | 0.89 | 0.02 |
|  | T0🡪T2 | 0 (-0.19,0.35) | -0.15 (-0.56,0.59) |  |  | 0.11 (0.32) | 0.72 | 0.04 |
| **Stroop** (N=45) |  |  |  |  |  |  |  |  |
| Reading baseline, RT [s] | T0🡪T1 | 0 (-0.02,0.03) | 0 (-0.05,0.03) | age* | none | 0.01 (0.02) | 0.72 | 0.04 |
|  | T0🡪T2 | 0 (0,0.03)*^,M^ | 0 (-0.02,0.03) |  |  | -0.01 (0.02) | 0.57 | 0.06 |
| Reading baseline, mistakes | T0🡪T1 | 0 (0,0) | 0 (-1,0) | none | - | -0.73 (0.52) | 0.16 | - |
|  | T0🡪T2 | 0 (0,1) | 0 (-1,0) |  |  | -0.75 (0.51) | 0.14 |  |
| Reading interference, RT [s] | T0🡪T1 | 0 (-0.08,0.01) | -0.06 (-0.11,0)*^,M^ | none | none | -0.03 (0.03) | 0.43 | 0.09 |
|  | T0🡪T2 | 0 (-0.05,0.08) | -0.05 (-0.11,0.02)^M^ |  |  | -0.06 (0.03) | 0.06 | 0.20 |
| Reading interference, mistakes | T0🡪T1 | 0 (-1,0) | 0 (-2,0)^M^ | none | - | -0.03 (0.00) | **< 0.001*** | - |
|  | T0🡪T2 | 0 (0,1) | -0.5 (-4,0)^M^ |  |  | -0.48 (0.00) | **< 0.001*** |  |
| R. interference tendency [s] | T0🡪T1 | 0 (-0.07,0.01) | -0.03 (-0.11,0.02)^M^ | none | linearity, normality of RE | -0.03 (0.03) | 0.36 | 0.10 |
|  | T0🡪T2 | 0 (-0.03,0.06) | -0.04 (-0.11,0.02) |  |  | -0.05 (0.03) | 0.14 | 0.16 |
| Naming baseline, RT [s] | T0🡪T1 | 0 (-0.05,0.02) | -0.01 (-0.03,0.02) | age*, education | normality of residuals & RE | 0.003 (0.02) | 0.85 | 0.02 |
|  | T0🡪T2 | 0 (-0.02,0.04) | -0.02 (-0.05,0.02)^M^ |  |  | -0.02 (0.02) | 0.33 | 0.10 |
| Naming baseline, mistakes | T0🡪T1 | 0 (0,0.5) | 0 (0,0) | none | - | -0.22 (0.64) | 0.73 | - |
|  | T0🡪T2 | 0 (0,0) | 0 (0,0) |  |  | -0.17 (0.66) | 0.80 |  |
| Naming interference, RT [s] | T0🡪T1 | 0 (-0.04,0.04) | 0.01 (-0.10,0.05) | age* | linearity, normality of residuals | -0.05 (0.07) | 0.46 | 0.08 |
|  | T0🡪T2 | 0 (-0.04,0.07) | -0.01 (-0.05,0.02) |  |  | -0.05 (0.07) | 0.48 | 0.08 |
| Naming interference, mistakes | T0🡪T1 | 0 (0,1) | 0 (-1,1) | age* | - | -0.69 (0.00) | **< 0.001*** | - |
|  | T0🡪T2 | 0 (-0.5,0.5) | 0 (-1,1) |  |  | -1.45 (0.00) | **< 0.001*** |  |
| N. interference tendency [s] | T0🡪T1 | 0 (-0.05,0.06) | 0.03 (-0.05,0.08) | age* | linearity, normality of residuals | -0.05 (0.07) | 0.44 | 0.08 |
|  | T0🡪T2 | 0.01 (-0.03,0.05) | 0.01 (-0.03,0.04) |  |  | -0.03 (0.07) | 0.67 | 0.05 |
| **NBT** (N=45) |  |  |  |  |  |  |  |  |
| RT correct [s] | T0🡪T1 | 0.01 (-0.07,0.09) | -0.01 (-0.11,0.05) | age* | linearity, normality of  residuals & RE | -0.08 (0.08) | 0.33 | 0.11 |
|  | T0🡪T2 | 0 (-0.04,0.18) | -0.08 (-0.15,0.04)^M^ |  |  | -0.09 (0.08) | 0.25 | 0.13 |
| correct | T0🡪T1 | 1 (0,1) | 0 (-2,1) | age* | - | -0.02 (0.13) | 0.86 | - |
|  | T0🡪T2 | 1 (-0.5,2) | 0 (-1,2) |  |  | -0.02 (0.13) | 0.90 |  |
| omissions | T0🡪T1 | -1 (-2,0) | 0 (-1,1) | age* | - | 0.07 (0.24) | 0.77 | - |
|  | T0🡪T2 | -1 (-2, 0.5) | 0 (-2,1) |  |  | -0.02 (0.24) | 0.95 |  |
| mistakes | T0🡪T1 | -1 (-3,0)*^,M^ | -1 (-3,0)^M^ | age* | - | 0.20 (0.20) | 0.31 | - |
|  | T0🡪T2 | -1 (-2,0)*^,M^ | -1 (-2,0)^M^ |  |  | 0.24 (0.21) | 0.25 |  |
| **MRT** (N=45) |  |  |  |  |  |  |  |  |
| RT correct [s] | T0🡪T1 | -0.74 (-4.08,0.89) | -0.53 (-4.06,2.44) | age* | normality of RE | -0.21 (1.28) | 0.87 | 0.02 |
|  | T0🡪T2 | 0 (-1.68,1.78) | -1.50 (-3.31,1.88) |  |  | -0.62 (1.28) | 0.63 | 0.05 |
| correct | T0🡪T1 | 1 (0,3)*^,L^ | 1 (-1,3)^M^ | education* | - | -0.01 (0.09) | 0.92 | - |
|  | T0🡪T2 | 1 (0,2.5)*^,L^ | 1 (0,3)^M^ |  |  | 0.01 (0.09) | 0.92 |  |
| accuracy | T0🡪T1 | 0.03 (0,0.10)*^,L^ | 0.03 (-0.02,0.10)^M^ | education* | normality of residuals & RE | -0.004 (0.02) | 0.86 | 0.02 |
|  | T0🡪T2 | 0.03 (0,0.08)*^,L^ | 0.03 (0,0.10)*^,M^ |  |  | -0.004 (0.02) | 0.86 | 0.02 |

***Bold*** *& *: significant result. ^M^ / ^L^ : medium / large effect size. ES: effect size, Bravais-Pearson correlation coefficients (r). CM-P: cross-modal phasic. MoCA: Montreal Cognitive Assessment. MRT: Mental Rotation Test. NBT: N-back Test. RE: random effects. RT: reaction time. Stroop: Stroop Interference Test. TMT: Trail-making Test. UM-P: uni-modal phasic.*

**Table S5: Per-protocol Analyses of Cognitive Outcomes**

| **Outcome** | **Period** | **Δ Intervention** | **Δ Control** | **Covariates** | **violated Assumptions** | **b (SE)** | **p** | **ES** |
| --- | --- | --- | --- | --- | --- | --- | --- | --- |
| **MoCA** (N=36) | T0🡪T1 | 1 (-0.5,2.5) | -1 (-2,1) | none | multicollinearity,  normality of REs | -1.72 (0.97) | 0.08 | 0.21 |
|  | T0🡪T2 | 0 (-2.5,3) | 0 (-2,1) |  |  | -0.75 (0.97) | 0.44 | 0.09 |
| **SRT** (N=36) |  |  |  |  |  |  |  |  |
| Intrinsic visual, RT [ms] | T0🡪T1 | -4 (-19,10) | 1 (-14,31) | none | homogeneity, multicollinearity | 12.49 (10.13) | 0.22 | 0.15 |
|  | T0🡪T2 | -7 (-28,7)^M^ | 4 (-19,23) |  |  | 21.74 (10.13) | **0.04*** | 0.25 |
| Intrinsic visual, missed | T0🡪T1 | 0 (0,0) | 0 (0,0) | none | - | -0.001 (<0.01) | > 0.99 | - |
|  | T0🡪T2 | 0 (0,0) | 0 (0,0) |  |  | 0.38 (<0.01) | > 0.99 |  |
| Intrinsic visual, mistakes | T0🡪T1 | 0 (-2.5,1)^M^ | 0 (-1,1) | age | - | 0.56 (0.41) | 0.17 | - |
|  | T0🡪T2 | 0 (-2,1.5) | 0 (-1,0) |  |  | 0.47 (0.37) | 0.21 |  |
| CM-P visual, RT [ms] | T0🡪T1 | -37 (-58,-8)*^,L^ | 2 (-29,12) | none | multicollinearity | 24.17 (15.21) | 0.12 | 0.19 |
|  | T0🡪T2 | -15 (-58,14)^M^ | -12 (-31,23) |  |  | 18.76 (15.21) | 0.22 | 0.15 |
| CM-P visual, missed° | T0🡪T1 | 0 (-1,0)^M^ | 0 (0,0) | none | - | 19.25 (836.10) | 0.98 | - |
|  | T0🡪T2 | 0 (-1,0)*^,L^ | 0 (0,0) |  |  | 19.25 (836.10) | 0.98 |  |
| CM-P visual, mistakes | T0🡪T1 | 0 (0,0) | 0 (0,0) | age | - | 2.45 (0.75) | < 0.01* | - |
|  | T0🡪T2 | 0 (0,0) | 0 (0,0) |  |  | 2.74 (0.80) | < 0.01* |  |
| UM-P visual, RT [ms] | T0🡪T1 | -24 (-38,-11)*^,L^ | -4 (-38,26) | none | multicollinearity,  normality of REs | 18.15 (15.53) | 0.24 | 0.14 |
|  | T0🡪T2 | -53 (-70,11)*^,L^ | -14 (-53,24)^M^ |  |  | 14.45 (15.53) | 0.36 | 0.11 |
|  |  |  |  |  |  |  |  |  |
| UM-P visual, missed° | T0🡪T1 | 0 (-0.5,0) | 0 (0,0) | none | - | 10.30 (144.46) | 0.94 | - |
|  | T0🡪T2 | 0 (-0.5,0)^M^ | 0 (0,0) |  |  | 11.40 (144.46) | 0.94 |  |
| UM-P visual, mistakes° | T0🡪T1 | 0 (-1,0) | 0 (0,0)^M^ | none | - | 1.42 (0.94) | 0.13 | - |
|  | T0🡪T2 | 0 (-1,0.5)^M^ | 0 (0,0) |  |  | 2.17 (0.96) | **0.02*** |  |
| Intrinsic auditory, RT [ms] | T0🡪T1 | -6 (-50,30) | 3 (-31,34) | none | homogeneity, multicollinearity, normality of REs | 10.63 (15.32) | 0.49 | 0.08 |
|  | T0🡪T2 | 8 (-36,26) | 7 (-12,23) |  |  | 12.08 (15.32) | 0.43 | 0.10 |
| Intrinsic auditory, missed | T0🡪T1 | 0 (0,0) | 0 (0,0) | none | - | -0.49 (<0.01) | > 0.99 | - |
|  | T0🡪T2 | 0 (0,0) | 0 (0,0) |  |  | -0.30 (<0.01) | > 0.99 |  |
| Intrinsic auditory, mistakes | T0🡪T1 | 0 (-1.5, 1) | 0 (-1,1) | none | - | -0.07 (0.39) | 0.82 | - |
|  | T0🡪T2 | 0 (-0.5,1) | 0 (-1,1) |  |  | 0.07 (0.38) | 0.85 |  |
| CM-P auditory, RT [ms] | T0🡪T1 | -20 (-55,25) | -21 (-52,16)*^,M^ | none | homogeneity, multicol-linearity, normality of REs | 3.26 (17.32) | 0.85 | 0.02 |
|  | T0🡪T2 | -39 (-84,11)^M^ | -11 (-75,9)^M^ |  |  | 10.47 (17.32) | 0.56 | 0.07 |
| CM-P auditory, missed | T0🡪T1 | 0 (0,0) | 0 (0,0) | age | - | -0.73 (0.85) | 0.39 | - |
|  | T0🡪T2 | 0 (0,0)^M^ | 0 (0,0) |  |  | 0.41 (0.98) | 0.68 |  |
| CM-P auditory, mistakes | T0🡪T1 | 0 (-0.5,0.5) | 0 (0,1) | none | - | 0.77 (0.57) | 0.18 | - |
|  | T0🡪T2 | 0 (-1,0.5) | 0 (0,0) |  |  | 0.88 (0.60) | 0.15 |  |
| UM-P auditory, RT [ms] | T0🡪T1 | -17 (-42,11)^M^ | -16 (-51,24) | none | multicollinearity | 6.36 (17.67) | 0.72 | 0.04 |
|  | T0🡪T2 | -23 (-81,3)*^,L^ | -16 (-73,-8)*^,M^ |  |  | 8.08 (17.64) | 0.65 | 0.06 |
| UM-P auditory, missed | T0🡪T1 | 0 (0,0) | 0 (0,0) | none | - | -1.72 (1.42) | 0.22 | - |
|  | T0🡪T2 | 0 (-1,0)*^,L^ | 0 (0,0) |  |  | 18.43 (9642.41) | > 0.99 |  |
| UM-P auditory, mistakes | T0🡪T1 | 0 (-1,1) | 0 (0,0) | none | - | -0.58 (0.70) | 0.41 | - |
|  | T0🡪T2 | 0 (0,1)^M^ | 0 (0,1) |  |  | -0.82 (0.59) | 0.17 |  |
| **TMT** (N=36) |  |  |  |  |  |  |  |  |
| -A, time [s] | T0🡪T1 | 0.21 (-9.26,11.88) | -1.42 (-5.17,1.75) | age | homogeneity, multicollinearity, linearity, normality of REs | -1.11 (3.46) | 0.75 | 0.04 |
|  | T0🡪T2 | -3.84 (-9.98,0.69)^M^ | -1.36 (-3.92,0.96) |  |  | 3.81 (3.46) | 0.28 | 0.13 |
| -A, mistakes° | T0🡪T1 | 0 (-2.5,1.5) | 0 (0,0) | none | - | 0.10 (0.45) | 0.83 | - |
|  | T0🡪T2 | 0.5 (-1,2) | 0 (0,0) |  |  | -0.06 (0.51) | 0.91 |  |
| -B, time [s] | T0🡪T1 | -4.07 (-10.55,10.88) | -0.16 (-13.49,5.72) | age* | homogeneity, multicollinearity, linearity, normality of res. & REs | 3.62 (8.30) | 0.66 | 0.05 |
|  | T0🡪T2 | -7.58 (-18.64,9.01) | -5.51 (-11.78,4.88) |  |  | 7.38 (8.30) | 0.38 | 0.11 |
| -B, mistakes | T0🡪T1 | -2 (-5.5, 4) | 0 (-3,4) | none | - | 0.58 (0.23) | **0.01*** | - |
|  | T0🡪T2 | -0.5 (-5,2) | -1 (-3,3) |  |  | 0.55 (0.24) | **0.02*** |  |
| B-A ratio | T0🡪T1 | -0.04 (-0.34,0.28) | 0.08 (-0.38,0.56) | none | homogeneity, multicollinearity | 0.21 (0.37) | 0.57 | 0.07 |
|  | T0🡪T2 | 0.23 (-0.09,0.57)^M^ | -0.18 (-0.62,0.62) |  |  | 0.01 (0.37) | 0.97 | < 0.01 |
| **Stroop** (N=35) |  |  |  |  |  |  |  |  |
| Reading baseline, RT [s] | T0🡪T1 | 0.02 (-0.03,0.04) | 0.0 (-0.05,0.03) | age* | homogeneity, multicollinearity, linearity, normality of residuals | -0.002 (0.02) | 0.91 | 0.01 |
|  | T0🡪T2 | 0.01 (0.0,0.04)^M^ | -0.01 (-0.02,0.03) |  |  | -0.008 (0.02) | 0.67 | 0.05 |
| Reading baseline, mistakes | T0🡪T1 | 0 (0,2) | 0 (-1,0) | none | - | -0.94 (0.59) | 0.11 | - |
|  | T0🡪T2 | 0 (-1,1) | 0 (-1,0) |  |  | -0.61 (0.61) | 0.32 |  |
| Reading interference, RT° [s] | T0🡪T1 | -0.03 (-0.09,0.02)^M^ | -0.07 (-0.11,0.0)*^,L^ | age*, education | homogeneity, multicollinearity, normality of REs | -0.02 (0.03) | 0.56 | 0.07 |
|  | T0🡪T2 | 0.01 (-0.07,0.06) | -0.06 (0.11,0.02)^M^ |  |  | -0.04 (0.03) | 0.18 | 0.17 |
| Reading interference, mistakes | T0🡪T1 | 0 (-2,1)^M^ | 0 (-2,0)^M^ | none | - | 0.28 (0.00) | < 0.001* | - |
|  | T0🡪T2 | 0 (-1,2) | -1 (-4,0)^M^ |  |  | -0.56 (0.00) | < 0.001* |  |
| R. interference tendency° [s] | T0🡪T1 | -0.04 (-0.07,0.01)^M^ | -0.04 (-0.11,0.02)^M^ | none | multicollinearity | -0.02 (0.04) | 0.66 | 0.05 |
|  | T0🡪T2 | 0.0 (-0.10,0.05) | -0.05 (-0.12,0.02) |  |  | -0.04 (0.04) | 0.35 | 0.12 |
| Naming baseline, RT [s] | T0🡪T1 | -0.04 (-0.05,0.01)^M^ | -0.01 (-0.03,0.02) | age*, education | multicollinearity, normality of REs | 0.02 (0.02) | 0.39 | 0.11 |
|  | T0🡪T2 | -0.01 (-0.06,0.03) | -0.03 (-0.05,0.02)^M^ |  |  | -0.001 (0.02) | 0.97 | < 0.01 |
| Naming baseline, mistakes | T0🡪T1 | 0 (0,0) | 0 (0,0) | none | - | 0.40 (0.84) | 0.63 | - |
|  | T0🡪T2 | 0 (-1,0) | 0 (0,0) |  |  | 0.23 (0.83) | 0.78 |  |
| Naming interference, RT [s] | T0🡪T1 | -0.01 (-0.07,0.05) | 0.02 (-0.11,0.05) | age* | multicollinearity, linearity, normality of REs | -0.04 (0.08) | 0.63 | 0.06 |
|  | T0🡪T2 | -0.02 (-0.12,0.04) | -0.01 (-0.05,0.02) |  |  | -0.03 (0.08) | 0.72 | 0.04 |
| Naming interference, mistakes | T0🡪T1 | 0.5 (0,2) | 0 (-1,1) | none | - | -1.14 (0.26) | < 0.001* | - |
|  | T0🡪T2 | 0 (-1,0) | 0 (-1,1) |  |  | -1.53 (0.28) | < 0.001* |  |
| N. interference tendency [s] | T0🡪T1 | 0.01 (-0.05,0.06) | 0.03 (-0.06,0.08) | age* | multicollinearity, linearity, normality of REs | -0.06 (0.08) | 0.50 | 0.08 |
|  | T0🡪T2 | 0.02 (-0.10,0.05) | 0.01 (-0.04,0.04) |  |  | -0.03 (0.08) | 0.73 | 0.04 |
| **NBT** (N=35) |  |  |  |  |  |  |  |  |
| RT of correct [s] | T0🡪T1 | 0.03 (-0.07,0.09) | -0.01 (-0.11,0.05) | age* | multicollinearity | -0.10 (0.10) | 0.33 | 0.12 |
|  | T0🡪T2 | 0.01 (-0.06,0.19) | -0.08 (-0.15,0.04)^M^ |  |  | -0.12 (0.10) | 0.25 | 0.14 |
| correct | T0🡪T1 | 1 (-1,1) | 0 (-2,1) | age* | - | -0.01 (0.15) | 0.93 | - |
|  | T0🡪T2 | 1 (1,2)^M^ | 0 (-1,2) |  |  | -0.04 (0.14) | 0.76 |  |
| omissions | T0🡪T1 | -1 (-1,0) | 0 (-1,1) | age* | - | 0.09 (0.28) | 0.75 | - |
|  | T0🡪T2 | -1 (-2,-1)^M^ | 0 (-2,1) |  |  | 0.22 (0.29) | 0.45 |  |
| mistakes | T0🡪T1 | -2 (-4,0)*^,L^ | -1 (-3,0)^M^ | age* | - | 0.39 (0.24) | 0.11 | - |
|  | T0🡪T2 | -1.5 (-4,-1)*^,L^ | -1 (-2,0)^M^ |  |  | 0.60 (0.26) | **0.02*** |  |
| **MRT** (N=35) |  |  |  |  |  |  |  |  |
| RT of correct [s] | T0🡪T1 | -0.80 (-1.83,2.65) | -1.05 (-4.29,2.49) | education* | multicollinearity | -1.14 (1.54) | 0.46 | 0.09 |
|  | T0🡪T2 | 0.15 (-2.59,2.56) | -2.23 (-3.33,2.28) |  |  | -0.81 (1.54) | 0.60 | 0.06 |
| correct | T0🡪T1 | 2 (1,4)*^,L^ | 1 (-1,3)^M^ | none | - | -0.03 (0.10) | 0.74 | - |
|  | T0🡪T2 | 2 (1,3)*^,L^ | 1 (0,3)^M^ |  |  | -0.03 (0.10) | 0.74 |  |
| accuracy | T0🡪T1 | 0.07 (0.03,0.12)*^,L^ | 0.03 (-0.03,0.10)^M^ | none | multicollinearity,  normality of REs | -0.02 (0.03) | 0.39 | 0.11 |
|  | T0🡪T2 | 0.07 (0.03,0.12)*^,L^ | 0.03 (0.0,0.10)*^,M^ |  |  | -0.02 (0.03) | 0.39 | 0.11 |

***Bold*** *& *: significant result. ^M^ / ^L^ : medium / large effect size. ES: effect size, Bravais-Pearson correlation coefficients (r). CM-P: cross-modal phasic. MoCA: Montreal Cognitive Assessment. MRT: Mental Rotation Test. NBT: N-back Test. RE: random effects. RT: reaction time. Stroop: Stroop Interference Test. TMT: Trail-making Test. UM-P: uni-modal phasic.*

**Table S6: Intention-to-treat Analyses of Health-related Quality of Life Outcomes**

| **SIS Domain** (N=43) | **Period** | **Δ Intervention** | **Δ Control** | **Covariates** | **violated Assumptions** | **b (SE)** | **p** | **ES** |
| --- | --- | --- | --- | --- | --- | --- | --- | --- |
| Total score | T0🡪T1 | 2.50 (-1.09,5.39)^M^ | -1.79 (3.79,1.0) | none | normality of REs | -1.14 (1.92) | 0.55 | 0.07 |
|  | T0🡪T2 | 1.44 (-1.51,4.26)^M^ | -1.17 (-3.93,4.72) |  |  | -0.22 (1.92) | 0.91 | 0.01 |
| Strength | T0🡪T1 | 0 (0,6.25) | 0 (-9.38,12.50) | none | homogeneity,  normality of REs | 6.85 (5.53) | 0.22 | 0.14 |
|  | T0🡪T2 | 0 (-6.25,6.25) | 0 (-9.38,15.63) |  |  | 7.35 (5.53) | 0.19 | 0.15 |
| Memory | T0🡪T1 | 3.57 (0,7.14) | 3.57 (-1.79,1.0)^M^ | none | none | 2.50 (3.85) | 0.52 | 0.07 |
|  | T0🡪T2 | 0 (-3.57,7.14) | 0 (-3.57,5.36) |  |  | -0.75 (3.85) | 0.85 | 0.02 |
| Emotions | T0🡪T1 | 0 (-5.56,5.56) | -2.78 (-8.33,0)^M^ | none | none | -1.37 (2.52) | 0.09 | 0.19 |
|  | T0🡪T2 | 0 (-2.78,5.56) | 0 (-5.56,6.94) |  |  | -1.59 (2.52) | 0.53 | 0.07 |
| Communication | T0🡪T1 | 3.57 (0,7.14)^M^ | 3.57 (-3.57,3.57) | none | normality of REs | 1.51 (3.69) | 0.68 | 0.05 |
|  | T0🡪T2 | 3.57 (0,10.71)^M^ | -3.57 (3.57,3.57) |  |  | -0.49 (3.69) | 0.90 | 0.01 |
| Activity | T0🡪T1 | 0 (0,5.0)^M^ | 0 (-3.75,2.50) | none | normality of REs | -1.60 (2.25) | 0.48 | 0.08 |
|  | T0🡪T2 | 0 (0,5.0) | 0 (3.75,1.25) |  |  | -1.91 (2.25) | 0.40 | 0.10 |
| Mobility | T0🡪T1 | 0 (0,2.78)^M^ | 0 (-5.56,0) | none | normality of REs | -4.28 (2.44) | 0.08 | 0.20 |
|  | T0🡪T2 | 2.78 (0,8.33)*^,L^ | 0 (-5.56,2.78) |  |  | -5.39 (2.44) | **0.03*** | 0.24 |
| Hand | T0🡪T1 | 0 (0,0) | 0 (-5.0,0) | age | normality of REs | -4.71 (3.33) | 0.16 | 0.16 |
|  | T0🡪T2 | 0 (0,5.0) | 0 (-5.0,0)^M^ |  |  | -1.11 (3.33) | 0.74 | 0.04 |
| Participation | T0🡪T1 | 0 (0,6.25)^M^ | 0 (-4.69,14.06) | none | normality of REs | -0.55 (4.23) | 0.90 | 0.01 |
|  | T0🡪T2 | 0 (0,3.13) | 0 (-3.13,10.94) |  |  | 4.13 (4.23) | 0.33 | 0.11 |
| Recovery | T0🡪T1 | 2.0 (0,5.0) | 0 (-3.25,1.0) | none | normality of REs | -2.24 (2.94) | 0.45 | 0.09 |
|  | T0🡪T2 | 0 (-5.0,5.0) | 0 (-5.0,5.0) |  |  | 2.05 (2.94) | 0.49 | 0.08 |

***Bold*** *& *: significant result. ^M^ / ^L^ : medium / large effect size. ES: effect size, Bravais-Pearson correlation coefficients (r). RE: random effects.*

**Table S7: Per-protocol Analyses of Health-related Quality of Life Outcomes**

| **SIS Domain** (N=36) | **Period** | **Δ Intervention** | **Δ Control** | **Covariates** | **violated Assumptions** | **b (SE)** | **p** | **ES** |
| --- | --- | --- | --- | --- | --- | --- | --- | --- |
| Total score | T0🡪T1 | 1.81 (-1.23,3.04)^M^ | -1.79 (-3.79,1.0) | none | homogeneity, multicollinearity, normality of REs | -0.76 (2.07) | 0.72 | 0.05 |
|  | T0🡪T2 | 0.0 (-1.65,2.68) | -1.17 (-3.93,4.72) |  |  | 0.92 (2.07) | 0.66 | 0.06 |
| Strength° | T0🡪T1 | 0.0 (-9.38,0.0) | 0.0 (-9.38,12.50) | none | multicollinearity, normality of REs | 8.74 (6.12) | 0.16 | 0.18 |
|  | T0🡪T2 | 0.0 (-9.38,0.0)^M^ | 0.0 (-9.38,15.63) |  |  | 12.40 (6.12) | 0.05 | 0.25 |
| Memory | T0🡪T1 | 3.57 (-5.36,8.93) | 3.57 (-1.79,10.71)^M^ | none | multicollinearity, normality of REs | 2.83 (4.46) | 0.53 | 0.08 |
|  | T0🡪T2 | 0.0 (-5.36,8.93) | 0.0 (-3.57,5.36) |  |  | -1.18 (4.46) | 0.79 | 0.03 |
| Emotions | T0🡪T1 | 0.0 (-4.17,4.17) | -2.78 (-8.33,0.0)^M^ | none | multicollinearity, normality of REs | -3.69 (2.61) | 0.16 | 0.17 |
|  | T0🡪T2 | 0.0 (-2.78,5.56) | 0.0 (-5.56,6.94) |  |  | -1.17 (2.61) | 0.66 | 0.06 |
| Communication | T0🡪T1 | 3.57 (0.0,5.36)^M^ | 3.57 (-3.57,3.57) | none | multicollinearity, normality of REs | 1.85 (3.94) | 0.64 | 0.06 |
|  | T0🡪T2 | 3.57 (-1.79,10.71) | -3.57 (-3.57,3.57) |  |  | 1.12 (3.94) | 0.78 | 0.04 |
| Activity | T0🡪T1 | 2.50 (0.0,5.0)^M^ | 0.0 (-3.75,2.50) | none | multicollinearity, normality of REs | -1.94 (2.41) | 0.43 | 0.10 |
|  | T0🡪T2 | 0.0 (-2.50,1.25) | 0.0 (-3.75,1.25) |  |  | 0.09 (2.41) | 0.97 | < 0.01 |
| Mobility | T0🡪T1 | 0.0 (0.0,4.17) | 0.0 (-5.56,0.0) | none | multicollinearity, normality of REs | -3.79 (2.67) | 0.16 | 0.17 |
|  | T0🡪T2 | 2.78 (0.0,6.94)*^,L^ | 0.0 (-5.56,2.78) |  |  | -5.16 (2.67) | 0.06 | 0.23 |
| Hand | T0🡪T1 | 0.0 (-5.0,0.0) | 0.0 (-5.0,0.0) | none | multicollinearity, normality of REs | -1.49 (3.57) | 0.68 | 0.05 |
|  | T0🡪T2 | 0.0 (-7.50,0.0)^M^ | 0.0 (-5.0,0.0)^M^ |  |  | 2.91 (3.57) | 0.42 | 0.10 |
| Participation | T0🡪T1 | 0.0 (0.0,6.25)^M^ | 0.0 (-4.69,14.06) | none | homogeneity, multicollinearity, normality of REs | -2.10 (4.17) | 0.62 | 0.06 |
|  | T0🡪T2 | 0.0 (0.0,1.56) | 0.0 (-3.13,10.94) |  |  | 3.59 (4.17) | 0.39 | 0.11 |
| Recovery | T0🡪T1 | 2.0 (0.0,5.0)^M^ | 0.0 (-3.25,1.0) | none | multicollinearity, normality of REs | -3.84 (2.89) | 0.19 | 0.16 |
|  | T0🡪T2 | 0.0 (-2.50,5.50) | 0.0 (-5.0,5.0) |  |  | -0.10 (2.89) | 0.97 | < 0.01 |

***Bold*** *& *: significant result. ° significant baseline difference. ^M^ / ^L^ : medium / large effect size. ES: effect size, Bravais-Pearson correlation coefficients (r). RE: random effects.*

**Table S8: Intention-to-treat Analyses Motor Outcomes**

| **Outcomes** | **Period** | **Δ Intervention** | **Δ Control** | **Covariates** | **violated Assumptions** | **b (SE)** | **p** | **ES** |
| --- | --- | --- | --- | --- | --- | --- | --- | --- |
| **TUG** (N=46) |  |  |  |  |  |  |  |  |
| time [s] | T0🡪T1 | 0.0 (-0.67,0.0)*^,L^ | -0.33 (-0.67,0.0)*^,M^ | FMA* | linearity, normality of  residuals & RE | 1.29 (1.05) | 0.22 | 0.13 |
|  | T0🡪T2 | 0.0 (-0.67,0.33) | -0.33 (-0.33,0.25)^M^ |  |  | 0.13 (1.05) | 0.90 | 0.01 |
| **TUG-Cogn** (N=45) |  |  |  |  |  |  |  |  |
| time [s] | T0🡪T1 | -0.67 (-1.67,0.0)*^,M^ | -1.0 (-2.0,-0.33)*^,M^ | age*, FMA* | linearity, normality of res. | 0.21 (1.18) | 0.86 | 0.02 |
|  | T0🡪T2 | -0.50 (-2.17,0.0)^M^ | -1.33 (-2.0,-0.67)*^,L^ |  |  | 0.20 (1.18) | 0.87 | 0.02 |
| motor Dual-Task Effect° | T0🡪T1 | 0.0 (-14.62,6.39) | -3.89 (-21.43,3.33)^M^ | age* | linearity, normality of  residuals & RE | -2.02 (8.02) | 0.80 | 0.03 |
|  | T0🡪T2 | -7.59 (-18.13,0.0)^M^ | -15.0 (-28.41,-0.91)*^,L^ |  |  | 0.18 (8.02) | 0.98 | < 0.01 |
| CRR single-task | T0🡪T1 | 0.04 (0.0,0.09)*^,L^ | 0.03 (0.0,0.10)*^,M^ | none | none | 0.001 (0.02) | 0.98 | < 0.01 |
|  | T0🡪T2 | 0.05 (0.0,0.13)*^,L^ | 0.07 (0.02,0.10)*^,L^ |  |  | -0.0004 (0.02) | 0.99 | < 0.01 |
| CRR dual-task | T0🡪T1 | 0.04 (-0.01,0.13)*^,M^ | 0.0 (-0.01,0.03) | none | none | -0.04 (0.04) | 0.21 | 0.14 |
|  | T0🡪T2 | 0.08 (0.0,0.15)*^,L^ | 0.01 (-0.02,0.09) |  |  | -0.05 (0.04) | 0.13 | 0.16 |
|  |  |  |  |  |  |  |  |  |
| **10MWT** (N=46) |  |  |  |  |  |  |  |  |
| time [s] | T0🡪T1 | -0.17 (-1.0, 0.33)^M^ | -0.33 (-1.0,0.33)^M^ | FMA* | linearity, normality of  residuals & RE | 0.88 (0.87) | 0.31 | 0.11 |
|  | T0🡪T2 | 0.0 (-0.50,0.0) | 0.0 (-0.58,0.25) |  |  | -0.47 (0.87) | 0.59 | 0.06 |
| speed [m/s] | T0🡪T1 | 0.05 (-0.02,0.12)^M^ | 0.05 (-0.05,0.10) | age*, FMA* | none | -0.01 (0.03) | 0.72 | 0.04 |
|  | T0🡪T2 | 0.02 (-0.01,0.08)*^,M^ | 0.01 (-0.03,0.11) |  |  | 0.02 (0.03) | 0.53 | 0.07 |
| cadence [steps/min] | T0🡪T1 | 1.10 (-2.32,4.36) | 1.86 (-2.59,5.15) | FMA* | none | 0.67 (1.74) | 0.70 | 0.04 |
|  | T0🡪T2 | 2.49 (-0.25,3.0)^M^ | 2.36 (-0.88,5.53)^M^ |  |  | 2.14 (1.74) | 0.22 | 0.13 |
| stride length affected [m] | T0🡪T1 | 0.05 (-0.01,0.07)*^,M^ | 0.03 (0.0,0.08)^M^ | age, FMA* | none | -0.01 (0.02) | 0.77 | 0.03 |
|  | T0🡪T2 | 0.01 (0.0,0.08)*^,M^ | 0.03 (-0.03,0.08)^M^ |  |  | 0.01 (0.02) | 0.79 | 0.03 |
| stride length unaffected [m] | T0🡪T1 | 0.01 (-0.01,0.07)^M^ | 0.02 (-0.01,0.08) | age, FMA* | none | -0.003 (0.02) | 0.88 | 0.02 |
|  | T0🡪T2 | 0.01 (0.0,0.04)^M^ | 0.0 (-0.05,0.04) |  |  | 0.001 (0.02) | 0.98 | < 0.01 |
| stride time affected [s] | T0🡪T1 | 0.0 (-0.04,0.02) | -0.02 (-0.05,0.03) | FMA* | linearity, normality of  residuals & RE | -0.06 (0.05) | 0.22 | 0.13 |
|  | T0🡪T2 | -0.02 (-0.03,0.0)^M^ | -0.04 (-0.09,0.02)^M^ |  |  | -0.03 (0.05) | 0.59 | 0.06 |
| stride time unaffected [s] | T0🡪T1 | -0.01 (-0.05,0.03)*^,L^ | -0.05 (-0.11,0.02)^M^ | FMA* | linearity, normality of  residuals & RE | -0.12 (0.08) | 0.16 | 0.16 |
|  | T0🡪T2 | -0.03 (-0.05,0.0) | -0.04 (-0.09,0.01)^M^ |  |  | -0.04 (0.08) | 0.60 | 0.06 |
| double-support time [%] | T0🡪T1 | 0.0 (-2.62,1.51) | -0.56 (-2.99,0.25) | FMA* | linearity | -0.47 (1.02) | 0.65 | 0.05 |
|  | T0🡪T2 | 0.0 (-1.44,0.55) | -0.23 (-0.96,0.43) |  |  | 0.01 (1.01) | 0.99 | < 0.01 |
| stance time affected [%] | T0🡪T1 | 0.0 (-0.70,0.60) | -0.26 (-0.96,0.65) | FMA* | linearity | -0.26 (0.59) | 0.65 | 0.05 |
|  | T0🡪T2 | 0.0 (-0.74,0.56) | 0.26 (-0.48,0.85) |  |  | 0.25 (0.59) | 0.67 | 0.05 |
| stance time unaffected [%] | T0🡪T1 | 0.0 (-1.20,0.92) | -0.32 (-2.33,0.44)^M^ | FMA* | linearity | -0.14 (0.65) | 0.83 | 0.02 |
|  | T0🡪T2 | 0.0 (-1.09,0.53) | -0.26 (-1.91,0.12)^M^ |  |  | -0.05 (0.65) | 0.94 | < 0.01 |
| swing time affected [%] | T0🡪T1 | 0.0 (-0.60,0.70) | 0.26 (-0.65,0.96) | FMA* | linearity | 0.27 (0.59) | 0.65 | 0.05 |
|  | T0🡪T2 | 0.0 (-0.56,0.74) | -0.26 (-0.85,0.48) |  |  | -0.25 (0.59) | 0.67 | 0.05 |
| swing time unaffected [%] | T0🡪T1 | 0.0 (-0.92,1.20) | 0.32 (-0.44,2.33)^M^ | FMA* | linearity | 0.11 (0.65) | 0.87 | 0.02 |
|  | T0🡪T2 | 0.0 (-0.53,1.09) | 0.26 (-0.34,1.91) |  |  | 0.02 (0.65) | 0.97 | < 0.01 |
| swing width affected [cm] | T0🡪T1 | 0.05 (-0.17,0.37) | -0.27 (-0.93,0.43) | none | none | -0.17 (0.31) | 0.59 | 0.06 |
|  | T0🡪T2 | 0.0 (-0.42,0.43) | -0.08 (-0.79,0.58) |  |  | 0.03 (0.31) | 0.93 | < 0.01 |
| swing width unaffected° [cm] | T0🡪T1 | 0.18 (-0.03,0.80)^M^ | -0.10 (-0.47,0.30) | FMA | linearity, normality of  residuals & RE | -0.32 (0.31) | 0.30 | 0.11 |
|  | T0🡪T2 | 0.03 (-0.08,0.58)^M^ | 0.12 (-0.35,0.78) |  |  | -0.31 (0.31) | 0.31 | 0.11 |
| asymmetry [%] | T0🡪T1 | 0.0 (-2.76,1.02) | -0.47 (-3.70,1.40) | FMA* | homogeneity, linearity | 0.75 (1.88) | 0.69 | 0.04 |
|  | T0🡪T2 | 0.0 (-0.98,1.34) | -0.80 (-3.94,0.74)^M^ |  |  | -1.0 (1.88) | 0.59 | 0.06 |
| GVI affected | T0🡪T1 | 1.54 (-1.92,10.85) | 2.96 (-2.35,9.22)^M^ | FMA* | homogeneity | 0.22 (3.98) | 0.96 | < 0.01 |
|  | T0🡪T2 | -0.54 (-2.90,4.23) | 1.23 (-1.35,6.47) |  |  | -0.63 (3.98) | 0.87 | 0.02 |
| GVI unaffected | T0🡪T1 | 0.0 (-3.52,12.48) | 5.92 (-2.43,17.47) | FMA* | multicollinearity, normality of RE | -0.72 (5.35) | 0.89 | 0.01 |
|  | T0🡪T2 | -0.70 (-9.09,0.61) | 3.79 (-4.34,12.22) |  |  | 4.83 (5.34) | 0.37 | 0.10 |
| walk ratio [cm/steps/min] | T0🡪T1 | 0.0 (-0.01,0.02) | 0.0 (-0.02,0.02) | FMA* | homogeneity, linearity | -0.01 (0.01) | 0.53 | 0.07 |
|  | T0🡪T2 | 0.0 (-0.02, 0.01) | -0.01 (-0.03,0.01) |  |  | -0.01 (0.01) | 0.29 | 0.11 |
| **10MWT fast** (N=43) |  |  |  |  |  |  |  |  |
| time [s] | T0🡪T1 | -0.17 (-1.25,0.0)*^,L^ | -0.33 (-1.0,0.0)*^,M^ | age*, FMA* | linearity, normality of residuals | 0.51 (0.46) | 0.27 | 0.13 |
|  | T0🡪T2 | 0.0 (-1.33,0.0)*^,M^ | -0.17 (-1.0,0.33) |  |  | 0.14 (0.45) | 0.75 | 0.04 |
| **OWA** (N=43) |  |  |  |  |  |  |  |  |
| speed [m/s] | T0🡪T1 | 0.01 (-0.02,0.10)^M^ | 0.0 (-0.10,0.03) | FMA* | none | -0.07 (0.03) | **0.02*** | 0.25 |
|  | T0🡪T2 | 0.0 (-0.01,0.04)^M^ | -0.03 (-0.06,0.10) |  |  | -0.03 (0.03) | 0.33 | 0.11 |
| cadence [steps/min] | T0🡪T1 | 1.62 (0.0,4.69)*^,L^ | 0.23 (-1.46,2.40) | FMA* | linearity | -2.57 (1.35) | 0.06 | 0.21 |
|  | T0🡪T2 | 0.24 (-0.04,3.83)*^,M^ | 0.37 (-1.60,3.52) |  |  | -0.33 (1.33) | 0.81 | 0.03 |
| stride length affected [m] | T0🡪T1 | 0.0 (-0.02,0.04) | 0.0 (-0.07,0.03) | FMA* | none | -0.03 (0.02) | 0.16 | 0.16 |
|  | T0🡪T2 | 0.0 (-0.02,0.06) | 0.01 (-0.05,0.05) |  |  | -0.02 (0.02) | 0.36 | 0.10 |
|  |  |  |  |  |  |  |  |  |
| stride length unaffected [m] | T0🡪T1 | 0.0 (-0.03,0.05) | -0.01 (-0.07,0.03) | FMA* | none | -0.03 (0.02) | 0.26 | 0.13 |
|  | T0🡪T2 | 0.0 (-0.03,0.05) | -0.02 (-0.07,0.04) |  |  | -0.01 (0.02) | 0.50 | 0.07 |
| stride time affected [s] | T0🡪T1 | -0.02 (-0.05,0.0)*^,M^ | 0.0 (-0.03,0.02) | FMA* | linearity, normality of REs | 0.02 (0.01) | 0.19 | 0.15 |
|  | T0🡪T2 | -0.01 (-0.04,0.0)*^,L^ | -0.01 (-0.06,0.02) |  |  | -0.001 (0.01) | 0.95 | < 0.01 |
| stride time unaffected [s] | T0🡪T1 | -0.01 (-0.04,0.0)*^,M^ | 0.0 (-0.03,0.02) | FMA* | linearity, normality of REs | 0.02 (0.01) | 0.23 | 0.13 |
|  | T0🡪T2 | -0.02 (0.04,0.0)*^,M^ | -0.01 (-0.06,0.02) |  |  | -0.003 (0.01) | 0.85 | 0.02 |
| double-support time [%] | T0🡪T1 | 0.25 (-1.21,1.77) | 0.0 (-2.15,1.34) | FMA* | linearity | -0.50 (0.90) | 0.58 | 0.06 |
|  | T0🡪T2 | 0.05 (-0.16,1.35)^M^ | -0.32 (-1.76,0.52) |  |  | -0.60 (0.89) | 0.50 | 0.08 |
| stance time affected [%] | T0🡪T1 | 0.35 (-0.40,0.77) | 0.0 (-1.25,0.90) | FMA* | linearity, normality of REs | -0.84 (0.62) | 0.18 | 0.15 |
|  | T0🡪T2 | 0.09 (-0.07,0.59)^M^ | -0.19 (-0.84,0.55) |  |  | -0.65 (0.61) | 0.29 | 0.12 |
| stance time unaffected [%] | T0🡪T1 | 0.0 (-0.37,1.03) | 0.22 (-0.53,0.80) | FMA* | linearity | 0.29 (0.49) | 0.56 | 0.06 |
|  | T0🡪T2 | 0.0 (-0.49,0.79) | -0.25 (-0.93,0.31) |  |  | 0.02 (0.49) | 0.97 | < 0.01 |
| swing time affected [%] | T0🡪T1 | -0.34 (-0.77,0.40)^M^ | 0.0 (-0.90,1.25) | FMA* | linearity, normality of REs | 0.84 (0.62) | 0.18 | 0.15 |
|  | T0🡪T2 | -0.09 (-0.59,0.07) | 0.19 (-0.55,0.84) |  |  | 0.65 (0.61) | 0.29 | 0.12 |
| swing time unaffected [%] | T0🡪T1 | 0.0 (-1.03,0.37) | -0.22 (-0.80,0.53) | FMA* | linearity | -0.29 (0.49) | 0.56 | 0.06 |
|  | T0🡪T2 | 0.0 (-0.79,0.49) | 0.25 (-0.31,0.93) |  |  | 0.02 (0.49) | 0.97 | < 0.01 |
| swing width affected [cm] | T0🡪T1 | 0.0 (-0.97,0.52) | -0.30 (-0.90,0.05) | none | none | 0.22 (0.39) | 0.57 | 0.06 |
|  | T0🡪T2 | -0.05 (-0.75,0.33) | -0.35 (-1.13,0.0)^M^ |  |  | -0.05 (0.38) | 0.89 | 0.02 |
| swing width unaffected [cm] | T0🡪T1 | 0.0 (-0.28,0.38) | -0.40 (-0.80,-0.25)*^,L^ | FMA* | none | -0.80 (0.27) | **0.004*** | 0.31 |
|  | T0🡪T2 | 0.30 (0.0,0.58)*^,L^ | -0.25 (-0.85,0.23)^M^ |  |  | -0.84 (0.27) | **0.003*** | 0.33 |
| asymmetry | T0🡪T1 | 0.80 (0.0,2.76)^M^ | 0.60 (-1.75,2.65) | FMA* | homogeneity, linearity, normality of residuals & REs | 1.50 (1.64) | 0.36 | 0.10 |
|  | T0🡪T2 | 0.0 (-1.12,0.85) | 0.50 (-0.65,1.78) |  |  | 1.91 (1.63) | 0.24 | 0.13 |
| GVI affected | T0🡪T1 | -0.31 (-3.70,5.37) | -1.37 (-9.21,4.21) | FMA* | normality of REs | -3.54 (3.25) | 0.28 | .012 |
|  | T0🡪T2 | -0.01 (-4.28,6.01) | 0.59 (-8.53,5.55) |  |  | -1.79 (3.22) | 0.58 | 0.06 |
| GVI unaffected | T0🡪T1 | 0.62 (-1.47,3.32) | -0.94 (-5.24,4.61) | FMA* | none | -1.87 (2.81) | 0.51 | 0.07 |
|  | T0🡪T2 | 0.89 (1.78,6.63) | 1.41 (-2.69,11.05) |  |  | 1.27 (2.79) | 0.65 | 0.05 |
| walk ratio [cm/steps/min] | T0🡪T1 | 0.0 (-0.03,0.02) | -0.01 (0.02,0.01) | FMA* | none | 0.001 (0.01) | 0.93 | 0.01 |
|  | T0🡪T2 | 0.0 (-0.02,0.02) | -0.01 (-0.02,0.01)^M^ |  |  | -0.01 (0.01) | 0.41 | 0.09 |

***Bold*** *& *: significant result. ° significant baseline different. ^M^ / ^L^ : medium / large effect size. ES: effect size, Bravais-Pearson correlation coefficients (r). CRR: Correct Response Rate. GVI: Gait Variability Index. OWA: Outdoor Walking Assessment. RE: random effects. TUG: Timed-up-and-go Test. TUG-Cogn: cognitive dual-task TUG. 10MWT: 10-Meter Walk Test.*

**Table S9: Per-protocol Analyses of Motor Outcomes**

| **Outcome** | **Period** | **Δ Intervention** | **Δ Control** | **Covariates** | **violated Assumptions** | **b (SE)** | **p** | **ES** |
| --- | --- | --- | --- | --- | --- | --- | --- | --- |
| **TUG** (N=36) |  |  |  |  |  |  |  |  |
| time [s] | T0🡪T1 | -0.67 (-0.67,0.0)*^,L^ | -0.33 (-0.75,0.0)*^,M^ | FMA* | multicollinearity, linearity, normality of res. & REs | 1.49 (1.25) | 0.24 | 0.15 |
|  | T0🡪T2 | -0.33 (-1.17,0.25) | -0.33 (-0.33,0.33)^M^ |  |  | 0.17 (1.26) | 0.89 | 0.02 |
| **TUG-Cogn** (N=35) |  |  |  |  |  |  |  |  |
| time | T0🡪T1 | -0.83 (-2.0,0.0)^M^ | -1.17 (-2.25,0.58)*^,M^ | age*, FMA* | multicollinearity, linearity, normality of res. | -0.53 (1.24) | 0.67 | 0.05 |
|  | T0🡪T2 | -0.67 (-2.33,0.0)^M^ | -1.33 (-2.0,-0.67)*^,L^ |  |  | -0.70 (1.25) | 0.58 | 0.07 |
| motor Dual-Task Effect° | T0🡪T1 | -7.50 (-17.51,8.18) | -6.81 (-22.32,3.33)^M^ | age | multicollinearity, linearity, normality of res. & REs | -2.02 (9.64) | 0.83 | 0.03 |
|  | T0🡪T2 | -8.33 (-15.0,4.17)^M^ | -16.67 (-30.74,3.63)*^,L^ |  |  | -2.11 (9.72) | 0.83 | 0.03 |
| CRR single-task° | T0🡪T1 | 0.06 (0.02,0.11)*^,L^ | 0.03 (0.01,0.11)*^,M^ | none | multicollinearity | -0.01 (0.03) | 0.72 | 0.05 |
|  | T0🡪T2 | 0.12 (0.02,0.14)*^,L^ | 0.07 (0.02,0.10)*^,L^ |  |  | -0.01 (0.03) | 0.63 | 0.06 |
| CRR dual-task | T0🡪T1 | 0.07 (-0.02,0.17)^M^ | 0.0 (-0.02,0.04) | none | multicollinearity, normality of REs | -0.04 (0.04) | 0.28 | 0.13 |
|  | T0🡪T2 | 0.11 (0.02,0.13)^L^ | 0.02 (-0.02,0.10) |  |  | -0.06 (0.04) | 0.14 | 0.18 |
|  |  |  |  |  |  |  |  |  |
|  |  |  |  |  |  |  |  |  |
| **10MWT** (N=36) |  |  |  |  |  |  |  |  |
| time [s] | T0🡪T1 | 0.0 (-1.0,0.50) | -0.33 (-1.08,0.33)^M^ | FMA* | multicollinearity, linearity, normality of res. & REs | 1.05 (1.11) | 0.35 | 0.12 |
|  | T0🡪T2 | -0.17 (-0.67,0.25) | 0.0 (-0.67,0.33) |  |  | -0.56 (1.11) | 0.62 | 0.06 |
| speed [m/s] | T0🡪T1 | 0.05 (-0.08,0.07) | 0.05 (-0.05,0.12) | age*, FMA* | multicollinearity | 0.01 (0.04) | 0.77 | 0.04 |
|  | T0🡪T2 | 0.03 (-0.02,0.07)^M^ | 0.01 (-0.03,0.12) |  |  | 0.03 (0.04) | 0.46 | 0.09 |
| cadence [steps/min] | T0🡪T1 | -1.38 (-3.61,4.20) | 2.0 (-2.70,6.08) | FMA* | multicollinearity | 1.43 (2.23) | 0.52 | 0.08 |
|  | T0🡪T2 | 2.64 (-0.65,4.19)^M^ | 3.05 (-0.94,5.65)*^,M^ |  |  | 1.93 (2.24) | 0.39 | 0.11 |
| stride length affected [m] | T0🡪T1 | 0.05 (-0.04,0.07)^M^ | 0.04 (-0.01,0.08)^M^ | FMA* | multicollinearity, normality of REs | 0.01 (0.03) | 0.81 | 0.03 |
|  | T0🡪T2 | 0.02 (-0.01,0.08)^M^ | 0.03 (-0.03,0.09)^M^ |  |  | 0.01 (0.03) | 0.69 | 0.05 |
| stride length unaffected [m] | T0🡪T1 | 0.01 (-0.04,0.05) | 0.02 (-0.01,0.08) | FMA* | multicollinearity | 0.01 (0.03) | 0.59 | 0.07 |
|  | T0🡪T2 | 0.01 (-0.03,0.04) | 0.0 (-0.05,0.04) |  |  | 0.01 (0.03) | 0.74 | 0.04 |
| stride time affected [s] | T0🡪T1 | 0.02 (-0.04,0.04) | -0.03 (-0.07,0.03) | FMA* | multicollinearity, linearity, normality of res. & REs | -0.09 (0.06) | 0.13 | 0.19 |
|  | T0🡪T2 | -0.02 (-0.04,0.01)^M^ | -0.04 (-0.09,0.02)^M^ |  |  | -0.03 (0.06) | 0.66 | 0.06 |
| stride time unaffected [s] | T0🡪T1 | 0.01 (-0.04,0.04) | -0.05 (-0.11,0.02)^M^ | FMA* | multicollinearity, linearity, normality of res. & REs | -0.18 (0.11) | 0.10 | 0.22 |
|  | T0🡪T2 | -0.03 (-0.06,0.0)*^,L^ | -0.04 (-0.10,0.01)^M^ |  |  | -0.05 (0.11) | 0.68 | 0.05 |
| double-support time [%] | T0🡪T1 | 0.77 (-1.82,2.05) | -0.71 (-3.03,0.55)^M^ | FMA* | multicollinearity, linearity | -1.67 (1.07) | 0.12 | 0.19 |
|  | T0🡪T2 | -0.04 (-1.46,0.67) | -0.27 (-0.97,0.45) |  |  | -0.74 (1.08) | 0.50 | 0.08 |
| stance time affected [%] | T0🡪T1 | 0.26 (-0.34,1.12) | -0.32 (-0.97,0.70) | FMA* | multicollinearity, linearity | -0.79 (0.67) | 0.24 | 0.14 |
|  | T0🡪T2 | 0.11 (-0.69,1.12) | 031 (-0.53,0.90) |  |  | -0.24 (0.67) | 0.72 | 0.04 |
| stance time unaffected [%] | T0🡪T1 | 0.85 (-0.93,1.08) | -0.74 (-2.36,0.55)^M^ | FMA* | multicollinearity | -0.90 (0.64) | 0.16 | 0.17 |
|  | T0🡪T2 | -0.12 (-1.05,0.91) | -0.32 (-1.97,0.16)^M^ |  |  | -0.39 (0.64) | 0.54 | 0.07 |
| swing time affected [%] | T0🡪T1 | -0.26 (-1.12,0.34) | 0.32 (-0.70,0.97) | FMA* | multicollinearity, linearity | 0.79 (0.67) | 0.24 | 0.15 |
|  | T0🡪T2 | -0.11 (-1.12,0.69) | -0.31 (-0.90,0.53) |  |  | 0.24 (0.67) | 0.72 | 0.04 |
| swing time unaffected° [%] | T0🡪T1 | -0.85 (1.08,0.93) | 0.58 (-0.55,2.36)^M^ | FMA* | multicollinearity | 0.86 (0.64) | 0.18 | 0.16 |
|  | T0🡪T2 | 0.12 (-0.91,1.05) | 0.32 (-0.40,1.97) |  |  | 0.36 (0.64) | 0.58 | 0.07 |
| swing width affected [cm] | T0🡪T1 | 0.17 (-0.20,0.57) | -0.27 (-0.97,0.46) | FMA* | multicollinearity | -0.25 (0.35) | 0.48 | 0.09 |
|  | T0🡪T2 | 0.0 (-0.44,0.36) | -0.17 (-0.83,0.60) |  |  | 0.10 (0.36) | 0.79 | 0.03 |
| swing width unaffected [cm] | T0🡪T1 | 0.20 (-0.20,0.57)^M^ | -0.13 (-0.51,0.31) | FMA* | multicollinearity | -0.38 (0.29) | 0.19 | 0.16 |
|  | T0🡪T2 | 0.20 (-0.17,0.68) | 0.23 (-0.37,0.90) |  |  | -0.32 (0.29) | 0.27 | 0.14 |
| asymmetry° [%] | T0🡪T1 | 0.40 (-2.58,1.43) | -0.68 (-3.76,1.44) | FMA* | homogeneity, multicollinearity, linearity, normality of REs | -0.21 (2.05) | 0.92 | 0.01 |
|  | T0🡪T2 | 0.70 (-0.46,2.23) | -0.87 (-3.94,0.80)^M^ |  |  | -1.10 (2.07) | 0.60 | 0.06 |
| GVI affected | T0🡪T1 | 3.53 (-6.52,7.65) | 3.03 (-2.48,10.20)^M^ | FMA* | multicollinearity, normality of REs | 1.89 (4.73) | 0.69 | 0.05 |
|  | T0🡪T2 | -1.92 (3.42,2.72) | 1.52 (-1.37,7.01) |  |  | 1.68 (4.76) | 0.73 | 0.04 |
| GVI unaffected | T0🡪T1 | -1.81 (-9.83,9.64) | 6.63 (-3.56,18.83) | FMA* | multicollinearity, normality of REs | 1.94 (6.49) | 0.77 | 0.04 |
|  | T0🡪T2 | -4.51 (-15.22,0.63)^M^ | 4.49 (-5.78,12.39) |  |  | 9.35 (6.53) | 0.16 | 0.17 |
| walk ratio  [cm/steps/min] | T0🡪T1 | 0.0 (-0.01,0.01) | 0.0 (-0.02,0.02) | FMA* | multicollinearity, normality of REs | -0.003 (0.01) | 0.77 | 0.04 |
|  | T0🡪T2 | -0.02 (-0.02,0.01) | -0.01 (-0.03,0.01) |  |  | -0.005 (0.01) | 0.65 | 0.06 |
| **10MWT fast** (N=34) |  |  |  |  |  |  |  |  |
| time | T0🡪T1 | -0.50 (-1.50,0.0)*^,L^ | -0.33 (-1.0,0.0)*^,M^ | age*, FMA* | multicollinearity, linearity | 0.65 (0.57) | 0.26 | 0.15 |
|  | T0🡪T2 | -0.67 (-1.33,0.33)^M^ | -0.33 (-1.0,0.33) |  |  | 0.28 (0.58) | 0.63 | 0.06 |
| **OWA** (N=35) |  |  |  |  |  |  |  |  |
| speed [m/s] | T0🡪T1 | 0.01 (-0.02,0.07) | -0.01 (-0.11,0.03) | FMA* | multicollinearity | -0.61 (0.04) | 0.11 | 0.20 |
|  | T0🡪T2 | 0.0 (-0.02,0.04) | -0.04 (-0.06,0.11) |  |  | -0.02 (0.04) | 0.56 | 0.07 |
| cadence [steps/min] | T0🡪T1 | 1.62 (-0.94,5.14)^M^ | 0.45 (-1.55,2.42) | FMA* | multicollinearity | -2.58 (1.69) | 0.13 | 0.19 |
|  | T0🡪T2 | 1.70 (-1.05,3.83)^M^ | 0.56 (-1.69,4.08) |  |  | -0.62 (1.67) | 0.71 | 0.05 |
| stride length affected [m] | T0🡪T1 | 0.01 (-0.04,0.04) | -0.01 (-0.07,0.04) | FMA* | multicollinearity | -0.02 (0.03) | 0.42 | 0.10 |
|  | T0🡪T2 | 0.0 (-0.04,0.04) | 0.01 (-0.05,0.06) |  |  | -0.01 (0.02) | 0.59 | 0.07 |
| stride length unaffected [m] | T0🡪T1 | -0.01 (-0.08,0.04) | -0.02 (-0.07,0.04) | FMA* | multicollinearity | -0.01 (0.03) | 0.68 | 0.05 |
|  | T0🡪T2 | -0.03 (-0.05,0.05) | -0.02 (-0.07,0.05) |  |  | -0.005 (0.03) | 0.86 | 0.02 |
| stride time affected [s] | T0🡪T1 | -0.02 (-0.05,0.01)^M^ | 0.0 (-0.03,0.02) | FMA* | multicollinearity, linearity, normality of REs | 0.01 (0.02) | 0.42 | 0.10 |
|  | T0🡪T2 | -0.02 (-0.04,0.01)^M^ | -0.02 (-0.06,0.02) |  |  | 0.001 (0.02) | 0.94 | 0.01 |
| stride time unaffected [s] | T0🡪T1 | -0.01 (-0.05,0.01) | 0.0 (-0.03,0.02) | FMA* | multicollinearity, linearity, normality of REs | 0.01 (0.02) | 0.44 | 0.10 |
|  | T0🡪T2 | -0.02 (-0.04,0.01)^M^ | -0.02 (-0.06,0.02) |  |  | 0.001 (0.02) | 0.95 | 0.01 |
| double-support time [%] | T0🡪T1 | 1.02 (-1.41,1.77) | -0.04 (-2.31,1.38) | FMA* | multicollinearity, linearity | -0.87 (1.12) | 0.44 | 0.10 |
|  | T0🡪T2 | 0.68 (-0.16,1.63)^M^ | -0.45 (-2.0,0.67) |  |  | -0.99 (0.11) | 0.37 | 0.11 |
| stance time affected [%] | T0🡪T1 | 0.43 (-0.32,1.18) | 0.01 (-1.26,0.98) | FMA* | multicollinearity, linearity, normality of REs | -1.18 (0.76) | 0.13 | 0.19 |
|  | T0🡪T2 | 0.36 (-0.07,0.59)^M^ | -0.20 (-0.91,0.61) |  |  | -0.90 (0.76) | 0.24 | 0.15 |
| stance time unaffected [%] | T0🡪T1 | 0.16 (-1.06,1.55) | 0.24 (-0.54,0.89) | FMA* | multicollinearity, linearity | 0.24 (0.61) | 0.70 | 0.05 |
|  | T0🡪T2 | 0.29 (-0.32,0.86)^M^ | -0.28 (-1.01,0.33) |  |  | -0.15 (0.60) | 0.80 | 0.03 |
| swing time affected [%] | T0🡪T1 | -0.43 (-1.18,0.32) | -0.01 (-0.98,1.26) | FMA* | multicollinearity, linearity, normality of REs | 1.18 (0.76) | 0.13 | 0.19 |
|  | T0🡪T2 | -0.36 (-0.59,0.06)^M^ | 0.20 (-0.61,0.91) |  |  | 0.90 (0.76) | 0.24 | 0.15 |
| swing time unaffected [%] | T0🡪T1 | -0.16 (-1.55,1.06) | -0.24 (-0.89,0.54) | FMA* | multicollinearity, linearity | -0.24 (0.61) | 0.70 | 0.05 |
|  | T0🡪T2 | -0.30 (-0.86,0.32)^M^ | 0.28 (-0.33,1.01) |  |  | 0.15 (0.60) | 0.80 | 0.03 |
| swing width affected [cm] | T0🡪T1 | -0.10 (-0.98,0.95) | -0.30 (-0.90,0.07) | none | multicollinearity | -0.05 (0.43) | 0.91 | 0.01 |
|  | T0🡪T2 | -0.45 (-0.75,0.70) | -0.40 (-1.15,-0.05)^M^ |  |  | -0.25 (0.43) | 0.57 | 0.07 |
| swing width unaffected [cm] | T0🡪T1 | 0.20 (-0.28,0.38) | -0.45 (-0.85,-0.30)*^,L^ | none | multicollinearity | -0.73 (0.30) | **0.02*** | 0.29 |
|  | T0🡪T2 | 0.30 (0.13,0.58)^M^ | -0.30 (-0.90,0.25)^M^ |  |  | -0.84 (0.30) | **0.007*** | 0.33 |
| asymmetry | T0🡪T1 | 1.76 (0.55,2.76)^M^ | 0.75 (-1.83,2.73) | FMA* | homogeneity, multicollinearity, linearity | 2.10 (2.07) | 0.32 | 0.13 |
|  | T0🡪T2 | 0.40 (-1.34,1.22) | 0.50 (-0.80,1.95) |  |  | 2.39 (2.06) | 0.25 | 0.15 |
| GVI affected | T0🡪T1 | -3.12 (-5.23,3.06) | -2.21 (-9.86,3.59) | FMA* | multicollinearity, normality of REs | -0.73 (3.71) | 0.85 | 0.02 |
|  | T0🡪T2 | 2.44 (-4.28,7.13) | -2.13 (-9.29,4.53) |  |  | -3.53 (3.68) | 0.34 | 0.12 |
| GVI unaffected | T0🡪T1 | -0.77 (-3.49,3.32) | -1.48 (-6.10,3.24) | age*, FMA* | multicollinearity | 0.75 (3.13) | 0.81 | 0.03 |
|  | T0🡪T2 | 2.56 (-1.78,9.30)^M^ | 1.06 (-2.96,11.27) |  |  | 0.36 (3.11) | 0.91 | 0.01 |
| walk ratio  [cm/steps/min] | T0🡪T1 | -0.01 (-0.03,0.02) | -0.01 (-0.02,0.01) | FMA* | multicollinearity | 0.01 (0.01) | 0.61 | 0.07 |
|  | T0🡪T2 | -0.01 (-0.03,0.02) | -0.01 (-0.03,0.01)^M^ |  |  | -0.002 (0.01) | 0.82 | 0.03 |

***Bold*** *& *: significant result. ° significant baseline different. ^M^ / ^L^ : medium / large effect size. ES: effect size, Bravais-Pearson correlation coefficients (r). CRR: Correct Response Rate. GVI: Gait Variability Index. OWA: Outdoor Walking Assessment. RE: random effects. TUG: Timed-up-and-go Test. TUG-Cogn: cognitive dual-task TUG. 10MWT: 10-Meter Walk Test.*

**Table S10: Baseline differences and within-group changes**

|  | | **Intention-to-treat** | | | | | **Per-protocol** | | | | |
| --- | --- | --- | --- | --- | --- | --- | --- | --- | --- | --- | --- |
| **Outcome** | **Period** | **Baseline Difference** | **Δ Intervention** | | **Δ Control** | | **Baseline Difference** | **Δ Intervention** | | **Δ Control** | |
|  |  | p-value | p-value | r | p-value | r | p-value | p-value | r | p-value | r |
| **MoCA**, total score | T0🡪T1 | 0.62 | 0.42 | 0.16 | 0.19 | 0.28 | 0.51 | 0.27 | 0.28 | 0.19 | 0.29 |
|  | T0🡪T2 |  | 0.91 | 0.02 | 0.18 | 0.28 |  | 0.92 | 0.03 | 0.18 | 0.29 |
| **SRT** |  |  |  |  |  |  |  |  |  |  |  |
| Intrinsic visual, RT | T0🡪T1 | 0.15 | 0.57 | 0.11 | 0.34 | 0.20 | 0.31 | 0.46 | 0.19 | 0.34 | 0.21 |
|  | T0🡪T2 |  | 0.07 | 0.37 | 0.50 | 0.14 |  | 0.17 | 0.35 | 0.50 | 0.15 |
| Intrinsic visual, missed | T0🡪T1 | 0.36 | > 0.99 | < 0.01 | > 0.99 | < 0.01 | > 0.99 | > 0.99 | < 0.01 | > 0.99 | < 0.01 |
|  | T0🡪T2 |  | 0.77 | 0.06 | > 0.99 | < 0.01 |  | 0.35 | 0.24 | > 0.99 | < 0.01 |
| Intrinsic visual, mistakes | T0🡪T1 | 0.52 | 0.32 | 0.20 | 0.62 | 0.10 | 0.63 | 0.18 | 0.35 | 0.62 | 0.11 |
|  | T0🡪T2 |  | 0.95 | 0.01 | 0.89 | 0.03 |  | 0.62 | 0.11 | 0.89 | 0.03 |
| CM-P visual, RT | T0🡪T1 | 0.28 | 0.02 | 0.47 | 0.40 | 0.18 | 0.62 | 0.02 | 0.59 | 0.40 | 0.18 |
|  | T0🡪T2 |  | 0.25 | 0.23 | 0.45 | 0.16 |  | 0.15 | 0.37 | 0.45 | 0.16 |
|  |  |  |  |  |  |  |  |  |  |  |  |
| CM-P visual, missed | T0🡪T1 | **0.01** | 0.10 | 0.34 | > 0.99 | < 0.01 | **0.002** | 0.07 | 0.47 | > 0.99 | < 0.01 |
|  | T0🡪T2 |  | 0.15 | 0.29 | > 0.99 | < 0.01 |  | 0.05 | 0.51 | > 0.99 | < 0.01 |
| CM-P visual, mistakes | T0🡪T1 | > 0.99 | 0.39 | 0.17 | 0.59 | 0.12 | 0.78 | 0.46 | 0.19 | 0.59 | 0.12 |
|  | T0🡪T2 |  | 0.20 | 0.26 | 0.80 | 0.05 |  | 0.28 | 0.28 | 0.80 | 0.06 |
| UM-P visual, RT | T0🡪T1 | 0.76 | 0.02 | 0.48 | 0.55 | 0.12 | 0.62 | 0.02 | 0.59 | 0.55 | 0.13 |
|  | T0🡪T2 |  | 0.16 | 0.29 | 0.15 | 0.30 |  | 0.02 | 0.61 | 0.15 | 0.31 |
| UM-P visual, missed | T0🡪T1 | **0.03** | 0.48 | 0.14 | > 0.99 | < 0.01 | **0.01** | 0.48 | 0.18 | > 0.99 | < 0.01 |
|  | T0🡪T2 |  | 0.24 | 0.24 | > 0.99 | < 0.01 |  | 0.23 | 0.31 | > 0.99 | < 0.01 |
| UM-P visual, mistakes | T0🡪T1 | **0.02** | 0.38 | 0.18 | 0.07 | 0.38 | **0.003** | 0.38 | 0.23 | 0.07 | 0.39 |
|  | T0🡪T2 |  | 0.26 | 0.23 | > 0.99 | < 0.01 |  | 0.24 | 0.31 | > 0.99 | < 0.01 |
| Intrinsic auditory, RT | T0🡪T1 | 0.32 | 0.61 | 0.10 | 0.77 | 0.06 | 0.48 | 0.57 | 0.15 | 0.77 | 0.06 |
|  | T0🡪T2 |  | 0.67 | 0.09 | 0.53 | 0.13 |  | 0.89 | 0.04 | 0.53 | 0.14 |
| Intrinsic auditory, missed | T0🡪T1 | 0.36 | 0.77 | 0.06 | > 0.99 | < 0.01 | 0.38 | 0.77 | 0.07 | > 0.99 | < 0.01 |
|  | T0🡪T2 |  | 0.85 | 0.04 | > 0.99 | < 0.01 |  | 0.85 | 0.05 | > 0.99 | < 0.01 |
| Intrinsic auditory, mistakes | T0🡪T1 | 0.31 | 0.90 | 0.03 | 0.73 | 0.07 | 0.11 | 0.81 | 0.06 | 0.73 | 0.08 |
|  | T0🡪T2 |  | 0.56 | 0.12 | 0.38 | 0.19 |  | 0.68 | 0.10 | 0.38 | 0.19 |
| CM-P auditory, RT | T0🡪T1 | 0.64 | 0.36 | 0.18 | 0.05 | 0.42 | 0.90 | 0.27 | 0.29 | 0.05 | 0.43 |
|  | T0🡪T2 |  | 0.24 | 0.24 | 0.06 | 0.40 |  | 0.08 | 0.45 | 0.06 | 0.41 |
| CM-P auditory, missed | T0🡪T1 | 0.58 | 0.59 | 0.11 | 0.27 | 0.24 | 0.89 | 0.77 | 0.08 | 0.27 | 0.24 |
|  | T0🡪T2 |  | 0.58 | 0.11 | 0.35 | 0.20 |  | 0.17 | 0.35 | 0.35 | 0.20 |
| CM-P auditory, mistakes | T0🡪T1 | 0.52 | > 0.99 | < 0.01 | 0.17 | 0.29 | 0.29 | > 0.99 | < 0.01 | 0.17 | 0.30 |
|  | T0🡪T2 |  | 0.40 | 0.17 | 0.33 | 0.21 |  | 0.60 | 0.13 | 0.33 | 0.21 |
| UM-P auditory, RT | T0🡪T1 | 0.50 | 0.18 | 0.28 | 0.19 | 0.28 | 0.89 | 0.12 | 0.40 | 0.19 | 0.29 |
|  | T0🡪T2 |  | 0.16 | 0.29 | 0.03 | 0.46 |  | 0.03 | 0.55 | 0.03 | 0.47 |
| UM-P auditory, missed | T0🡪T1 | 0.07 | 0.48 | 0.14 | 0.37 | 0.19 | 0.11 | 0.42 | 0.21 | 0.37 | 0.20 |
|  | T0🡪T2 |  | **0.04** | 0.42 | 0.59 | 0.12 |  | **0.04** | 0.54 | 0.59 | 0.12 |
| UM-P auditory, mistakes | T0🡪T1 | 0.80 | 0.89 | 0.03 | 0.34 | 0.20 | 0.45 | > 0.99 | < 0.01 | 0.34 | 0.21 |
|  | T0🡪T2 |  | 0.31 | 0.21 | 0.82 | 0.05 |  | 0.19 | 0.34 | 0.82 | 0.05 |
| **TMT** |  |  |  |  |  |  |  |  |  |  |  |
| -A, time | T0🡪T1 | 0.33 | 0.43 | 0.16 | 0.24 | 0.25 | 0.14 | 0.90 | 0.03 | 0.24 | 0.26 |
|  | T0🡪T2 |  | 0.16 | 0.28 | 0.18 | 0.29 |  | 0.08 | 0.45 | 0.18 | 0.29 |
| -A, mistakes | T0🡪T1 | **< 0.01** | 0.67 | 0.09 | 0.72 | 0.08 | 0.29 | 0.77 | 0.08 | 0.72 | 0.08 |
|  | T0🡪T2 |  | 0.98 | 0.01 | 0.47 | 0.15 |  | 0.94 | 0.02 | 0.47 | 0.16 |
| -B, time | T0🡪T1 | 0.71 | 0.56 | 0.12 | 0.70 | 0.08 | 0.86 | 0.71 | 0.09 | 0.71 | 0.08 |
|  | T0🡪T2 |  | 0.59 | 0.11 | 0.37 | 0.19 |  | 0.33 | 0.25 | 0.37 | 0.19 |
| -B, mistakes | T0🡪T1 | 0.45 | 0.91 | 0.02 | 0.48 | 0.15 | 0.79 | 0.43 | 0.20 | 0.48 | 0.15 |
|  | T0🡪T2 |  | 0.78 | 0.06 | 0.68 | 0.09 |  | 0.33 | 0.25 | 0.68 | 0.09 |
| B-A ratio | T0🡪T1 | 0.48 | 0.67 | 0.09 | 0.60 | 0.11 | 0.10 | 0.81 | 0.06 | 0.61 | 0.11 |
|  | T0🡪T2 |  | 0.48 | 0.14 | 0.89 | 0.03 |  | 0.24 | 0.30 | 0.89 | 0.03 |
| **Stroop** |  |  |  |  |  |  |  |  |  |  |  |
| Reading baseline, RT | T0🡪T1 | 0.61 | 0.66 | 0.09 | 0.97 | 0.01 | 0.35 | 0.36 | 0.25 | 0.97 | 0.01 |
|  | T0🡪T2 |  | **0.03** | 0.44 | 0.85 | 0.04 |  | 0.07 | 0.48 | 0.84 | 0.04 |
| Reading baseline, mistakes | T0🡪T1 | 0.26 | 0.39 | 0.18 | 0.24 | 0.25 | 0.76 | 0.39 | 0.18 | 0.24 | 0.24 |
|  | T0🡪T2 |  | 0.28 | 0.23 | 0.34 | 0.20 |  | 0.67 | 0.09 | 0.34 | 0.20 |
| Reading interference, RT | T0🡪T1 | 0.15 | 0.34 | 0.20 | **0.02** | 0.49 | **0.02** | 0.24 | 0.31 | 0.02 | 0.50 |
|  | T0🡪T2 |  | 0.46 | 0.16 | 0.13 | 0.32 |  | > 0.99 | < 0.01 | 0.13 | 0.33 |
| Reading interference, mistakes | T0🡪T1 | 0.12 | 0.25 | 0.24 | 0.07 | 0.39 | 0.25 | 0.17 | 0.36 | 0.07 | 0.40 |
|  | T0🡪T2 |  | 0.55 | 0.12 | 0.07 | 0.39 |  | 0.61 | 0.14 | 0.07 | 0.40 |
| R. interference tendency | T0🡪T1 | 0.10 | 0.40 | 0.18 | 0.06 | 0.40 | **0.02** | 0.16 | 0.38 | 0.06 | 0.41 |
|  | T0🡪T2 |  | 0.84 | 0.04 | 0.18 | 0.29 |  | 0.78 | 0.08 | 0.18 | 0.30 |
| Naming baseline, RT | T0🡪T1 | 0.72 | 0.41 | 0.17 | 0.47 | 0.16 | 0.52 | 0.16 | 0.38 | 0.47 | 0.16 |
|  | T0🡪T2 |  | 0.64 | 0.10 | 0.16 | 0.30 |  | 0.53 | 0.17 | 0.16 | 0.31 |
| Naming baseline, mistakes | T0🡪T1 | 0.99 | 0.39 | 0.18 | 0.61 | 0.11 | 0.42 | > 0.99 | < 0.01 | 0.61 | 0.11 |
|  | T0🡪T2 |  | 0.93 | 0.02 | 0.82 | 0.05 |  | > 0.99 | < 0.01 | 0.82 | 0.05 |
| Naming interference, RT | T0🡪T1 | 0.76 | 0.95 | 0.01 | > 0.99 | < 0.01 | 0.73 | 0.86 | 0.05 | > 0.99 | < 0.01 |
|  | T0🡪T2 |  | 0.51 | 0.14 | 0.42 | 0.17 |  | 0.54 | 0.16 | 0.43 | 0.17 |
| Naming interference, mistakes | T0🡪T1 | 0.89 | 0.22 | 0.26 | 0.97 | 0.01 | 0.86 | 0.33 | 0.26 | 0.97 | 0.01 |
|  | T0🡪T2 |  | 0.72 | 0.07 | 0.75 | 0.07 |  | 0.91 | 0.03 | 0.75 | 0.07 |
| N. interference tendency | T0🡪T1 | 0.32 | 0.73 | 0.07 | 0.63 | 0.10 | 0.30 | 0.63 | 0.13 | 0.63 | 0.10 |
|  | T0🡪T2 |  | 0.64 | 0.10 | 0.60 | 0.11 |  | 0.90 | 0.03 | 0.61 | 0.11 |
| **NBT** |  |  |  |  |  |  |  |  |  |  |  |
| RT correct | T0🡪T1 | 0.93 | 0.67 | 0.09 | 0.40 | 0.18 | 0.59 | 0.62 | 0.13 | 0.41 | 0.18 |
|  | T0🡪T2 |  | 0.31 | 0.21 | 0.09 | 0.36 |  | 0.62 | 0.13 | 0.09 | 0.37 |
| correct | T0🡪T1 | 0.34 | 0.39 | 0.18 | 0.96 | 0.01 | 0.66 | 0.75 | 0.09 | 0.96 | 0.01 |
|  | T0🡪T2 |  | 0.35 | 0.20 | 0.82 | 0.05 |  | 0.06 | 0.49 | 0.82 | 0.05 |
| omissions | T0🡪T1 | 0.34 | 0.26 | 0.23 | 0.94 | 0.02 | 0.66 | 0.47 | 0.19 | 0.94 | 0.02 |
|  | T0🡪T2 |  | 0.35 | 0.20 | 0.82 | 0.05 |  | 0.06 | 0.49 | 0.82 | 0.05 |
| mistakes | T0🡪T1 | 0.63 | **0.03** | 0.45 | 0.10 | 0.35 | 0.69 | 0.03 | 0.59 | 0.10 | 0.36 |
|  | T0🡪T2 |  | **0.02** | 0.48 | 0.15 | 0.31 |  | 0.01 | 0.67 | 0.15 | 0.32 |
| **MRT** |  |  |  |  |  |  |  |  |  |  |  |
| RT correct | T0🡪T1 | 0.97 | 0.37 | 0.19 | 0.47 | 0.16 | 0.29 | 0.95 | 0.02 | 0.47 | 0.16 |
|  | T0🡪T2 |  | 0.60 | 0.11 | 0.19 | 0.28 |  | 0.95 | 0.02 | 0.19 | 0.29 |
| correct | T0🡪T1 | 0.32 | **0.01** | 0.52 | 0.08 | 0.37 | 0.46 | **0.02** | 0.64 | 0.08 | 0.38 |
|  | T0🡪T2 |  | **0.01** | 0.54 | 0.07 | 0.39 |  | **0.01** | 0.66 | 0.07 | 0.40 |
| accuracy | T0🡪T1 | 0.32 | **0.01** | 0.53 | 0.07 | 0.38 | 0.46 | **0.02** | 0.64 | 0.07 | 0.39 |
|  | T0🡪T2 |  | **0.01** | 0.57 | **0.04** | 0.44 |  | **0.01** | 0.67 | **0.04** | 0.45 |
| **SIS** |  |  |  |  |  |  |  |  |  |  |  |
| Total score | T0🡪T1 | > 0.99 | 0.06 | 0.41 | 0.31 | 0.22 | 0.39 | 0.17 | 0.36 | 0.31 | 0.22 |
|  | T0🡪T2 |  | 0.07 | 0.40 | 0.86 | 0.04 |  | 0.71 | 0.10 | 0.86 | 0.04 |
| Strength | T0🡪T1 | 0.17 | 0.82 | 0.05 | 0.53 | 0.13 | **0.04** | 0.39 | 0.22 | 0.53 | 0.14 |
|  | T0🡪T2 |  | 0.69 | 0.09 | 0.34 | 0.20 |  | 0.08 | 0.46 | 0.34 | 0.21 |
| Memory | T0🡪T1 | 0.69 | 0.30 | 0.23 | 0.06 | 0.40 | 0.70 | 0.62 | 0.13 | 0.06 | 0.41 |
|  | T0🡪T2 |  | 0.62 | 0.23 | 0.66 | 0.09 |  | 0.40 | 0.22 | 0.66 | 0.10 |
| Emotions | T0🡪T1 | 0.42 | 0.74 | 0.07 | 0.06 | 0.40 | 0.90 | 0.94 | 0.02 | 0.06 | 0.41 |
|  | T0🡪T2 |  | 0.27 | 0.24 | 0.82 | 0.05 |  | 0.40 | 0.22 | 0.82 | 0.05 |
| Communication | T0🡪T1 | 0.81 | 0.11 | 0.35 | 0.50 | 0.14 | 0.64 | 0.16 | 0.37 | 0.50 | 0.15 |
|  | T0🡪T2 |  | 0.07 | 0.39 | > 0.99 | < 0.01 |  | 0.33 | 0.25 | > 0.99 | < 0.01 |
| Activity | T0🡪T1 | 0.64 | 0.12 | 0.34 | 0.47 | 0.15 | 0.42 | 0.09 | 0.43 | 0.47 | 0.16 |
|  | T0🡪T2 |  | 0.33 | 0.21 | 0.44 | 0.17 |  | 0.81 | 0.06 | 0.44 | 0.17 |
| Mobility | T0🡪T1 | 0.45 | 0.17 | 0.30 | 0.20 | 0.28 | 0.58 | 0.28 | 0.28 | 0.20 | 0.28 |
|  | T0🡪T2 |  | **0.01** | 0.54 | 0.69 | 0.09 |  | **0.04** | 0.52 | 0.69 | 0.09 |
|  |  |  |  |  |  |  |  |  |  |  |  |
| Hand | T0🡪T1 | 0.59 | 0.26 | 0.25 | 0.26 | 0.24 | 0.81 | 0.40 | 0.22 | 0.26 | 0.25 |
|  | T0🡪T2 |  | 0.94 | 0.02 | 0.15 | 0.31 |  | 0.20 | 0.33 | 0.15 | 0.31 |
| Participation | T0🡪T1 | 0.78 | 0.12 | 0.34 | 0.82 | 0.05 | 0.39 | 0.08 | 0.45 | 0.82 | 0.05 |
|  | T0🡪T2 |  | 0.45 | 0.17 | 0.33 | 0.21 |  | 0.55 | 0.15 | 0.33 | 0.21 |
| Recovery | T0🡪T1 | 0.84 | 0.23 | 0.26 | > 0.99 | < 0.01 | 0.37 | 0.07 | 0.47 | > 0.99 | < 0.01 |
|  | T0🡪T2 |  | > 0.99 | < 0.01 | 0.83 | 0.05 |  | 0.40 | 0.22 | 0.83 | 0.05 |
| **TUG** |  |  |  |  |  |  |  |  |  |  |  |
| time | T0🡪T1 | 0.61 | **0.01** | 0.53 | **0.03** | 0.45 | 0.72 | **0.02** | 0.60 | **0.03** | 0.46 |
|  | T0🡪T2 |  | 0.43 | 0.16 | 0.14 | 0.32 |  | 0.37 | 0.23 | 0.14 | 0.32 |
| **TUG-Cogn** |  |  |  |  |  |  |  |  |  |  |  |
| time | T0🡪T1 | 0.09 | **0.04** | 0.43 | **0.03** | 0.47 | 0.09 | 0.09 | 0.45 | **0.03** | 0.48 |
|  | T0🡪T2 |  | 0.06 | 0.40 | **0.01** | 0.57 |  | 0.14 | 0.39 | **0.01** | 0.58 |
| motor Dual-Task Effect | T0🡪T1 | **0.03** | 0.30 | 0.22 | 0.09 | 0.63 | **0.02** | 0.29 | 0.28 | 0.09 | 0.37 |
|  | T0🡪T2 |  | 0.06 | 0.40 | **0.01** | 0.53 |  | 0.20 | 0.35 | **0.01** | 0.56 |
| CRR single-task | T0🡪T1 | 0.06 | **0.001** | 0.67 | **0.03** | 0.46 | **0.02** | **0.01** | 0.75 | **0.03** | 0.47 |
|  | T0🡪T2 |  | **0.001** | 0.71 | **0.001** | 0.73 |  | **0.003** | 0.78 | **0.0001** | 0.81 |
| CRR dual-task | T0🡪T1 | 0.28 | **0.04** | 0.44 | 0.64 | 0.10 | 0.06 | 0.11 | 0.43 | 0.65 | 0.10 |
|  | T0🡪T2 |  | **0.01** | 0.52 | 0.31 | 0.21 |  | 0.05 | 0.51 | 0.32 | 0.22 |
| **10MWT** |  |  |  |  |  |  |  |  |  |  |  |
| time | T0🡪T1 | 0.55 | 0.07 | 0.37 | 0.14 | 0.31 | 0.54 | 0.49 | 0.18 | 0.14 | 0.32 |
|  | T0🡪T2 |  | 0.55 | 0.12 | 0.26 | 0.24 |  | 0.72 | 0.09 | 0.26 | 0.24 |
| speed | T0🡪T1 | 0.56 | 0.09 | 0.35 | 0.19 | 0.28 | 0.41 | 0.89 | 0.04 | 0.19 | 0.29 |
|  | T0🡪T2 |  | 0.04 | 0.43 | 0.19 | 0.28 |  | 0.22 | 0.32 | 0.19 | 0.29 |
| cadence | T0🡪T1 | 0.21 | 0.29 | 0.22 | 0.24 | 0.25 | 0.25 | 0.89 | 0.04 | 0.25 | 0.25 |
|  | T0🡪T2 |  | 0.06 | 0.39 | **0.04** | 0.44 |  | 0.17 | 0.35 | **0.04** | 0.46 |
| stride length affected | T0🡪T1 | 0.53 | **0.02** | 0.48 | 0.06 | 0.39 | 0.43 | 0.15 | 0.37 | 0.06 | 0.40 |
|  | T0🡪T2 |  | **0.03** | 0.43 | 0.09 | 0.36 |  | 0.17 | 0.35 | 0.09 | 0.37 |
| stride length unaffected | T0🡪T1 | 0.39 | 0.10 | 0.33 | 0.22 | 0.26 | 0.36 | 0.76 | 0.06 | 0.19 | 0.27 |
|  | T0🡪T2 |  | 0.12 | 0.32 | > 0.99 | < 0.01 |  | 0.50 | 0.14 | > 0.99 | < 0.01 |
| stride time affected | T0🡪T1 | 0.29 | 0.40 | 0.17 | 0.22 | 0.26 | 0.32 | 0.93 | 0.02 | 0.20 | 0.28 |
|  | T0🡪T2 |  | 0.05 | 0.40 | 0.14 | 0.31 |  | 0.15 | 0.37 | 0.15 | 0.32 |
| stride time unaffected | T0🡪T1 | 0.19 | 0.24 | 0.24 | 0.07 | 0.39 | 0.36 | 0.85 | 0.05 | 0.06 | 0.41 |
|  | T0🡪T2 |  | **0.01** | 0.56 | 0.05 | 0.41 |  | **0.01** | 0.64 | 0.05 | 0.43 |
| double-support time | T0🡪T1 | 0.47 | 0.64 | 0.10 | 0.16 | 0.30 | 0.40 | 0.60 | 0.14 | 0.17 | 0.30 |
|  | T0🡪T2 |  | 0.53 | 0.13 | 0.24 | 0.25 |  | 0.86 | 0.05 | 0.24 | 0.25 |
| stance time affected | T0🡪T1 | 0.29 | 0.98 | 0.004 | 0.51 | 0.14 | 0.25 | 0.41 | 0.21 | 0.52 | 0.14 |
|  | T0🡪T2 |  | 0.73 | 0.07 | 0.26 | 0.24 |  | 0.81 | 0.06 | 0.26 | 0.25 |
| stance time unaffected | T0🡪T1 | 0.22 | 0.47 | 0.15 | 0.08 | 0.37 | 0.24 | 0.68 | 0.11 | 0.08 | 0.38 |
|  | T0🡪T2 |  | 0.51 | 0.14 | 0.12 | 0.33 |  | > 0.99 | < 0.01 | 0.12 | 0.34 |
| swing time affected | T0🡪T1 | 0.29 | 0.98 | 0.004 | 0.51 | 0.14 | 0.25 | 0.41 | 0.21 | 0.52 | 0.14 |
|  | T0🡪T2 |  | 0.73 | 0.07 | 0.26 | 0.24 |  | 0.81 | 0.06 | 0.26 | 0.25 |
| swing time unaffected | T0🡪T1 | 0.27 | 0.47 | 0.15 | 0.10 | 0.35 | 0.28 | 0.68 | 0.11 | 0.11 | 0.35 |
|  | T0🡪T2 |  | 0.51 | 0.14 | 0.19 | 0.28 |  | > 0.99 | < 0.01 | 0.19 | 0.29 |
| swing width affected | T0🡪T1 | 0.90 | 0.56 | 0.12 | 0.44 | 0.16 | 0.77 | 0.48 | 0.18 | 0.45 | 0.16 |
|  | T0🡪T2 |  | 0.84 | 0.04 | 0.65 | 0.10 |  | 0.68 | 0.11 | 0.65 | 0.10 |
|  |  |  |  |  |  |  |  |  |  |  |  |
| swing width unaffected | T0🡪T1 | 0.05 | 0.07 | 0.37 | 0.47 | 0.16 | 0.04 | 0.19 | 0.34 | 0.47 | 0.16 |
|  | T0🡪T2 |  | 0.10 | 0.34 | 0.89 | 0.03 |  | 0.30 | 0.27 | 0.89 | 0.03 |
| asymmetry | T0🡪T1 | 0.11 | 0.51 | 0.13 | 0.29 | 0.23 | 0.03 | 0.93 | 0.02 | 0.29 | 0.23 |
|  | T0🡪T2 |  | 0.82 | 0.05 | 0.10 | 0.35 |  | 0.30 | 0.27 | 0.10 | 0.36 |
| GVI affected | T0🡪T1 | 0.15 | 0.29 | 0.22 | 0.08 | 0.37 | 0.36 | 0.72 | 0.09 | 0.08 | 0.38 |
|  | T0🡪T2 |  | 0.89 | 0.03 | 0.28 | 0.23 |  | 0.63 | 0.13 | 0.29 | 0.23 |
| GVI unaffected | T0🡪T1 | 0.35 | 0.25 | 0.23 | 0.20 | 0.27 | 0.25 | 0.85 | 0.05 | 0.20 | 0.28 |
|  | T0🡪T2 |  | 0.22 | 0.25 | 0.30 | 0.22 |  | 0.06 | 0.49 | 0.30 | 0.22 |
| walk ratio | T0🡪T1 | 0.84 | 0.32 | 0.20 | 0.93 | 0.02 | 0.53 | 0.72 | 0.09 | 0.93 | 0.02 |
|  | T0🡪T2 |  | 0.82 | 0.05 | 0.19 | 0.28 |  | 0.33 | 0.25 | 0.19 | 0.29 |
| **10MWT fast** |  |  |  |  |  |  |  |  |  |  |  |
| time | T0🡪T1 | 0.44 | **0.01** | 0.52 | **0.04** | 0.45 | 0.31 | **0.04** | 0.54 | **0.04** | 0.46 |
|  | T0🡪T2 |  | **0.05** | 0.43 | 0.29 | 0.23 |  | 0.07 | 0.48 | 0.29 | 0.24 |
| **OWA** |  |  |  |  |  |  |  |  |  |  |  |
| speed | T0🡪T1 | 0.43 | 0.12 | 0.33 | 0.32 | 0.22 | 0.74 | 0.75 | 0.08 | 0.32 | 0.22 |
|  | T0🡪T2 |  | 0.14 | 0.31 | > 0.99 | < 0.01 |  | 0.64 | 0.12 | > 0.99 | < 0.01 |
| cadence | T0🡪T1 | 0.71 | **0.01** | 0.55 | 0.86 | 0.04 | 0.73 | 0.08 | 0.45 | 0.87 | 0.04 |
|  | T0🡪T2 |  | **0.05** | 0.42 | 0.43 | 0.17 |  | 0.09 | 0.44 | 0.44 | 0.17 |
| stride length affected | T0🡪T1 | 0.74 | 0.36 | 0.19 | 0.41 | 0.18 | 0.91 | 0.76 | 0.08 | 0.42 | 0.18 |
|  | T0🡪T2 |  | 0.37 | 0.19 | 0.84 | 0.04 |  | 0.78 | 0.07 | 0.84 | 0.04 |
| stride length unaffected | T0🡪T1 | 0.95 | 0.70 | 0.08 | 0.21 | 0.28 | 0.68 | 0.67 | 0.11 | 0.21 | 0.28 |
|  | T0🡪T2 |  | 0.83 | 0.05 | 0.61 | 0.11 |  | 0.71 | 0.09 | 0.61 | 0.11 |
| stride time affected | T0🡪T1 | 0.77 | **0.04** | 0.44 | 0.93 | 0.02 | 0.83 | 0.23 | 0.31 | 0.93 | 0.02 |
|  | T0🡪T2 |  | **0.01** | 0.51 | 0.25 | 0.25 |  | 0.06 | 0.49 | 0.26 | 0.25 |
| stride time unaffected | T0🡪T1 | 0.70 | **0.05** | 0.42 | 0.91 | 0.02 | 0.76 | 0.30 | 0.27 | 0.91 | 0.02 |
|  | T0🡪T2 |  | **0.03** | 0.46 | 0.28 | 0.24 |  | 0.06 | 0.49 | 0.28 | 0.24 |
| double-support time | T0🡪T1 | 0.68 | 0.66 | 0.09 | 0.93 | 0.02 | 0.99 | 0.54 | 0.16 | 0.93 | 0.02 |
|  | T0🡪T2 |  | 0.13 | 0.32 | 0.48 | 0.15 |  | 0.08 | 0.45 | 0.49 | 0.15 |
| stance time affected | T0🡪T1 | 0.28 | 0.46 | 0.15 | 0.85 | 0.04 | 0.40 | 0.33 | 0.25 | 0.85 | 0.04 |
|  | T0🡪T2 |  | 0.11 | 0.33 | 0.46 | 0.16 |  | 0.08 | 0.45 | 0.47 | 0.16 |
| stance time unaffected | T0🡪T1 | 0.98 | 0.95 | 0.01 | 0.63 | 0.10 | 0.57 | 0.71 | 0.09 | 0.64 | 0.10 |
|  | T0🡪T2 |  | 0.54 | 0.13 | 0.47 | 0.16 |  | 0.24 | 0.30 | 0.47 | 0.16 |
| swing time affected | T0🡪T1 | 0.28 | 0.46 | 0.15 | 0.85 | 0.04 | 0.40 | 0.33 | 0.25 | 0.85 | 0.04 |
|  | T0🡪T2 |  | 0.11 | 0.33 | 0.46 | 0.16 |  | 0.08 | 0.45 | 0.47 | 0.16 |
| swing time unaffected | T0🡪T1 | 0.98 | 0.95 | 0.01 | 0.63 | 0.10 | 0.57 | 0.71 | 0.09 | 0.64 | 0.10 |
|  | T0🡪T2 |  | 0.54 | 0.13 | 0.47 | 0.16 |  | 0.24 | 0.30 | 0.47 | 0.16 |
| swing width affected | T0🡪T1 | 0.72 | 0.49 | 0.15 | 0.30 | 0.23 | 0.69 | 0.95 | 0.02 | 0.30 | 0.23 |
|  | T0🡪T2 |  | 0.34 | 0.20 | 0.08 | 0.38 |  | 0.66 | 0.11 | 0.08 | 0.39 |
| swing width unaffected | T0🡪T1 | 0.28 | 0.37 | 0.19 | **0.01** | 0.58 | 0.24 | 0.51 | 0.17 | **0.01** | 0.60 |
|  | T0🡪T2 |  | **0.01** | 0.52 | 0.13 | 0.33 |  | 0.07 | 0.47 | 0.13 | 0.34 |
| asymmetry | T0🡪T1 | 0.20 | 0.09 | 0.35 | 0.32 | 0.22 | 0.15 | 0.19 | 0.34 | 0.32 | 0.22 |
|  | T0🡪T2 |  | 0.60 | 0.11 | 0.37 | 0.20 |  | 0.90 | 0.03 | 0.37 | 0.20 |
| GVI affected | T0🡪T1 | 0.92 | 0.97 | 0.01 | 0.68 | 0.09 | 0.61 | 0.43 | 0.21 | 0.50 | 0.15 |
|  | T0🡪T2 |  | 0.68 | 0.09 | 0.87 | 0.04 |  | 0.50 | 0.17 | 0.68 | 0.09 |
| GVI unaffected | T0🡪T1 | 0.63 | 0.51 | 0.14 | 0.98 | 0.004 | 0.93 | 0.58 | 0.14 | 0.73 | 0.08 |
|  | T0🡪T2 |  | 0.16 | 0.30 | 0.19 | 0.29 |  | 0.19 | 0.34 | 0.29 | 0.23 |
| walk ratio | T0🡪T1 | 0.78 | 0.38 | 0.18 | 0.41 | 0.18 | 0.56 | 0.27 | 0.29 | 0.42 | 0.18 |
|  | T0🡪T2 |  | 0.73 | 0.07 | 0.09 | 0.36 |  | 0.50 | 0.17 | 0.10 | 0.37 |

**Table S11: Intervention and Further Activity Outcomes**

| **Type / Period** | **Outcome** | | **Intervention Group** | **Control Group** | **P** |
| --- | --- | --- | --- | --- | --- |
| **T0-T1** |  | | (N=21) | (N=21) |  |
| **Intervention outcomes** | Compliance | [%] | 89.8 (13.14), range: 41.7-100 | - | - |
|  | Adherence | [%] | 89.1 (13.54), range: 39.0-100 | - | - |
| **Usual care** | Usual care, total | time (min / week) | 5.0 (0.0,39.17) | 35.0 (0.0,73.75) | 0.29 |
|  | Physical therapy, intense | frequency (sessions / week) | 0.0 (0.0,0.0) | 0.0 (0.0,0.5) | 0.13 |
|  |  | time (min / week) | 0.0 (0.0,0.0) | 0.0 (0.0,6.25) | 0.15 |
|  | Physical therapy, moderate | frequency (sessions / week) | 0.0 (0.0,0.5) | 0.0 (0.0,1.0) | 0.57 |
|  |  | time (min / week) | 0.0 (0.0,27.92) | 2.50 (0.0, 25.0) | 0.74 |
|  | Cognitive therapy | frequency (sessions / week) | 0.0 (0.0,0.0) | 0.0 (0.0,0.0) | 0.40 |
|  |  | time (min / week) | 0.0 (0.0,0.0) | 0.0 (0.0,5.0) | 0.29 |
| **Leisure activities** | Physical activity, intense | frequency (sessions / week) | 0.0 (0.0,0.0) | 0.0 (0.0,1.0) | 0.19 |
|  |  | time (min / week) | 5.0 (0.0, 26.25) | 12.5 (0.0,66.67) | 0.29 |
|  | Physical activity, moderate | frequency (sessions / week) | 3.5 (2.0,6.0) | 4.5 (2.5,6.0) | 0.57 |
|  |  | time (min / week) | 279.25 (107.50,442.50) | 283.75 (173.75,727.50) | 0.46 |
|  | Sedentary time | (hours/day) | 7.25 (5.79,9.92) | 6.83 (5.38,9.92) | 0.77 |
|  | Cognitive activity, intense | frequency (sessions / week) | 0.0 (0.0,0.0) | 0.0 (0.0,0.0) | 0.75 |
|  |  | time (min / week) | 0.0 (0.0,0.0) | 0.0 (0.0,0.0) | 0.78 |
|  | Cognitive activity, moderate | frequency (sessions / week) | 4.0 (1.5,6.0) | 2.5 (0.5,4.0) | 0.29 |
|  |  | time (min / week) | 250.83 (107.17,344.17) | 197.50 (7.50,247.50) | 0.22 |
| **T1-T2** |  |  | (N=20) | (N=18) |  |
| **Usual care** | Usual care, total | time (min / week) | 25.83 (0.0,45.0) | 21.25 (0.0,47.50) | 0.90 |
|  | Physical therapy, intense | frequency (sessions / week) | 0.0 (0.0,0.0) | 0.0 (0.0,0.0) | 0.63 |
|  |  | time (min / week) | 0.0 (0.0,1.67) | 0.0 (0.0,5.0) | 0.51 |
|  | Physical therapy, moderate | frequency (sessions / week) | 0.5 (0.0,1.0) | 0.0 (0.0,0.50) | 0.57 |
|  |  | time (min / week) | 9.17 (0.0,37.50) | 2.50 (0.0,21.25) | 0.50 |
|  | Cognitive therapy | frequency (sessions / week) | 0.0 (0.0,0.0) | 0.0 (0.0,0.0) | 0.55 |
|  |  | time (min / week) | 0.0 (0.0,10.0) | 0.0 (0.0,3.44) | 0.71 |
| **Leisure activities** | Physical activity, intense | frequency (sessions / week) | 0.0 (0.0,0.0) | 0.0 (0.0,1.0) | 0.33 |
|  |  | time (min / week) | 0.0 (0.0,7.92) | 0.0 (0.0,38.33) | 0.34 |
|  | Physical activity, moderate | frequency (sessions / week) | 4.50 (3.0,5.50) | 5.0 (2.50,6.50) | 0.82 |
|  |  | time (min / week) | 289.17 (148.33,502.08) | 300.0 (173.75,653.75) | 0.49 |
|  | Sedentary time | (hours/day) | 6.42 (5.83,11.0) | 6.83 (5.75,9.92) | 0.80 |
|  | Cognitive activity, intense | frequency (sessions / week) | 0.0 (0.0,0.0) | 0.0 (0.0,0.0) | 0.49 |
|  |  | time (min / week) | 0.0 (0.0,0.0) | 0.0 (0.0,3.75) | 0.44 |
|  | Cognitive activity, moderate | frequency (sessions / week) | 4.0 (3.0,6.0) | 3.0 (0.50,4.0) | 0.27 |
|  |  | time (min / week) | 243.17 (118.75,378.75) | 192.92 (5.0,270.0) | 0.28 |
